# High nitrate levels in skeletal muscle contribute to nitric oxide generation via a nitrate/nitrite reductive pathway in mice that lack the nNOS enzyme

**DOI:** 10.3389/fphys.2024.1352242

**Published:** 2024-05-09

**Authors:** Supranee Upanan, Jeeyoung Lee, Khalid J. Tunau-Spencer, Praveen K. Rajvanshi, Elizabeth C. Wright, Constance T. Noguchi, Alan N. Schechter

**Affiliations:** ^1^ Molecular Medicine Branch, National Institute of Diabetes and Digestive and Kidney Diseases, National Institutes of Health, Bethesda, MD, United States; ^2^ Office of the Director, National Institute of Diabetes and Digestive and Kidney Diseases, National Institutes of Health, Bethesda, MD, United States

**Keywords:** nitric oxide, nitrate, nitric oxide synthases, nitrate/nitrite reductive pathway, skeletal muscle, neuronal nitric oxide synthase, neuronal nitric oxide synthase knockout mice (nNOS−/−), xanthine oxidoreductase

## Abstract

**Introduction:**

Nitric oxide (NO) is a vasodilator gas that plays a critical role in mitochondrial respiration and skeletal muscle function. NO is endogenously generated by NO synthases: neuronal NO synthase (nNOS), endothelial NO synthase (eNOS), or inducible NO synthase (iNOS). NO in skeletal muscle is partly generated by nNOS, and nNOS deficiency can contribute to muscular dystrophic diseases. However, we and others discovered an alternative nitrate/nitrite reductive pathway for NO generation: nitrate to nitrite to NO. We hypothesized that nitrate supplementation would increase nitrate accumulation in skeletal muscle and promote a nitrate/nitrite reductive pathway for NO production to compensate for the loss of nNOS in skeletal muscle.

**Methods:**

Wild-type (WT) and genetic nNOS knockout (nNOS−/−) mice were fed normal chow (386.9 nmol/g nitrate) and subjected to three treatments: high-nitrate water (1 g/L sodium nitrate for 7 days), low-nitrate diet (46.8 nmol/g nitrate for 7 days), and low-nitrate diet followed by high-nitrate water for 7 days each.

**Results:**

High-nitrate water supplementation exhibited a greater and more significant increase in nitrate levels in skeletal muscle and blood in nNOS−/− mice than in WT mice. A low-nitrate diet decreased blood nitrate and nitrite levels in both WT and nNOS−/− mice. WT and nNOS−/− mice, treated with low-nitrate diet, followed by high-nitrate water supplementation, showed a significant increase in nitrate levels in skeletal muscle and blood, analogous to the increases observed in nNOS−/− mice supplemented with high-nitrate water. In skeletal muscle of nNOS−/− mice on high-nitrate water supplementation, on low-nitrate diet, and in low–high nitrate treatment, the loss of nNOS resulted in a corresponding increase in the expression of nitrate/nitrite reductive pathway-associated nitrate transporters [sialin and chloride channel 1 (CLC1)] and nitrate/nitrite reductase [xanthine oxidoreductase (XOR)] but did not show a compensatory increase in iNOS or eNOS protein and eNOS activation activity [p-eNOS (Ser1177)].

**Discussion:**

These findings suggest that a greater increase in nitrate levels in skeletal muscle of nNOS−/− mice on nitrate supplementation results from reductive processes to increase NO production with the loss of nNOS in skeletal muscle.

## 1 Introduction

Nitric oxide (NO), a free-radical gas, is well-known as a vasodilator with an essential role in signaling and regulatory functions in mitochondrial bioenergetics (Nisoli and Carruba, 2006; Lorin et al., 2014; Pappas et al., 2023). In skeletal muscle, it is a versatile signaling molecule in muscle development and function, for example, to attenuate muscle force generation and regulate proper blood and oxygen delivery to active muscle during exercise (Dyakova et al., 2015; Tan et al., 2023). NO was first discovered to be endogenously synthesized by the oxidation of the amino acid L-arginine to NO and L-citrulline, catalyzed by three NO synthases: endothelial NO synthase (eNOS), neuronal NO synthase (nNOS), and inducible NO synthase (iNOS) (Lorin et al., 2014; Dyakova et al., 2015; Tejero et al., 2019). In skeletal muscle, NO is generated mostly by nNOS, and defective NO signaling can impair skeletal muscle growth and performance by involving mitochondrial dysregulation (Wadley and McConell, 2007; De Palma et al., 2014; Pappas et al., 2023). In addition, nNOS deficiency caused by gene mutation can contribute to muscular dystrophic diseases with progressive muscle wasting, such as Duchenne and Becker muscular dystrophies (Percival et al., 2008; Tidball and Wehling-Henricks, 2014; Esposito and Carsana, 2019).

In addition to NOS pathways, NO can be generated via an alternative nitrate–nitrite–NO pathway or a nitrate/nitrite reductive pathway, where nitrate (NO_3_^−^) is reduced to nitrite (NO_2_^−^) and then to NO (Kozlov et al., 1999; Lundberg et al., 2008). The nitrate–nitrite–NO pathway has been reported to be involved in the regulation of blood flow and cell metabolism, especially during hypoxia or exercise (Larsen et al., 2006; Sparacino-Watkins et al., 2012; Roberts et al., 2015; Carlstrom and Montenegro, 2019; McNally et al., 2020; Piknova et al., 2022a). Nitrate is abundant in vegetables (such as beetroot, spinach, and lettuce) and has been used as nutritional supplementation in healthy and diseased humans (Lidder and Webb, 2013; Ranchal-Sanchez et al., 2020; Babateen et al., 2021; Esen et al., 2022). We previously demonstrated in rats and humans that nitrate supplementation increases tissue nitrate/nitrite levels, that skeletal muscle contains a nitrate transporter (sialin) and nitrate/nitrite reductase [xanthine oxidoreductase (XOR)], and that skeletal muscle nitrate acts as a regulator to maintain a systemic NO homeostasis (Piknova et al., 2016a; Gilliard et al., 2018; Wylie et al., 2019; Piknova et al., 2022b; Kadach et al., 2022; Piknova et al., 2023). In a recent study, in rats administered with ^15^N-labeled nitrate, we found that nitrate treatment directly increases ^15^N-labeled nitrate and nitrite accumulation in skeletal muscle and upregulates skeletal muscle gene expression of nitrate transporters [*sialin* and *chloride channel 1* (*CLC1*)] and *XOR* (Park et al., 2023). Interestingly, ^15^N-labeled nitrate administration in humans also shows increased ^15^N-labeled nitrate accumulation in skeletal muscle and, following nitrate ingestion, a decline in ^15^N-labeled nitrate during exercise and the improvement of muscle torque production, indicating enhanced muscle contractile performance (Kadach et al., 2023).

However, skeletal muscle responses to nitrate supplementation in a model of nNOS deficiency and the role of the compensatory nitrate/nitrite reductive pathway are still unknown. In this study, we used genetically modified nNOS knockout (nNOS−/−) mice as a model lacking nNOS and losing catalytic activity for NO production (Huang et al., 1993) combined with the treatment of three nitrate supplementation conditions (i.e., high nitrate, low nitrate, and low nitrate followed by high nitrate) for comparison to wild-type (WT) mice. We hypothesized that nitrate supplementation results in high-nitrate levels in skeletal muscle cells due to transport changes from the media that allow reductive processes to compensate for the loss of nNOS in skeletal muscle.

## 2 Materials and methods

### 2.1 Animal and nitrate treatments

**FIGURE 1:**
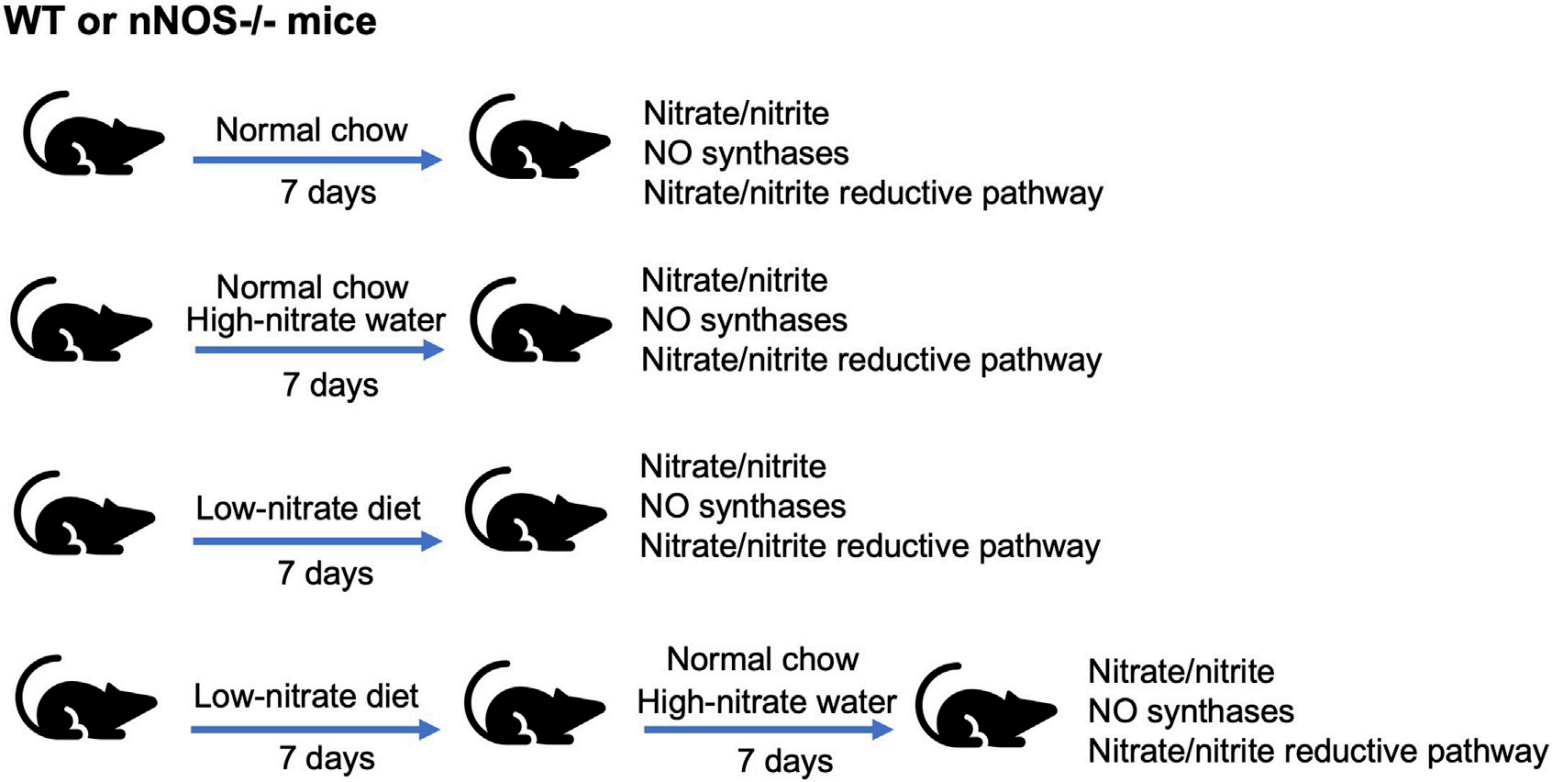
Nitrate intervention setup. WT and nNOS knockout (nNOS−/−) mice were treated with three nitrate interventions compared to normal chow control: high-nitrate water (1 g/L sodium nitrate) for 7 days, low-nitrate diet for 7 days, and low-nitrate diet for 7 days followed by high-nitrate water for 7 days. After treatments, tissue nitrate/nitrite levels and mRNA and protein expression related to NO synthases or nitrate/nitrite reductive pathway were investigated.

All animal procedures were approved by the NIDDK Animal Care and Use Committee (protocol: ASP-K048-MMB-22) and conducted according to recommendations in the Guide for the Care and Use of Laboratory Animals of the NIH. Adult 14–17-week-old wild-type (WT) and homozygous genetic nNOS knockout (nNOS−/−) (B6.129S4-*Nos1* ^*tm1Plh*^/J) C57BL/6 mice (Jackson Laboratory, United States) were used. Mice were acclimated at least 1 month after delivery and housed in a 12 h light/dark cycle environment with access to food and drinking water *ad libitum*. Both WT and nNOS−/− mice were divided into four groups of intervention (*n* = 11–16; mixed genders): control, high nitrate, low nitrate, and low nitrate followed by high nitrate (Figure 1). Control group mice were fed normal chow (standard mouse diet NIH-31, 386.9 nmol/g nitrate). High-nitrate group mice were fed with normal chow and drinking water containing 1 g/L sodium nitrate for 7 days. Low-nitrate group mice were fed with a low-nitrate diet (Harlan Teklad diet #TD99366, United States, 46.8 nmol/g nitrate) for 7 days. Mice in the low-nitrate followed by the high-nitrate group were fed with a low-nitrate diet for 7 days and switched to normal chow and drinking water containing 1 g/L sodium nitrate for an additional 7 days. The doses and regimens of nitrate supplementation were validated in previous publications (Park et al., 2013; Gilliard et al., 2018).

### 2.2 Sample collection

Mice were euthanized in an enclosed anesthesia box using a 5% isoflurane mixture with air. Anesthetized mice were taken off from the anesthesia box and placed on a pad in the supine position, and anesthesia was continued through a nose cone. The thoracic cavity was opened, and whole blood was collected by cardiac puncture using a heparin-coated syringe. The blood was immediately mixed with stop solution, a nitrite-preserving solution from degradation by hemoglobin, containing potassium ferricyanide (K_3_Fe(CN)_6_), N-ethylmaleimide (NEM), and Nonidet P-40 (NP-40) (400 μL blood: 100 μL stop solution) and flash frozen on dry ice for nitrate/nitrite assay (Kadach et al., 2022). Then, euthanized mice were perfused with saline containing heparin (heparin sodium, 2 USP units/mL) until there was no blood in the outgoing saline; the liver was discolored, and the heart was removed. Quadriceps skeletal muscle from the hind legs and the liver were collected quickly and immersed in RNAlater solution (Invitrogen, United States) for qPCR or flash frozen on dry ice for Western blot and nitrate/nitrite assay. All samples were kept at −80°C until analysis.

### 2.3 Nitrate/nitrite assay

Since tissue nitrate or nitrite accumulation represents an index of NO production, nitrate and nitrite concentrations in blood, quadriceps skeletal muscle, and liver samples were measured using a standard chemiluminescence method (Piknova et al., 2016b; Park et al., 2021a; Kadach et al., 2022). A volume of 500 μL of blood samples containing the stop solution as described above was mixed with methanol (1:2 ratio, sample: methanol, vol/vol), incubated on ice for 30 min to precipitate protein, and then centrifuged for 30 min (17,000 g at 4°C). Then, 100–150 mg of quadriceps skeletal muscle and liver samples were immersed in 500 μL of the diluted stop solution (1:9 ratio, stop solution: deionized water, vol/vol), homogenized using a bead homogenizer (Bertin Technologies, United States), mixed with methanol (1:2 ratio, sample: methanol, vol/vol), incubated on ice for 30 min for precipitating proteins, and then centrifuged for 30 min (17,000 g at 4°C). After centrifugation, the supernatant of all samples was collected and injected into the NO analyzer (NOA, Sievers, Model 280i, GE Analytical Instruments, United States) using helium as a carrier gas. Vanadium chloride or tri-iodide solution was used as the reducing agent for nitrate or nitrite analysis, respectively.

### 2.4 Quantitative real-time polymerase chain reaction

Quantitative real-time polymerase chain reaction (qPCR) was used to quantify mRNA expression of NO-related proteins in the NO synthases (e.g., *nNOS*, *eNOS*, and *iNOS*) and the nitrate/nitrite reductive pathway [nitrate/nitrite transporters (e.g., *sialin* and *chloride channels* (*CLC1* (expressed in skeletal muscle) and *CLC2* (expressed in the liver))) and nitrate/nitrite reductase (e.g., *xanthine oxidoreductase* (*XOR*))]. Quadriceps skeletal muscle and liver samples were collected and stored in RNAlater (Invitrogen, United States). RNA was extracted from 30–40 mg of tissues using a bead homogenizer (Bertin Technologies, United States) and the RNeasy Fibrous Tissue Mini Kit (QIAGEN, Germany). Then, 0.2–1 μg of RNA was reverse-transcribed to cDNA using a High-Capacity cDNA Reverse Transcription Kit (Applied Biosystems, United States). The qPCRs of cDNA samples were performed using PowerUp™ SYBR™ Green Master Mix (Applied Biosystems, United States) and run on the QuantStudio™ 7 Flex qPCR System (Applied Biosystems, United States), according to the manufacturer’s protocol. The primers used in qPCR are listed in Supplementary Table S1. The expression mRNA was normalized to housekeeping *ribosomal protein L13a* (*Rpl13a*) mRNA and is expressed as a relative fold change using the 2^−ΔΔCT^ method.

### 2.5 Western blot

Western blot was performed to analyze protein levels related to NO synthases [e.g., nNOS, eNOS, phospho-eNOS (Ser1177 and Thr495), and iNOS] and the nitrate/nitrite reductive pathway (e.g., sialin and XOR). Briefly, 100 mg of quadriceps skeletal muscle samples were homogenized in 1,000 μL of RIPA lysis buffer (Sigma, United States) containing protease inhibitors (Complete Tablets, Roche, Germany) and phosphatase inhibitors (PhosSTOP, Roche, Germany) using a bead homogenizer (Bertin Technologies, United States), incubated on ice for 30 min, and then centrifuged for 30 min (17,000 g at 4°C). The supernatant was collected to estimate protein concentrations using the BCA assay (Thermo Fisher Scientific, United States). Afterward, 50 μg of protein was run into 10% or 4%–12% SDS-PAGE (Invitrogen, United States) along with a protein marker or protein ladder (Precision Plus Protein™ Kaleidoscope™ Prestained Protein Standard, Bio-Rad, United States) and transferred to a nitrocellulose membrane (Invitrogen, United States). Membranes were blocked in 5% skim milk (5% BSA for phospho-eNOS antibodies) for 1 h at room temperature and incubated with a primary antibody in the blocking solution at 4°C overnight. The membranes were washed with the TBS-T buffer three times (5 min each) and then incubated with a second antibody, goat-anti-mouse antibody or goat-anti-rabbit antibody conjugated with horseradish peroxidase (Jackson ImmunoResearch, United States), in the blocking solution for 1 h at room temperature. After the TBS-T buffer mambrane wash (three times), protein bands were detected by enhanced chemiluminescence (ECL) (SuperSignal West Femto Maximum Sensitivity Substrate, Thermo Fisher Scientific, United States). Protein band and protein marker images were taken by 600-imager (Azure Biosystems, United States) using the chemiluminescence and color marker modes, respectively. The protein band image was merged with the protein marker image to compare the protein size (kDa). Band density was estimated using NIH ImageJ software and normalized to housekeeping glyceraldehyde-3-phosphate dehydrogenase (GAPDH). Antibodies used for Western blot analyses are listed in Supplementary Table S2.

### 2.6 Statistical analysis

All data are presented as the mean, standard deviation, median, and interquartile range (IQR). Outliers were not excluded because they looked like they represented real responses to the diets. Two-factor analyses of variance with Tukey’s adjustment for multiple comparisons were performed to compare the responses of each diet to the control diet and the responses of WT and nNOS−/− mice. In some cases, unpaired *t*-tests were used because of the high variability of results. Statistical analysis was performed using GraphPad Prism^®^ software and SAS^®^ version 9.4. The number of observations, mean, standard deviation, median, and additional statistical parameters are included for each figure in Supplementary Table S3–S15.

## 3 Results

### 3.1 In nNOS−/− mice, the loss of nNOS decreases skeletal muscle nitrate levels

**FIGURE 2:**
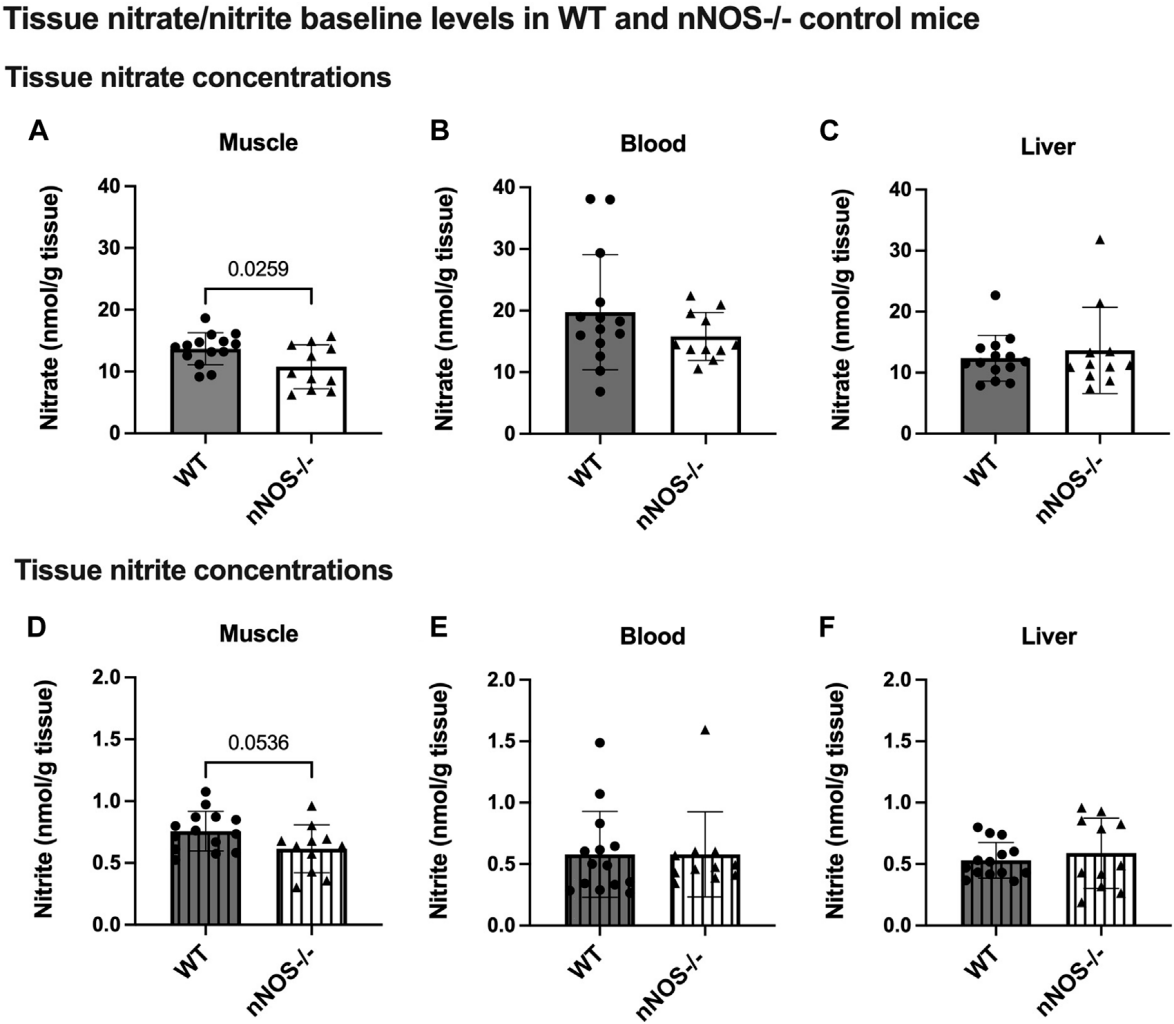
Tissue nitrate/nitrite baseline levels in WT and nNOS−/− control mice. Tissue nitrate **(A–C)** and nitrite **(D–F)** concentrations were measured from the skeletal muscle, blood, and liver of WT control mice (*n* = 14; 7 male mice and 7 female mice) and nNOS−/− control mice (*n* = 11; 6 male mice and 5 female mice). Data are represented as the mean ± SD. The statistics was tested using the unpaired *t*-test.

In WT mice, nNOS gene expression and proteins were readily detected in skeletal muscle but not in nNOS−/− mice (Supplementary Figure S1). Baseline levels of tissue nitrate and nitrite concentrations, representing the index of NO production, were measured in the skeletal muscle, blood, and liver of WT and nNOS−/− mice (Figure 2). Mice that lack nNOS expression (nNOS−/−) showed significantly lower nitrate baseline levels in skeletal muscle (Figure 2A) than WT mice with a −2.91 nmol/g tissue mean difference (Supplementary Table S3). There was also a decreasing trend for nitrite levels in the skeletal muscle of nNOS−/− mice (*p* = 0.0536) (Figure 2D) compared with WT mice with a −0.14 nmol/g tissue mean difference (Supplementary Table S3). These data suggest that NO production is lower in the skeletal muscle of nNOS−/− mice than in WT mice.

### 3.2 High-nitrate supplementation results in a greater increase in nitrate accumulation in nNOS−/− mice

**FIGURE 3:**
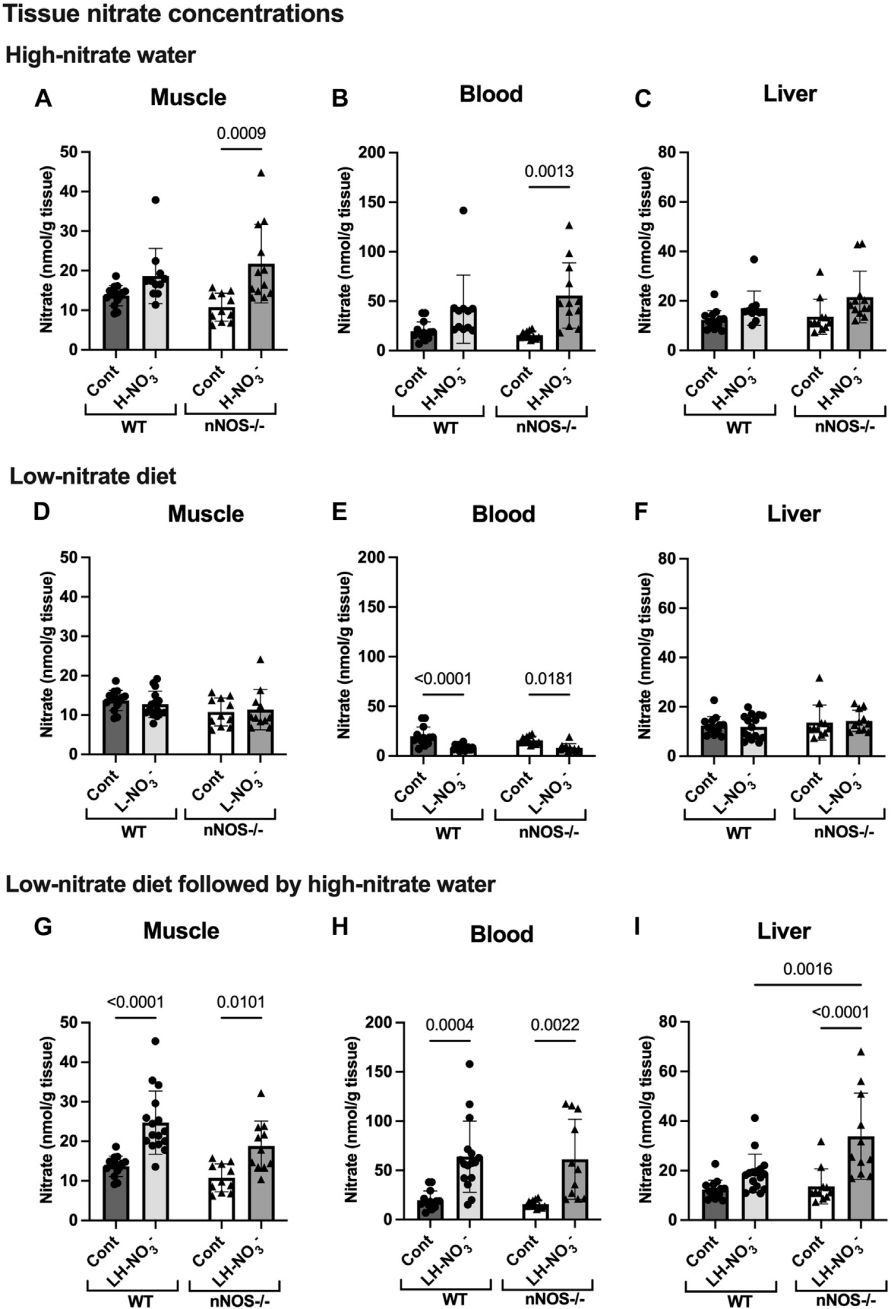
Tissue nitrate concentrations. Tissue nitrate concentrations in the skeletal muscle, blood, and liver of WT and nNOS−/− mice were examined after the mice were treated with three nitrate interventions compared to normal chow control (Cont) [WT: *n* = 14 (7 male mice and 7 female mice); nNOS−/−: *n* = 11 (6 male mice and 5 female mice)]: high-nitrate water (1 g/L sodium nitrate for 7 days, H-NO_3_^-^) [WT: n = 11 (6 male mice and 5 female mice); nNOS−/−: *n* = 12 (7 male mice and 5 female mice)] **(A–C)**, a low-nitrate diet for 7 days (L-NO_3_^-^) [WT: *n* = 15 (8 male mice and 7 female mice); nNOS−/−: *n* = 11 (6 male mice and 5 female mice)] **(D–F)**, and a low-nitrate diet for 7 days followed by high-nitrate water for 7 days (LH-NO_3_^-^) [WT: *n* = 16 (8 male mice and 8 female mice); nNOS−/−: *n* = 11 (6 male mice and 5 female mice)] **(G–I)**. Data are presented as the mean ± SD. The statistics was tested using a two-way ANOVA with Tukey’s adjustment for multiple comparisons. Note that the values in the WT and nNOS−/− control groups were identical to the values in Figure 2 and used to compare all treatment groups.

**FIGURE 4:**
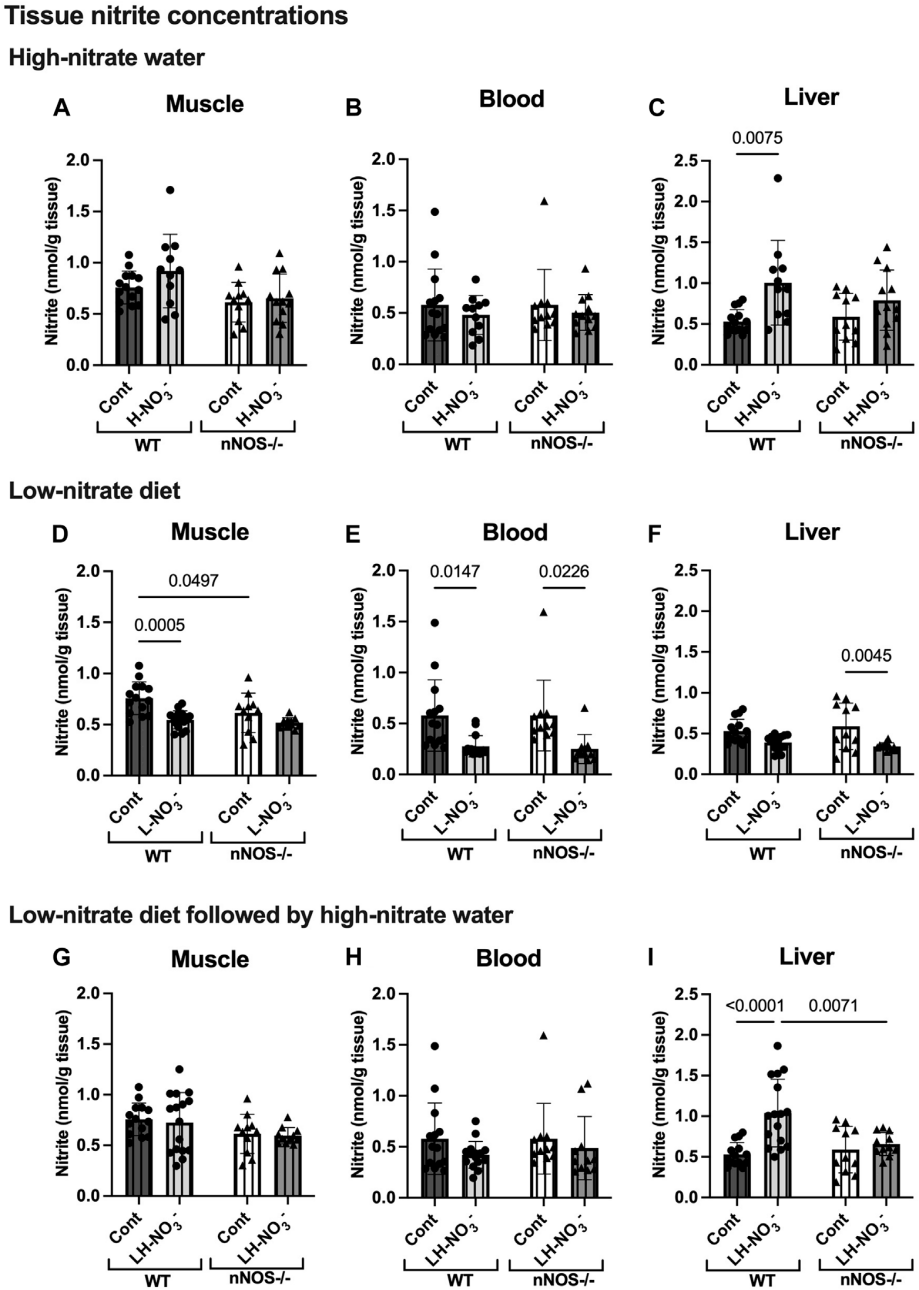
Tissue nitrite concentrations. Tissue nitrite concentrations in the skeletal muscle, blood, and liver of WT and nNOS−/− mice were examined after the mice were treated with three nitrate interventions compared to normal chow control (Cont) [WT: *n* = 14 (7 male mice and 7 female mice); nNOS−/−: *n* = 11 (6 male mice and 5 female mice)]: high-nitrate water (1 g/L sodium nitrate for 7 days, H-NO_3_^-^) [WT: *n* = 11 (6 male mice and 5 female mice); nNOS−/−: *n* = 12 (7 male mice and 5 female mice)] **(A–C)**, a low-nitrate diet for 7 days (L-NO_3_^-^) [WT: *n* = 15 (8 male mice and 7 female mice); nNOS−/−: *n* = 11 (6 male mice and 5 female mice)] **(D–F)**, and a low-nitrate diet for 7 days followed by high-nitrate water for 7 days (LH-NO_3_^-^) [WT: *n* = 16 (8 male mice and 8 female mice); nNOS−/−: *n* = 11 (6 male mice and 5 female mice)] **(G–I)**. Data are presented as the mean ± SD. The statistics was tested using a two-way ANOVA with Tukey’s adjustment for multiple comparisons. Note that the values in the WT and nNOS−/− control groups were identical to the values in Figure 2 and used to compare all treatment groups.

Supplementation with high-nitrate water (H-NO_3_^-^) significantly increased the nitrate levels in nNOS−/− mice in skeletal muscle (Figure 3A) and blood (Figure 3B) compared to normal chow control (Cont). Compared to their control, H-NO_3_^-^-treated nNOS−/− mice showed a greater nitrate accumulation in skeletal muscle, blood, and liver (10.99, 39.91, and 7.93 nmol/g tissue mean difference, respectively) than H-NO_3_^-^-treated WT mice (4.96, 22.15, and 4.73 nmol/g tissue mean difference, respectively) (Supplementary Table S4). However, WT mice on high-nitrate water showed increased nitrite concentrations in the liver (Figure 4C) but not nNOS−/− mice. Although nNOS−/− mice showed a greater level of tissue nitrate with high-nitrate supplementation, H-NO_3_^-^-treated WT mice showed significant nitrate accumulation in skeletal muscle, blood, and liver when using the unpaired *t*-test (Supplementary Figure S2).

### 3.3 Low-nitrate diet reduces nitrate/nitrite accumulation in WT and nNOS−/− mice

To investigate NO production in the nitrate depletion condition, mice were placed on a low-nitrate diet (L-NO_3_^-^). In both WT and nNOS−/− mice, L-NO_3_^-^ treatment significantly lowered nitrate levels in the blood (Figure 3E) but not in skeletal muscle (Figure 3D) and liver (Figure 3F). Moreover, this treatment decreased nitrite levels in the skeletal muscle and blood of WT and the blood and liver of nNOS−/− mice (Figures 4D–F). In the low-nitrate diet dataset, nNOS deficiency only caused significant loss of nitrite in muscle (*p* = 0.0497) (Figure 4D) but not in other compartments (Figures 4E, F), while nitrate levels were not different in nNOS−/− mice compared to WT mice (Figures 3D–F). The decrease in nitrite is more likely caused by the loss of nNOS’s NO-producing capacity. These results indicate that nitrate/nitrite accumulation is decreased in nitrate starvation. Interestingly, in skeletal muscle, L-NO_3_^-^-treated nNOS−/− mice did not show a decrease in nitrate with a 0.58 nmol/g tissue mean difference, whereas L-NO_3_^-^-treated WT mice revealed a reduction in nitrate with a −0.98 nmol/g tissue mean difference when compared to their control (Supplementary Table S4).

### 3.4 Low nitrate followed by high-nitrate supplementation increases nitrate/nitrite accumulation in WT and maintains high-nitrate levels in nNOS−/− mice

To mimic conditions of low-nitrate production in nNOS−/− mice, WT mice were pretreated with a low-nitrate diet for 1 week prior to 1 week of high-nitrate water treatment on normal chow (LH-NO_3_^-^). Similar to H-NO_3_^-^ treatment in nNOS−/− mice, LH-NO_3_^-^ treatment in nNOS−/− mice also showed a significant increase in tissue nitrate concentrations in skeletal muscle (Figure 3G), blood (Figure 3H), and liver (Figure 3I). Interestingly, LH-NO_3_^-^ treatment in WT mice showed a significantly greater increase in tissue nitrate concentrations in skeletal muscle (Figure 3G) and blood (Figure 3H), with 11.03 and 44.15 nmol/g tissue mean differences, respectively, compared to WT control mice (Supplementary Table S4). The quantification of nitrite levels showed increased nitrite concentrations in the liver of LH-NO_3_^-^-treated WT mice compared to WT control and LH-NO_3_^-^-treated nNOS−/− mice (Figure 4I). These results demonstrate that low-nitrate pretreatment followed by high-nitrate supplementation can enhance nitrate/nitrite accumulation in WT mice with the high-nitrate loading observed in nNOS−/− mice supplemented with high nitrate.

### 3.5 Greater nitrate accumulation in the skeletal muscle of high-nitrate-treated nNOS−/− mice is induced via the nitrate/nitrite reductive pathway

**FIGURE 5:**
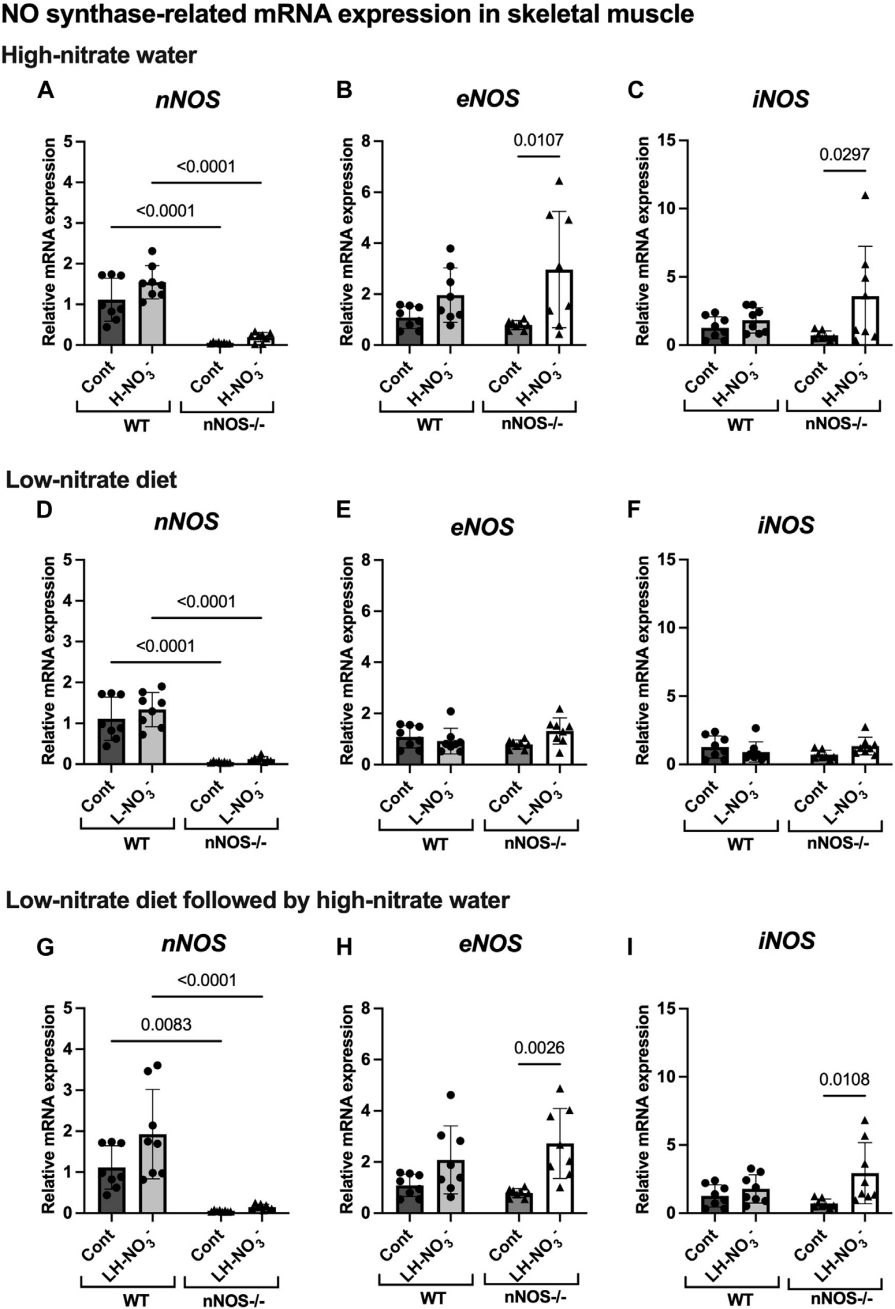
NO synthase-related mRNA expression in skeletal muscle. The mRNA expression of proteins related to NO synthases (*nNOS*, *eNOS*, and *iNOS*) in quadriceps skeletal muscle was detected using qPCR after WT and nNOS−/− mice (*n* = 8; 4 male mice and 4 female mice) were treated with three nitrate interventions compared to normal chow control (Cont): high-nitrate water (1 g/L sodium nitrate for 7 days, H-NO_3_^-^) **(A–C)**, a low-nitrate diet for 7 days (L-NO_3_^-^) **(D–F)**, and a low-nitrate diet for 7 days followed by high-nitrate water for 7 days (LH-NO_3_^-^) **(G–I)**. Relative mRNA expression was acquired by normalizing to *Rpl13a* and is presented as a fold change (mean ± SD) compared to the WT control group. The statistics was tested using a two-way ANOVA with Tukey’s adjustment for multiple comparisons. Note that all treatment groups were compared to the identical values of the WT and nNOS−/− control groups.

**FIGURE 6:**
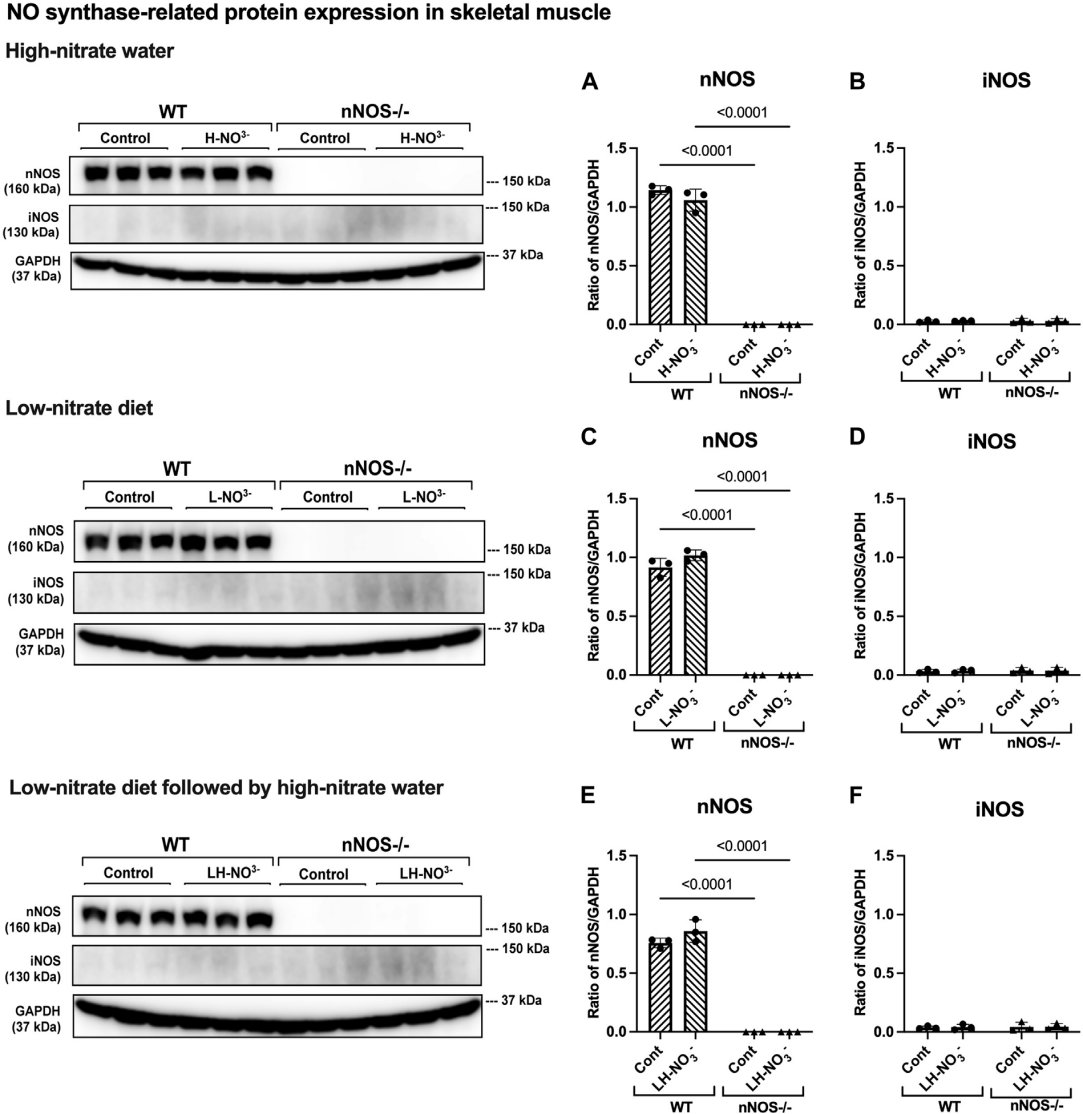
nNOS and iNOS protein expression in skeletal muscle. NO synthase-related protein expression (nNOS and iNOS) in quadriceps skeletal muscle was detected using Western blot analysis after WT and nNOS−/− mice (*n* = 3; 2 male mice and 1 female mouse) were treated with three nitrate interventions compared to normal chow control (Cont): high-nitrate water (1 g/L sodium nitrate for 7 days, H-NO_3_^-^) **(A, B)**, a low-nitrate diet for 7 days (L-NO_3_^-^) **(C, D)**, and a low-nitrate diet for 7 days followed by high-nitrate water for 7 days (LH-NO_3_^-^) **(E, F)**. Protein expression is presented as a ratio of band density normalized to GAPDH (mean ± SD). The statistics was tested using a two-way ANOVA with Tukey’s adjustment for multiple comparisons or unpaired *t*-test*.

**FIGURE 7:**
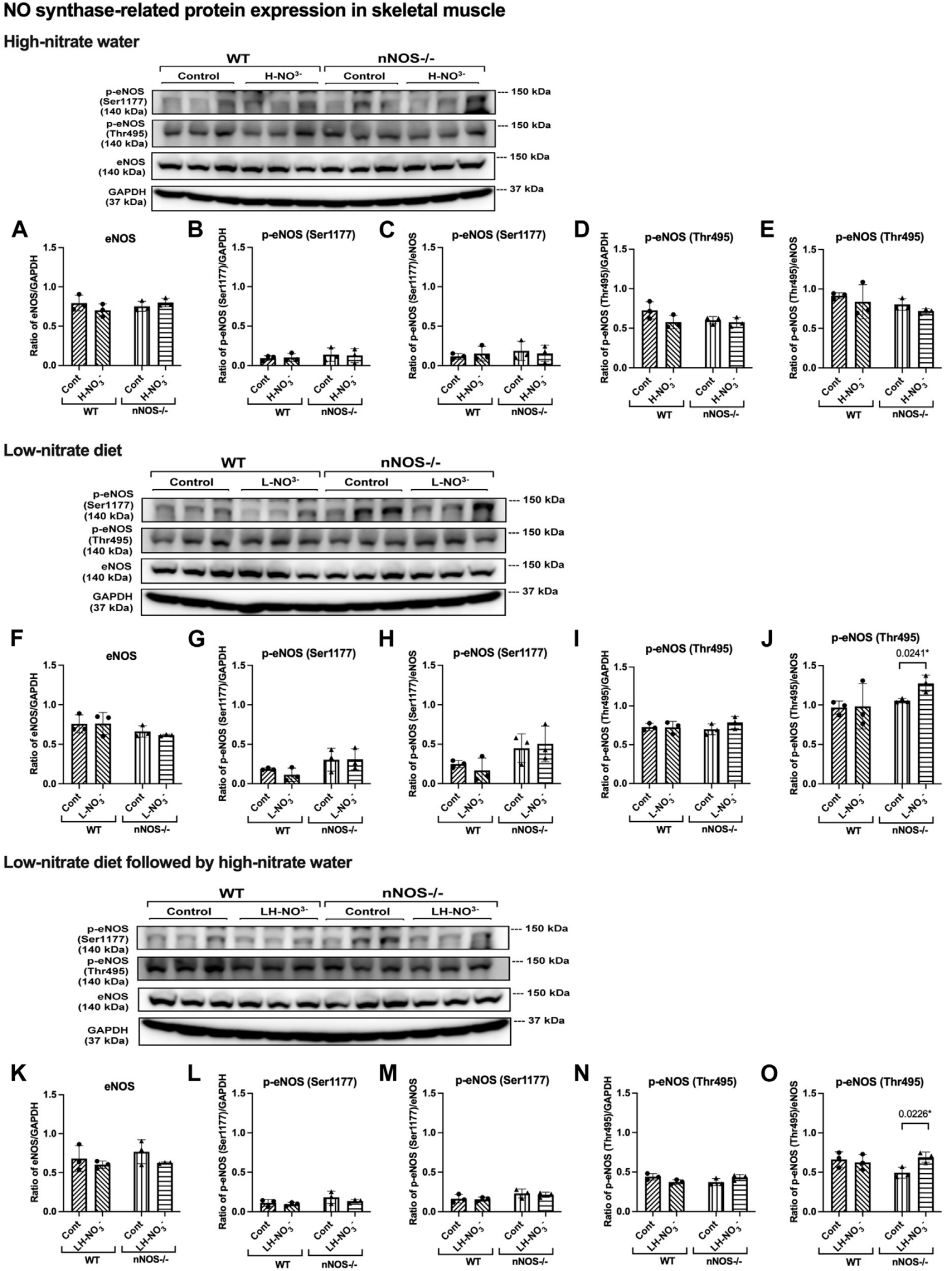
eNOS and phosphorylated eNOS protein expression in skeletal muscle. NO synthase-related protein expression [eNOS and phosphorylated eNOS related to eNOS activation activity (p-eNOS (Ser1177)) and eNOS inhibitory activity (p-eNOS (Thr495))] in quadriceps skeletal muscle was detected using Western blot analysis after WT and nNOS−/− mice (*n* = 3; 2 male mice and 1 female mouse) were treated with three nitrate interventions compared to normal chow control (Cont): high-nitrate water (1 g/L sodium nitrate for 7 days, H-NO_3_^-^) **(A–E)**, a low-nitrate diet for 7 days (L-NO_3_^-^) **(F–J),** and a low-nitrate diet for 7 days followed by high-nitrate water for 7 days (LH-NO_3_^-^) **(K–O)**. Protein expression is presented as a ratio of band density normalized to GAPDH or eNOS (mean ± SD). The statistics was tested using a two-way ANOVA with Tukey’s adjustment for multiple comparisons or unpaired *t*-test*.

To determine whether high-nitrate levels in skeletal muscle are involved via either the NO synthases or alternate nitrate/nitrite reductive pathway, mRNA and protein expression related to the NO synthases (nNOS, eNOS, and iNOS) and nitrate/nitrite reductive pathway (sialin, CLC1, and XOR) were assessed. None of the treatments affected *nNOS*, *eNOS*, or *iNOS* mRNA expression or protein in WT mice, with iNOS protein below the level of detection (Figures 5– 7). Consistent with the low levels of iNOS protein, little or no changes were observed in *IL6* mRNA expression in skeletal muscle in WT or nNOS−/− mice with or without treatments (Supplementary Figure S3), suggesting no significant inflammatory response. Little or no *nNOS* mRNA expression or protein was detected in nNOS−/− mice, and *eNOS* and *iNOS* mRNA and protein levels were comparable to those of WT mice (Figures 5– 7). Interestingly, the loss of nNOS in skeletal muscle did not result in increased *eNOS* or *iNOS* mRNA and protein expression in nNOS−/− mice. These results suggest that the contribution from NO synthases to NO production in WT mice and nNOS−/− mice is similar.

**FIGURE 8:**
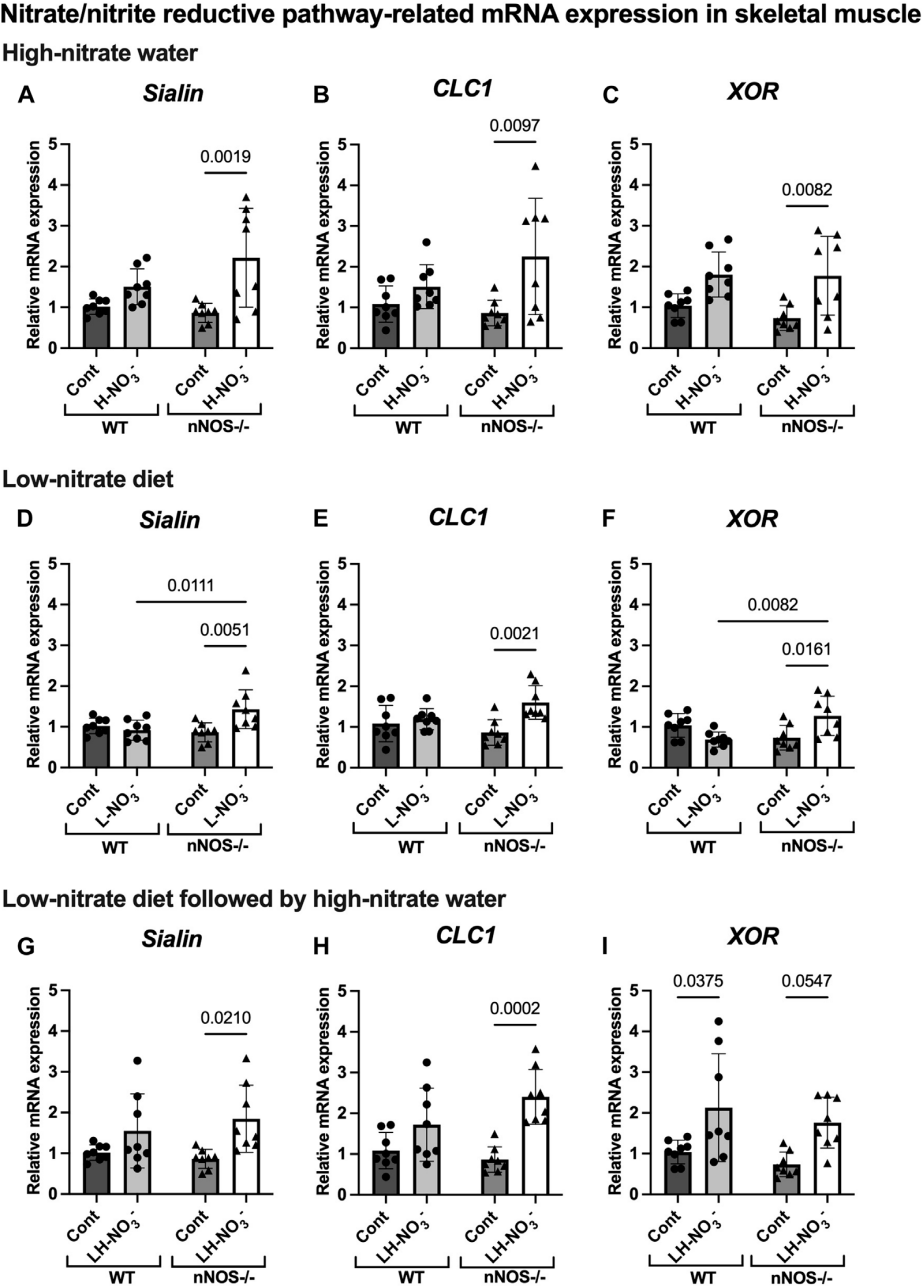
Nitrate/nitrite reductive pathway-related mRNA expression in skeletal muscle. The mRNA expression of proteins related to the nitrate/nitrite reductive pathway [nitrate transporters (*sialin* and *CLC1*) and nitrate/nitrite reductase (*XOR*)] in quadriceps skeletal muscle was detected using qPCR after WT and nNOS−/− mice (*n* = 8; 4 male mice and 4 female mice) were treated with three nitrate interventions compared to normal chow control (Cont): high-nitrate water (1 g/L sodium nitrate for 7 days, H-NO_3_^-^) **(A–C)**, a low-nitrate diet for 7 days (L-NO_3_^-^) **(D–F)**, and a low-nitrate diet for 7 days followed by high-nitrate water for 7 days (LH-NO_3_^-^) **(G–I)**. Relative mRNA expression was acquired by normalizing to *Rpl13*a and is presented as a fold change (mean ± SD) compared to the WT control group. The statistics was tested using a two-way ANOVA with Tukey’s adjustment for multiple comparisons. Note that all treatment groups were compared to the identical values of WT and nNOS−/− control groups.

**FIGURE 9:**
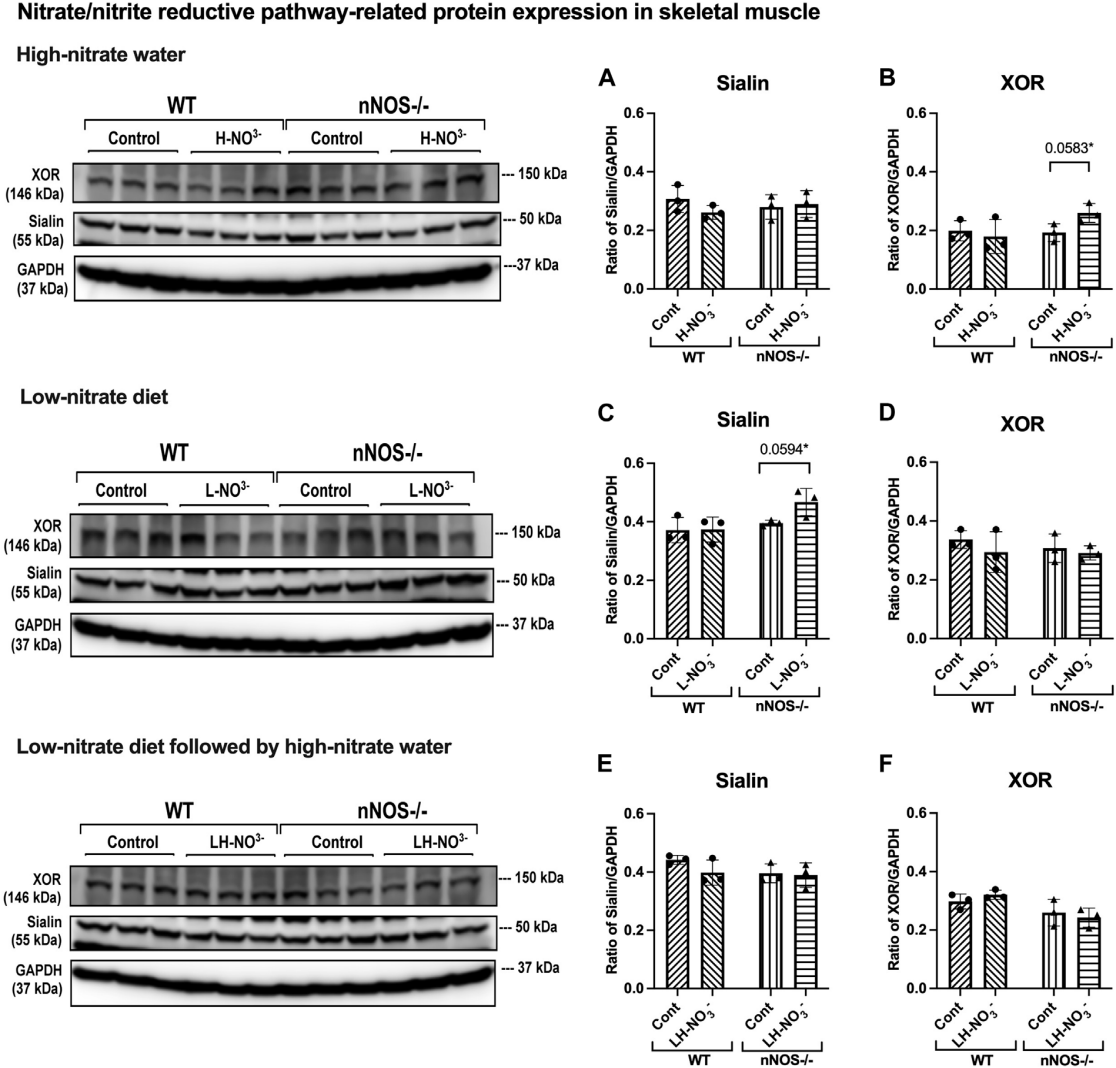
Nitrate/nitrite reductive pathway-related protein expression in skeletal muscle. Protein expression of proteins related to the nitrate/nitrite reductive pathway (sialin and XOR) in quadriceps skeletal muscle was detected using Western blot analysis after WT and nNOS−/− mice (*n* = 3; 2 male mice and 1 female mouse) were treated with three nitrate interventions compared to normal chow control (Cont): high-nitrate water (1 g/L sodium nitrate for 7 days, H-NO_3_^-^) **(A, B)**, a low-nitrate diet for 7 days (L-NO_3_^-^) **(C, D)**, and low-nitrate diet for 7 days followed by high-nitrate water for 7 days (LH-NO_3_^-^) **(E, F)**. Protein expression is presented as a ratio of band density normalized to GAPDH (mean ± SD). The statistics was tested using a two-way ANOVA with Tukey’s adjustment for multiple comparisons or unpaired *t*-test*.

In H-NO_3_^-^-treated nNOS−/− mice, *eNOS* and *iNOS* mRNA expression appeared to be significantly increased (Figures 5B, C), and eNOS protein was comparable to nNOS−/− control mice, including eNOS activities in both activation [p-eNOS (Ser1177)] and inhibition [p-eNOS (Thr495)] (Figures 6B, 7A–E). Skeletal muscle iNOS protein remained below the level of detection. However, the expressions of the nitrate/nitrite reductive pathway-associated genes, *sialin*, *CLC1*, and *XOR*, were significantly increased in H-NO_3_^-^-treated nNOS−/− mice compared to nNOS−/− control mice (Figures 8A–C), and there was a trend to increase XOR protein compared to nNOS−/− control mice (*p* = 0.0583, unpaired *t*-test) (Figure 9B). These data indicate that nitrate overload or greater nitrate/nitrite accumulation in high-nitrate supplementation is generated by the skeletal muscle of nNOS−/− mice via the nitrate/nitrite reductive pathway with the loss of nNOS. In contrast to skeletal muscle, liver mRNA expressions of *eNOS*, *sialin*, *CLC2*, and *XOR* were not significantly changed with the H-NO_3_^-^ treatment condition (Supplementary Figures S4A, S5A–C), and liver *nNOS* and *iNOS* mRNA expression were undetermined by qPCR.

### 3.6 Lower nitrate/nitrite accumulation in low-nitrate-treated nNOS−/− mice is regulated by skeletal muscle via the nitrate/nitrite reductive pathway

Surprisingly, mRNA expression of NO-related proteins involved in the nitrate/nitrite reductive pathway was significantly upregulated only in the skeletal muscle of L-NO_3_^-^-treated nNOS−/− mice (Figures 8D–F), while the reduction in nitrate/nitrite accumulation was notable in blood (Figures 3E, 4E) but not in skeletal muscle (Figures 3D, 4D). We found that *sialin*, *CLC1*, and *XOR* mRNA expressions in the skeletal muscle of this group were greater than in the nNOS−/− control or L-NO_3_^-^-treated WT group (Figures 8D–F), along with a trend of increase in sialin protein (*p* = 0.0594, unpaired *t*-test) (Figures 9C,D) and eNOS inhibitory activity [p-eNOS (Thr495)] levels (*p* = 0.0241, unpaired *t*-test) (Figure 7J). L-NO_3_^-^ treatment did not significantly alter *nNOS*, *eNOS*, and *iNOS* mRNA expression (Figures 5D–F) or protein levels (Figures 6C, D and Figures 7F–J) in the skeletal muscle of WT or nNOS−/− mice. In the liver, the expression of *eNOS* or nitrate/nitrite reductive pathway-associated genes was not significantly changed between control and L-NO_3_^-^ treatment (Supplementary Figures S4B, S5D–F). Thus, these findings demonstrate that the skeletal muscle of nNOS−/− mice seems to regulate lower nitrate/nitrite accumulation in nitrate depletion via the nitrate/nitrite reductive pathway.

### 3.7 Low nitrate followed by high-nitrate treatment can enhance nitrate/nitrite accumulation in WT mice and maintain high-nitrate levels in nNOS−/− mice via the nitrate/nitrite reductive pathway

Since we found that low nitrate followed by high-nitrate supplementation could enhance nitrate/nitrite accumulation in WT mice (Figures 3G, H, 4I), we previously believed that this condition would mimic the nitrate overloading in nNOS−/− mice treated with high nitrate (Figures 3A, B). Unlike H-NO_3_^-^-treated nNOS−/− mice, the skeletal muscle of LH-NO_3_^-^-treated WT mice showed a significant increase only in *XOR* mRNA expression (Figures 5G–I, 8G–I), whereas the skeletal muscle of H-NO_3_^-^-treated nNOS−/− mice exhibited significantly increased mRNA expression of *eNOS*, *iNOS*, *sialin*, *CLC1*, and *XOR* (Figures 5A–C, 8A–C). In LH-NO_3_^-^-treated WT mice, no significant changes in proteins associated with the nitrate/nitrite reductive pathway were detected in skeletal muscle (Figures 9E, F), while nNOS−/− mice treated with H-NO_3_^-^ showed an increasing trend of XOR protein in skeletal muscle (*p* = 0.0583, unpaired *t*-test) (Figures 9A, B). In LH-NO_3_^-^-treated WT mice and H-NO_3_^-^-treated nNOS−/− mice, no significant changes in the skeletal muscle of nNOS or eNOS protein or in eNOS activities, p-eNOS (Ser1177) and p-eNOS (Thr495), were observed, and iNOS protein remained below the level of detection (Figures 6A, B, E, F and Figures 7A–E, K–O). Although LH-NO_3_^-^-treated WT mice cannot mimic H-NO_3_^-^-treated nNOS−/− mice, these results suggest that the nitrate/nitrite reductive pathway has more impact on enhancing nitrate/nitrite accumulation in skeletal muscle than NO synthases in LH-NO_3_^-^-treated WT mice and H-NO_3_^-^-treated nNOS−/− mice.

Like H-NO_3_^-^-treated nNOS−/− mice, LH-NO_3_^-^-treated nNOS−/− mice showed consistent and significant mRNA upregulation in the skeletal muscle of *eNOS*, *iNOS*, *sialin*, and *CLC1*, with an increasing trend in *XOR* (*p* = 0.0547) (Figures 5H, I and Figures 8G–I) but not in the liver (Supplementary Figures S4C and S5G–I), and protein levels were comparable to levels in nNOS−/− control mice (Figures 6F, 7K–N, 9E,F). A trend of increased inhibitory activity of eNOS [p-eNOS (Thr495)] was also observed in LH-NO_3_^-^-treated nNOS−/− mice compared to nNOS−/− control mice (*p* = 0.0226, unpaired *t*-test) (Figure 7O). These results indicate that the high-nitrate levels in LH-NO_3_^-^-treated nNOS−/− mice are involved and maintained using the nitrate/nitrite reductive pathway but not by NO synthases.

## 4 Discussion

Our results demonstrate that three nitrate treatment conditions show compensatory increases involving the nitrate/nitrite reductive pathway for NO production in the skeletal muscle of nNOS knockout mice that lack the nNOS-dependent pathway. First, high-nitrate water supplementation in nNOS−/− mice exhibits greater tissue nitrate/nitrite accumulation via the nitrate/nitrite reductive pathway. Second, on low-nitrate diet feeding, lower nitrate/nitrite accumulation in nNOS−/− mice is concomitant with increased expression of the nitrate/nitrite reductive pathway. Finally, low-nitrate diet pretreatment followed by high-nitrate water supplementation can enhance nitrate/nitrite accumulation in WT mice and, as with high-nitrate water supplementation alone, maintain high-nitrate levels in nNOS−/− mice via the nitrate/nitrite reductive pathway.

Since nNOS−/− mice used in this study are generated by placing the neomycin resistance gene in exon 1 of the *nNOS (NOS1)* gene encoding the initiation site and amino acids 1–159 of the protein, nNOS−/− mice show loss of nNOS and catalytic activity to produce NO (Huang et al., 1993). Since NO has a short half-life, it is difficult to quantify NO directly. Nitrate and nitrite are the stable oxidized products of NO. There are two sources of nitrate and nitrite: NO synthases and diet. NO generated by NO synthases is oxidized to nitrate by oxyhemoglobin and to nitrite by ceruloplasmin (Lundberg et al., 2008). Therefore, the accumulation of nitrate or nitrite is preferably measured as an index of NO production (Giustarini et al., 2008). Although nitrate and nitrite levels were not different at the baseline before supplementation in WT and nNOS−/− mice using two-way ANOVA with Tukey’s multiple comparisons (Figures 3, 4), our results in the comparison to WT mice using the unpaired *t*-test suggest that the loss of nNOS resulted in a decrease in the baseline nitrate levels in skeletal muscle (Figure 2A), representing downregulation of NO production in the skeletal muscle of nNOS−/− mice. Interestingly, the high-nitrate supplementation revealed a greater effect in nNOS−/− mice than in WT mice. The results showed high-nitrate/nitrate accumulation in the skeletal muscle and blood of H-NO_3_^-^-treated nNOS−/− mice. Skeletal muscle mRNA expressions of *eNOS* and *iNOS* and proteins associated with the nitrate/nitrite reductive pathway (*sialin*, *CLC1*, and *XOR*) were significantly increased in H-NO_3_^-^-treated nNOS−/− mice but not in H-NO_3_^-^-treated WT mice. Since we showed robust skeletal muscle expression of NOS proteins, especially nNOS and eNOS compared with iNOS in WT mice, it is possible that other NO synthases can drive NO production in skeletal muscle lacking nNOS. The increased *iNOS* mRNA expression in high-nitrate-treated nNOS−/− mice was not related to *IL6* mRNA expression, indicating that there was no effect of inflammation with iNOS activation. The activity of eNOS is regulated by phosphorylated post-translational modification, namely, phospho-eNOS (p-eNOS): p-eNOS (Ser1177) levels represent the activated form of eNOS, while p-eNOS (Thr495) levels are associated with inactivated eNOS (Michell et al., 2001). Although nNOS protein is absent in nNOS−/− mice, eNOS and iNOS proteins, as well as eNOS activities [p-eNOS (Ser1177) and p-eNOS (Thr495)], were not significantly increased compared to control mice, and the levels of proteins associated with the nitrate/nitrite reductive pathway were similar to control mice with some increasing trend in XOR (*p* = 0.0583, unpaired *t*-test). The crosstalk between the nitrate/nitrite reductive pathway and NO synthases in nitrate treatment is further supported by the previous finding in rats that long-term dietary nitrate treatment results in increased plasma nitrate but downregulation of vascular eNOS activity with a reduction in p-eNOS (Ser1177) and an increase in p-eNOS (Thr495) in the aorta (Carlstrom et al., 2015). Altogether, in the nNOS−/− mice that lack nNOS, high-nitrate water did not alter the expression of other NOS isoforms (i.e., eNOS and iNOS), suggesting that NO synthases do not participate in or are not the primary contributors to nitrate/nitrite metabolism during nitrate supplementation. The increased nitrate/nitrite levels following treatment with high-nitrate water in nNOS−/− mice suggest a greater contribution to NO production via the nitrate/nitrite reductive pathway in NOS−/− mice.

In this study, however, we did not observe a significant increase in tissue nitrate/nitrite concentrations or *sialin*, *CLC1*, and *XOR* mRNA expression in WT mice treated with high-nitrate water, although our previous studies in rats and humans revealed higher tissue nitrate/nitrite levels and skeletal muscle *sialin*, *CLC1*, and *XOR* mRNA expression in this condition (Gilliard et al., 2018; Wylie et al., 2019; Park et al., 2023). These results suggest that the mice may be less responsive to nitrate supplementation compared to rats and humans. However, when using unpaired *t*-test statistical analysis and considering only WT mice, as in a previous publication in humans and rats (Gilliard et al., 2018; Wylie et al., 2019), H-NO_3_^-^-treated WT mice showed significantly higher nitrate levels in skeletal muscle, blood, and liver than WT control mice (Supplementary Figure S2). Therefore, these results clarify that high-nitrate supplementation can increase nitrate accumulation in WT mice. Furthermore, in nNOS knockout mice exhibiting lower skeletal muscle nitrate levels, high-nitrate supplementation can further increase nitrate accumulation in skeletal muscle, with a greater contribution from the nitrate/nitrite reductive pathway due to the decreased activation of the NO synthases with the loss of nNOS. Consequently, these findings can account for why high-nitrate supplementation appeared more effective in increasing nitrate accumulation in nNOS−/− mice than in WT mice.

We previously found in a rat model that a low-nitrate diet can drop tissue nitrate/nitrite levels (Gilliard et al., 2018). We proposed to mimic nNOS−/− mice by feeding WT mice with a low-nitrate diet. nNOS−/− mice have lower nitrate baseline levels in skeletal muscle, while WT mice fed with a low-nitrate diet have lower levels of nitrate in blood and nitrite in skeletal muscle and blood. Although L-NO_3_^-^-treated WT mice cannot mimic nNOS−/−mice, these results demonstrated that a low-nitrate diet can reduce tissue nitrate/nitrite accumulation in WT mice. In L-NO_3_^-^-treated nNOS−/− mice, we also found lower nitrate and nitrite levels in the blood but not in skeletal muscle. In contrast, only L-NO_3_^-^-treated nNOS−/− mice had mRNA upregulation of *sialin*, *CLC1*, and *XOR*, along with an increasing trend of sialin protein (*p* = 0.0594, unpaired *t*-test) in skeletal muscle related to the nitrate/nitrite reductive pathway. These responses may result in a little or undetectable decrease in nitrate/nitrite levels in the skeletal muscle of the L-NO_3_^-^-treated mice when compared to mice on normal chow, or nNOS−/−mice might maintain nitrate/nitrate accumulation in skeletal muscle in nitrate starvation or depletion conditions via the nitrate/nitrite reductive pathway. Interestingly, in rats, we observed that high-nitrate treatment followed by a return to normal chow is accompanied by decreased nitrate and nitrite levels and the upregulation of sialin and XOR proteins (Park et al., 2021b). Hence, nNOS−/− mice, but not WT mice, show regulation of lower NO production in skeletal muscle in nitrate depletion via increased expression of the nitrate/nitrite reductive pathway.

In nNOS−/− mice, high nitrate could give rise to greater nitrate accumulation, providing evidence for the activation of the nitrate/nitrite reductive pathway. This is consistent with a previous study in rats that showed greater levels of nitrate accumulation in skeletal muscle after high-nitrate supplementation when pretreated with a low-nitrate diet prior to nitrate supplementation (Gilliard et al., 2018). Furthermore, WT mice on LH-NO_3_^-^ treatment (pretreatment with a low-nitrate diet followed by H-NO_3_^-^ treatment), set up to mimic in part the condition of H-NO_3_^-^-treated nNOS−/− mice, showed significantly greater nitrate/nitrite accumulation than H-NO_3_^-^ treatment alone, in agreement with previous observations in rats (Gilliard et al., 2018). Since L-NO_3_^-^-treated WT mice did not mimic nNOS−/− mice as described above, LH-NO_3_^-^-treated WT mice cannot mimic H-NO_3_^-^-treated nNOS−/− mice as well. Unlike WT mice, both combined LH-NO_3_^-^ treatment and H-NO_3_^-^ treatment in nNOS−/− mice resulted in greater nitrate accumulation than in control mice. Like high-nitrate and low-nitrate treatments, we found that the nitrate/nitrite reductive pathway contributes to the greater nitrate accumulation in this combined LH-NO_3_^-^ treatment in nNOS−/− mice, but LH-NO_3_^-^-treated WT mice showed only an increase in *XOR* mRNA expression. Interestingly, WT mice with low-nitrate diet pretreatment followed by high-nitrate water showed a greater increase in tissue nitrate accumulation as seen in nNOS−/− mice-treated high-nitrate water. This suggests that tissue nitrate/nitrate depletion may mediate a greater increase in tissue nitrate/nitrite accumulation in nNOS−/− mice.

Increased expression of nitrate/nitrite reductive pathway-associated genes was observed in nNOS−/− mice treated with the low-nitrate diet that did not show a lower nitrate/nitrite accumulation in skeletal muscle. Although there was no apparent increase in eNOS or iNOS with the loss of nNOS in skeletal muscle, the potential increase in the activation of the nitrate/nitrite reductive pathway provides evidence for increased contribution from the nitrate/nitrite reductive pathway to nitrate/nitrite accumulation in skeletal muscle in nNOS−/−mice treated with high-nitrate water or low-nitrate diet, or in combination. This supports the notion that NO synthases do not participate in or are not the primary contributors to the metabolism of nitrate/nitrite for NO production under nitrate supplementation. These changes appear to be specific to skeletal muscle, and significant differences in the expression of NO synthase-related genes and nitrate/nitrite reductive pathway-related genes were not observed in the liver of both WT and nNOS−/− mice. We previously found that rats also show no changes in the nitrate/nitrite reductive pathway-related gene expression (*sialin*, *CLC2*, and *XOR*) in the liver after nitrate supplementation (Park et al., 2023). The contribution of the nitrate/nitrite reductive pathway to NO production in skeletal muscle might be more robust than in other organs (Piknova et al., 2016a; Piknova et al., 2022b). In the skeletal muscle of nNOS−/− mice, nitrate supplementation involved the nitrate/nitrite reductive pathway, whereas in their liver, nitrate supplementation did not alter the expression of the nitrate/nitrite reductive pathway. This suggests that nitrate supplementation influences the compensatory nitrate/nitrite reductive pathway in skeletal muscle more than in the liver.

In this study, the quantification of mRNA expression demonstrated that nitrate supplementation is involved in NO production via either NO synthases or the nitrate/nitrite reductive pathway; however, from protein expression, the nitrate/nitrite reductive pathway showed greater effects than NO synthases. There were no changes in iNOS and eNOS protein levels and eNOS activation activity [p-eNOS (Ser1177)]. In addition, a trend of inhibition of eNOS activity by increasing p-eNOS (Thr495) was shown in nNOS−/− mice placed on a low-nitrate diet (*p* = 0.0241, unpaired *t*-test) and with low-nitrate pretreatment followed by high-nitrate supplementation (*p* = 0.0226, unpaired *t*-test). nNOS−/− mice treated with high nitrate showed a trend to increase XOR protein (*p* = 0.0583, unpaired *t*-test), while nNOS−/− mice treated with low nitrate revealed a trend to increase sialin protein (*p* = 0.0594, unpaired *t*-test). Since skeletal muscle protein expression of sialin and XOR is highly variable between individuals, as shown in our previous study in rats and humans (Wylie et al., 2019; Piknova et al., 2023), a significant difference in protein expression in nitrate treatments could not be notably observed. Additionally, limitations of antibody suitability and availability for Western blotting result in changes more readily detectable by mRNA expression. It is interesting that the inhibition of sialin and CLC1 by an anion transporter inhibitor [4,4′-diisothiocyanatostilbene-2,2′-disulfonic acid disodium salt hydrate (DIDS)] inhibits nitrate uptake in myotubes (Srihirun et al., 2020), while the inhibition of XOR by an XOR inhibitor (oxypurinol) decreases nitrate reductase activity and NO generation in the rat skeletal muscle homogenate (Piknova et al., 2016a; Park et al., 2021b). Therefore, the presence of sialin and XOR in skeletal muscle can imply that the nitrate/nitrite reductive pathway is active and essential for NO production, as described in the human study with nitrate supplementation (Wylie et al., 2019). However, the role of XOR as the potential main source of NO in the nNOS−/− mice can be addressed by further studies using XOR inhibitors.

Although not investigated here, the response of NO synthases and the nitrate/nitrite reductive pathway to nitrate supplementation dependent on skeletal muscle fiber type warrants further investigation. Responses to nitrate supplementation can vary according to skeletal muscle type. For example, in rats, the expression of sialin and XOR proteins in extensor digitorum longus (EDL) and soleus skeletal muscles is also not consistently changed by high-nitrate treatment; only EDL shows upregulation of XOR protein (Park et al., 2021b). Fast-twitch and slow-twitch muscle fibers can exhibit differences in protein expression, such as higher expression of chloride channel CLC1 in human fast-twitch type IIa compared with slow-twitch type I muscle fibers (Thomassen et al., 2018). In rodents, contractile force and intracellular free calcium were significantly higher in fast-twitch muscles such as EDL and flexor digitorum brevis (FDB) with nitrate treatment with an increased calcium-binding protein (calsequestrin 1) and a calcium channel [dihydropyridine receptor (DHPR)], but not in slow-twitch soleus muscles (Hernandez et al., 2012).

In conclusion, our findings indicate that nitrate supplementation resulting in high-nitrate levels in skeletal muscle may allow nitrate/nitrite reductive processes to compensate for the loss of nNOS in the skeletal muscle of nNOS knockout mice. Although this study was performed in a resting condition without exercise, we believe that these findings would be a potential application for nNOS deficiency or diseases associated with nNOS malfunction.

## Data Availability

The raw data supporting the conclusion of this article will be made available by the authors, without undue reservation.

## Abbreviations

CLC1, chloride channel 1; CLC2, chloride channel 2; eNOS, endothelial nitric oxide synthase; GAPDH, glyceraldehyde-3-phosphate dehydrogenase; H-NO_3_^-^, high-nitrate water; IL6, interleukin 6; iNOS, inducible nitric oxide synthase; L-NO_3_^-^, low-nitrate diet; LH-NO_3_^-^, low-nitrate diet followed by high-nitrate water; nNOS, neuronal nitric oxide synthase; nNOS−/−, nNOS knockout; NO, nitric oxide; p-eNOS, phospho-eNOS; Rpl13a, ribosomal protein L13a; WT, wild-type; XOR, xanthine oxidoreductase.
