# Supplementary material for: High nitrate levels in skeletal muscle contribute to nitric oxide generation via a nitrate/nitrite reductive pathway in mice that lack the nNOS enzyme
Source: Front Physiol. 2024 May 9;15:1352242. doi: 10.3389/fphys.2024.1352242 (PMC11112080; doi:10.3389/fphys.2024.1352242)
Supplement: Supplementary file 1 [file Table1.docx]

Supplementary Tables

**Supplementary Table S1.** The list of mouse primer sequences

| **Gene name** | **Gene symbol** | **NCBI transcript accession number** | **Primer sequence (5’-3’)** | **Amplicon length (bp)** |
| --- | --- | --- | --- | --- |
| Nitric oxide synthase 1, neuronal (Nos1) | *nNOS* | NM_008712 | Forward: GATGACAACCGGTACCACGA  Reverse: GGCGGGAGGATCCAGTTAGG | 150 |
| Nitric oxide synthase 3, endothelial cell (Nos3) | *eNOS* | NM_008713 | Forward: AAGGTGATGAGCTCTGTGGC  Reverse: GATATCTCGGGCAGCAGCTT | 119 |
| Nitric oxide synthase 2, inducible (Nos2) | *iNOS* | NM_010927 | Forward: CAGCTGGGCTGTACAAACCTT  Reverse: CATTGGAAGTGAAGCGTTTCG | 95 |
| Interleukin 6 | *IL6* | NM_031168 | Forward: TACCACTTCACAAGTCGGAGGC  Reverse:  CTGCAAGTGCATCATCGTTGTTC | 116 |
| Solute carrier family 17 member 5 (Slc17a5) | *Sialin* | NM_172773 | Forward: GGAACATTCTGCCCCCATAAAAG  Reverse:  CTGGGTGACGATGTAGCCG | 118 |
| Chloride channel, voltage-sensitive 1 (Clcn1) | *CLC1* | NM_013491 | Forward: ACAATGCCCACCCAACACA  Reverse: GTCCTCATCCAAGCTGTCCA | 122 |
| Chloride channel, voltage-sensitive 2 (Clcn2) | *CLC2* | NM_009900 | Forward: AGATTGTCCAGGTGATGCGG  Reverse: TGAACTGTCCAAAGCCAGGG | 129 |
| Xanthine dehydrogenase (Xdh) | *XOR* | NM_011723 | Forward: ACAACGGTAGATGAGTTGGTCT  Reverse:  AAGCTTGGTCCCGCACAG | 123 |
| Ribosomal protein L13A (Rpl13a) | *Rpl13a* | NM_009438 | Forward: GCTTCTTCTTCCGATAGTGCATC  Reverse: AGCCTACCAGAAAGTTTGCTTAC | 129 |

**Supplementary Table S2.** The list of primary antibodies

| **Name** | **Company** | **Cat #** | **Size**  **(kDa)** |
| --- | --- | --- | --- |
| nNOS | Cell Signaling | 4231S | 160 |
| eNOS | Cell Signaling | 32027 | 140 |
| Phospho-eNOS (Ser1177) | Cell Signaling | 9571S | 140 |
| Phospho-eNOS (Thr495) | Cell Signaling | 9574S | 140 |
| iNOS | Cell Signaling | 32027 | 130 |
| Sialin | Alpha Diagnostic | SIAL11-A | 55 |
| XOR | Abcam | ab133268 | 146 |
| GAPDH | Cell Signaling | 97166 | 37 |
