# Supplementary material for: High nitrate levels in skeletal muscle contribute to nitric oxide generation via a nitrate/nitrite reductive pathway in mice that lack the nNOS enzyme
Source: Front Physiol. 2024 May 9;15:1352242. doi: 10.3389/fphys.2024.1352242 (PMC11112080; doi:10.3389/fphys.2024.1352242)
Supplement: Supplementary file 2 [file DataSheet3.docx]

Supplementary Figures


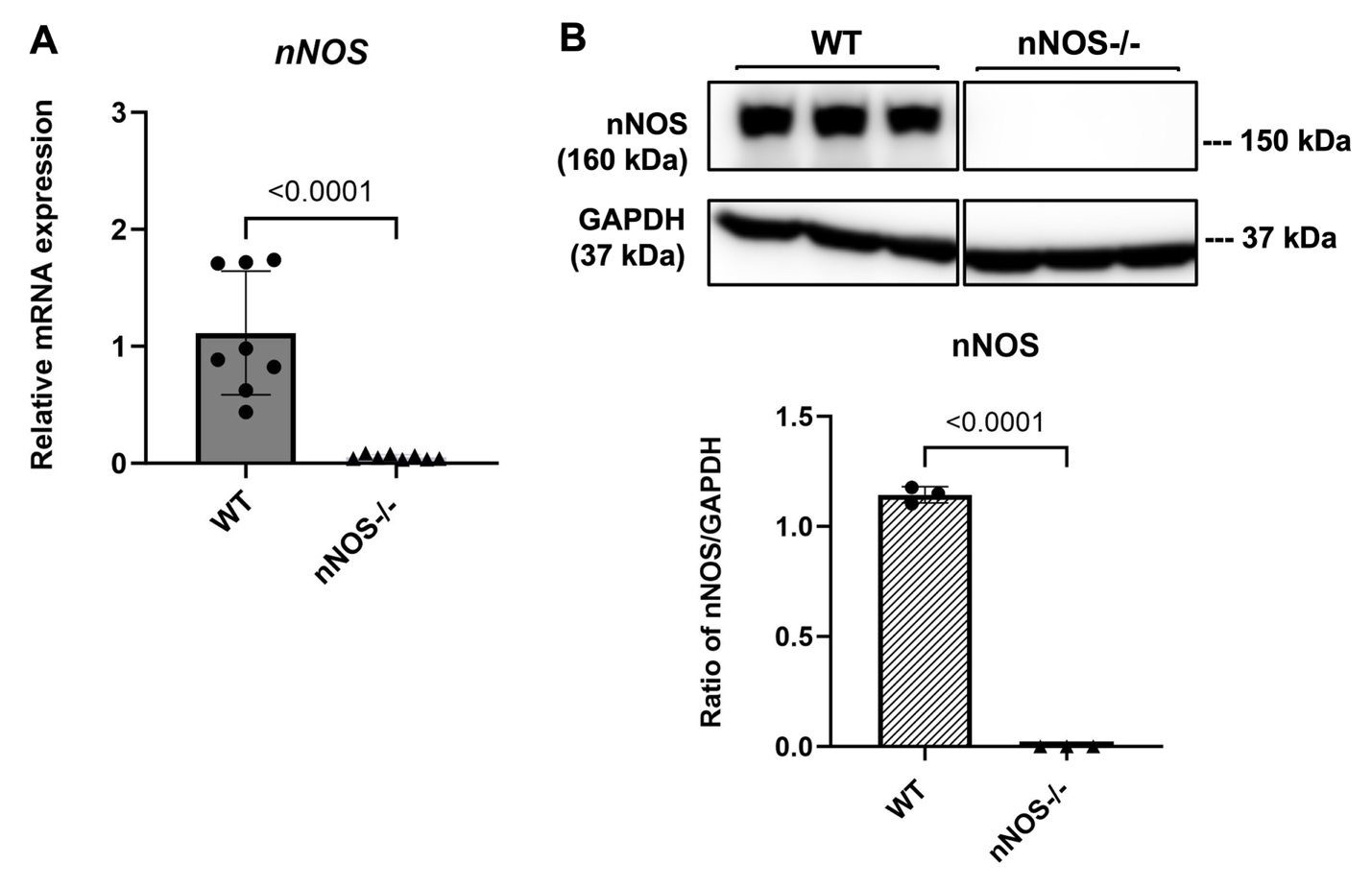


**Supplementary Figure S1.** **Baseline levels** **of *nNOS* mRNA and protein expression in skeletal muscle of WT and nNOS-/- control mice.** *nNOS* mRNA (n = 8; 4 male mice and 4 female mice) **(A)** and protein expressions (n = 3; 2 male mice and 1 female mouse) **(B)** in quadriceps skeletal muscle of WT and nNOS-/- mice were quantified. Relative mRNA expression was acquired by normalizing to *Rpl13a* and is presented as a fold change. Protein expression is presented as a ratio of band density normalized to GAPDH. Data are mean ± SD. The statistics was tested using the unpaired *t*-test. Note that the values of mRNA expression **(A)** and protein expression **(B)** in WT and nNOS-/- control groups were identical to the values in Figures 5 and 6A, respectively. The protein band image **(B)** is cropped and reused images of WT and nNOS-/- control mice in high- nitrate water supplementation in Figure 6A for the illustrative purpose, comparing nNOS protein between WT and nNOS-/- mice.

**
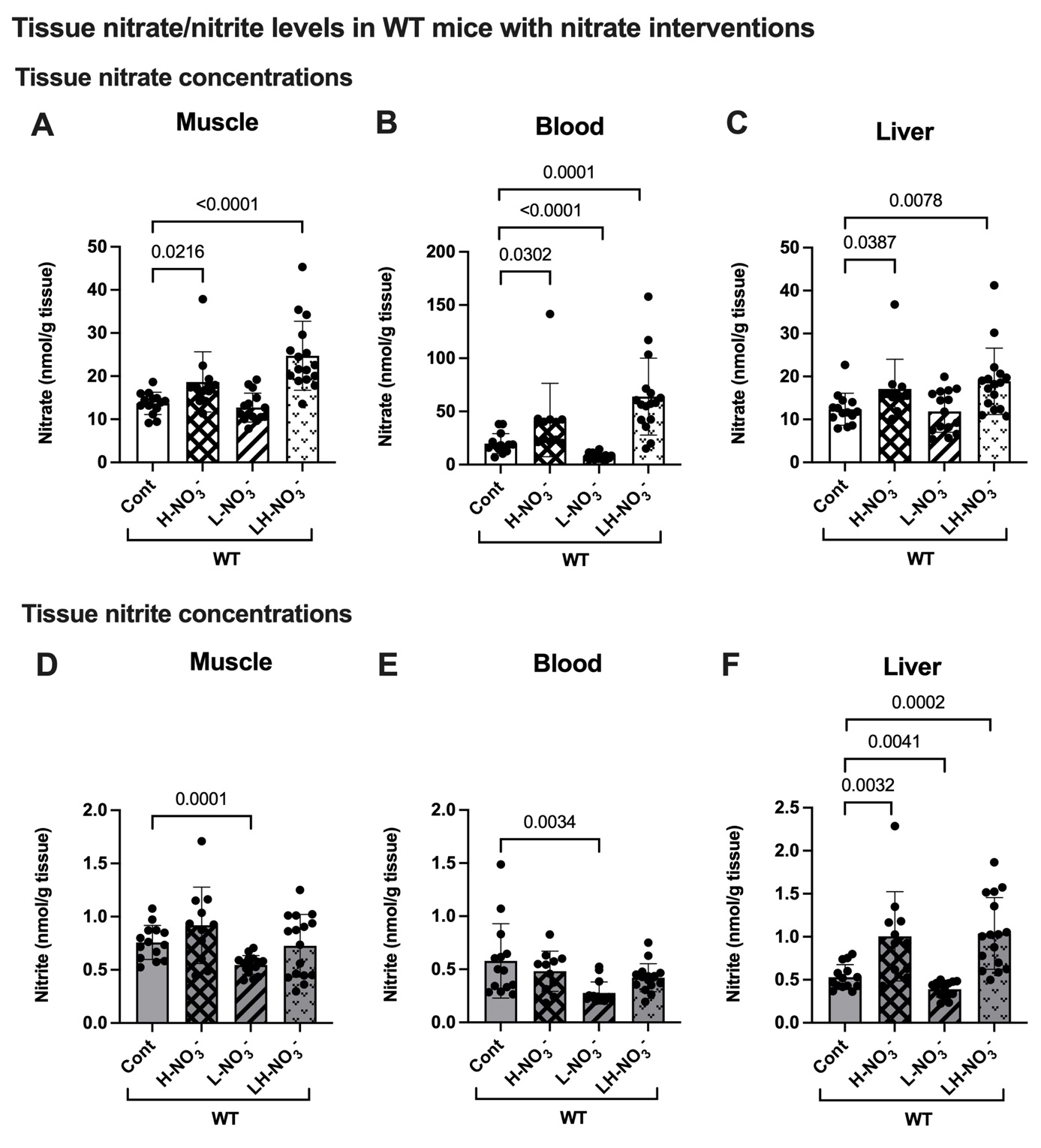
**

**Supplementary Figure S2. Tissue nitrate/nitrite levels in WT mice with nitrate interventions.** Tissue nitrate **(A-C)** and nitrite **(D-F)** concentrations were measured from quadriceps skeletal muscle, blood, and liver of WT mice with three nitrate interventions compared to normal chow control (Cont) (n = 14; 7 male mice and 7 female mice): high-nitrate water (1 g/L sodium nitrate for 7 days, H-NO_3_^-^) (n = 11; 6 male mice and 5 female mice), low-nitrate diet for 7 days
(L-NO_3_^-^) (n = 15; 8 male mice and 7 female mice) and low-nitrate diet for 7 days followed by high-nitrate water for 7 days (LH-NO_3_^-^) (n = 16; 8 male mice and 8 female mice). Data are mean ± SD. Note that the data were identical to the values in Figures 3 and 4 while the statistics was tested using the unpaired *t*-test compared to the WT control group.

**
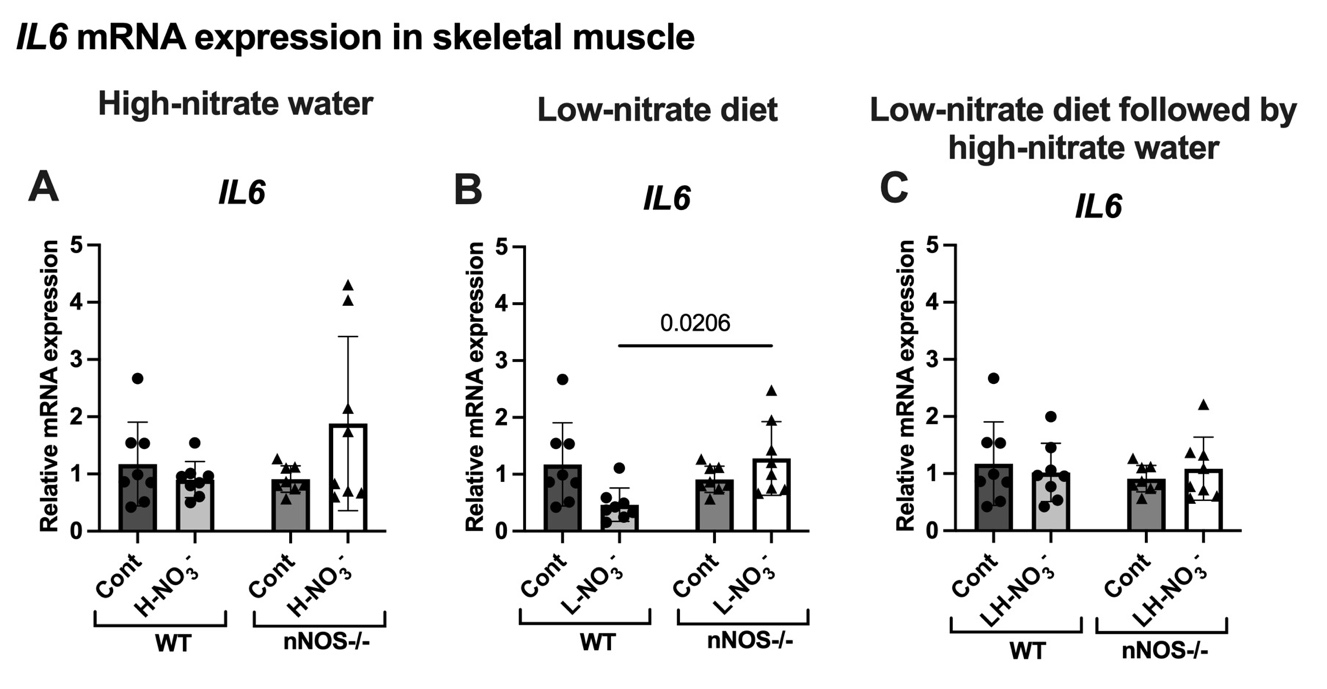
**

**Supplementary Figure S3. *IL6* mRNA expression in skeletal muscle.** *IL6* mRNA expression in quadriceps skeletal muscle was detected using qPCR after WT and nNOS-/- mice (n = 8; 4 male mice and 4 female mice) were treated with three nitrate interventions compared to normal chow control (Cont): high-nitrate water (1 g/L sodium nitrate for 7 days, H-NO_3_^-^) **(A)**, low-nitrate diet for 7 days (L-NO_3_^-^) **(B)** and low-nitrate diet for 7 days followed by high-nitrate water for 7 days (LH-NO_3_^-^) **(C)**. Relative mRNA expression was acquired by normalizing to *Rpl13a* and is presented as a fold change (mean ± SD) compared to WT control group. The statistics was tested using a two-way ANOVA with Tukey’s adjustment for multiple comparisons.


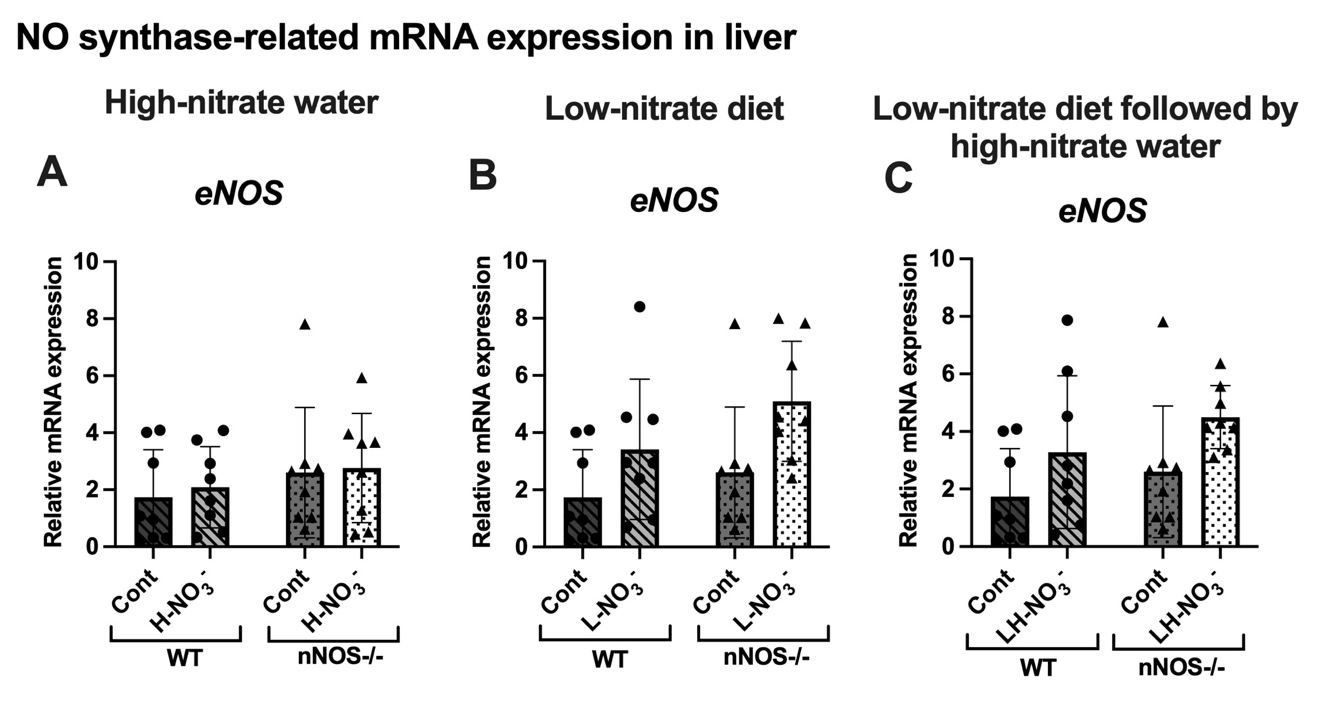


**Supplementary Figure S4.** **NO synthase-related mRNA expression in liver.** *eNOS* mRNA expression in liver was detected using qPCR after WT and nNOS-/- mice (n = 8; 4 male mice and 4 female mice) were treated with three nitrate interventions compared to normal chow control (Cont): high-nitrate water (1 g/L sodium nitrate for 7 days, H-NO_3_^-^) **(A)**, low-nitrate diet for 7 days
(L-NO_3_^-^) **(B)** and low-nitrate diet for 7 days followed by high-nitrate water for 7 days (LH-NO_3_^-^) **(C)**. Relative mRNA expression was acquired by normalizing to *Rpl13a* and is presented as a fold change (mean ± SD) compared to WT control group. The statistics was tested using a two-way ANOVA with Tukey’s adjustment for multiple comparisons. Note that mRNA expression of *nNOS* and *iNOS* is very low in liver of WT and nNOS-/- mice and was undetermined by qPCR.


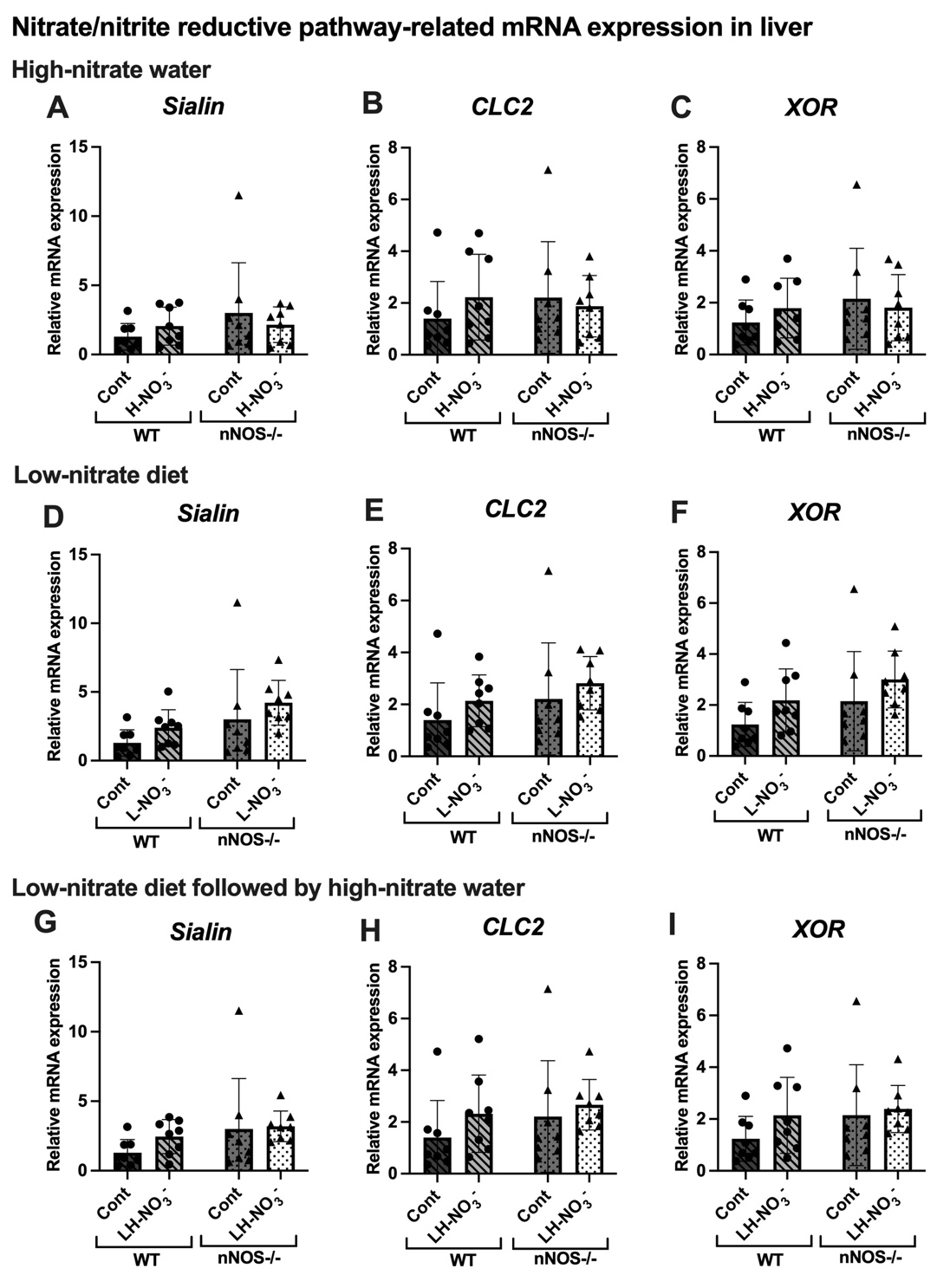


**Supplementary Figure S5.** **Nitrate/nitrite reductive pathway-related mRNA expression in liver.** mRNA expression of proteins related to nitrate/nitrite reductive pathway (nitrate transporters (*sialin* and *CLC2*) and nitrate/nitrite reductase (*XOR*)) in liver was detected using qPCR after WT and nNOS-/- mice (n = 8; 4 male mice and 4 female mice) were treated with three nitrate interventions compared to normal chow control (Cont): high-nitrate water (1 g/L sodium nitrate for 7 days, H-NO_3_^-^) **(A-C)**, low-nitrate diet for 7 days (L-NO_3_^-^) **(D-F)** and low-nitrate diet for 7 days followed by high-nitrate water for 7 days (LH-NO_3_^-^) **(G-I)**. Relative mRNA expression was acquired by normalizing to *Rpl13a* and is presented as a fold change (mean ± SD) compared to WT control group. The statistics was tested using a two-way ANOVA with Tukey’s adjustment for multiple comparisons.
