# Supplementary material for: High nitrate levels in skeletal muscle contribute to nitric oxide generation via a nitrate/nitrite reductive pathway in mice that lack the nNOS enzyme
Source: Front Physiol. 2024 May 9;15:1352242. doi: 10.3389/fphys.2024.1352242 (PMC11112080; doi:10.3389/fphys.2024.1352242)
Supplement: Supplementary file 3 [file DataSheet1.ZIP › Original western blot images/Protein bands and protein marker.pdf]

Protein bands compared to protein marker

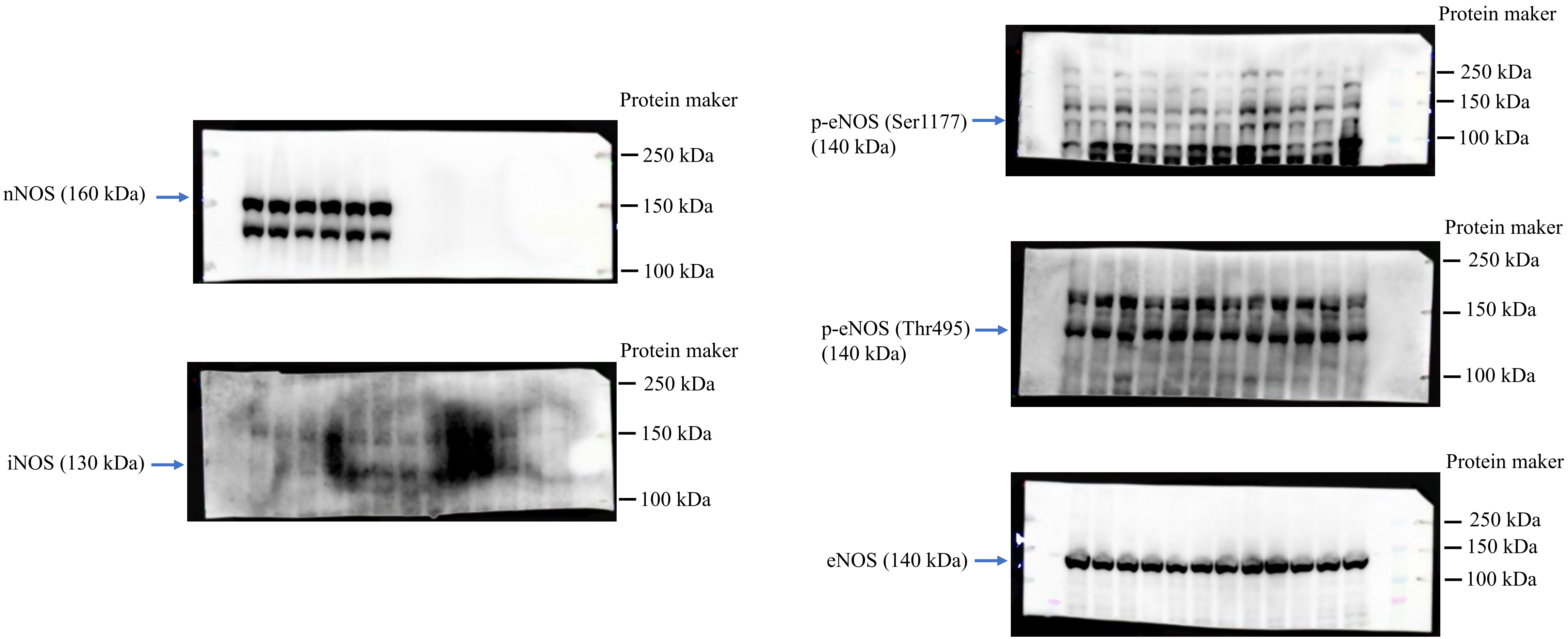

Note: To observe both protein bands and protein marker, these images were automatically exposed by 600-imager (azure biosystems, USA), merging between a protein band snapshot taken in chemiluminescence mode and a protein marker snapshot taken in color marker mode. The protein bands in the cropped images were located as blue arrows.

Protein bands compared to protein marker

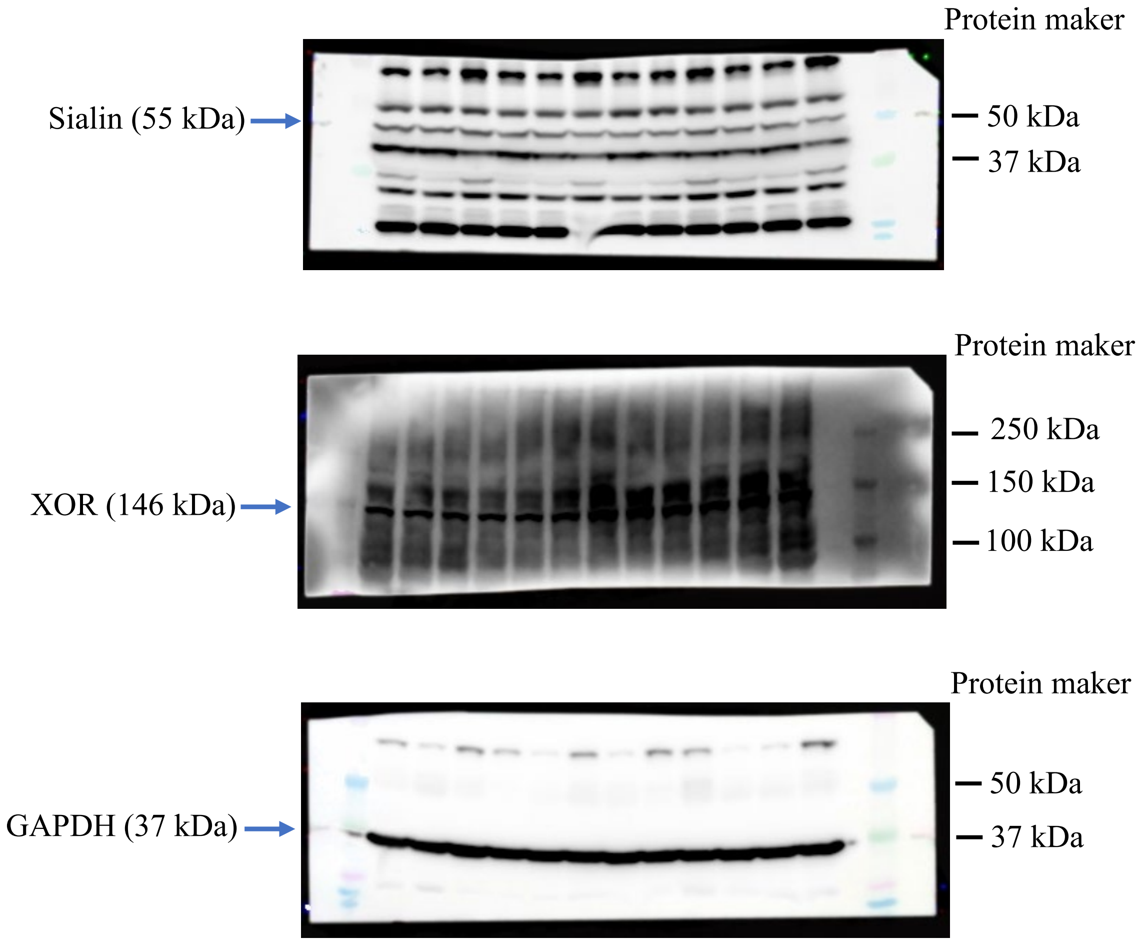

Note: To observe both protein bands and protein marker, these images were automatically exposed by 600-imager (azure biosystems, USA), merging between a protein band snapshot taken in chemiluminescence mode and a protein marker snapshot taken in color marker mode. The protein bands in the cropped images were located as blue arrows.
