# Supplementary figures and images for: High nitrate levels in skeletal muscle contribute to nitric oxide generation via a nitrate/nitrite reductive pathway in mice that lack the nNOS enzyme
Source: Front Physiol. 2024 May 9;15:1352242. doi: 10.3389/fphys.2024.1352242 (PMC11112080; doi:10.3389/fphys.2024.1352242)

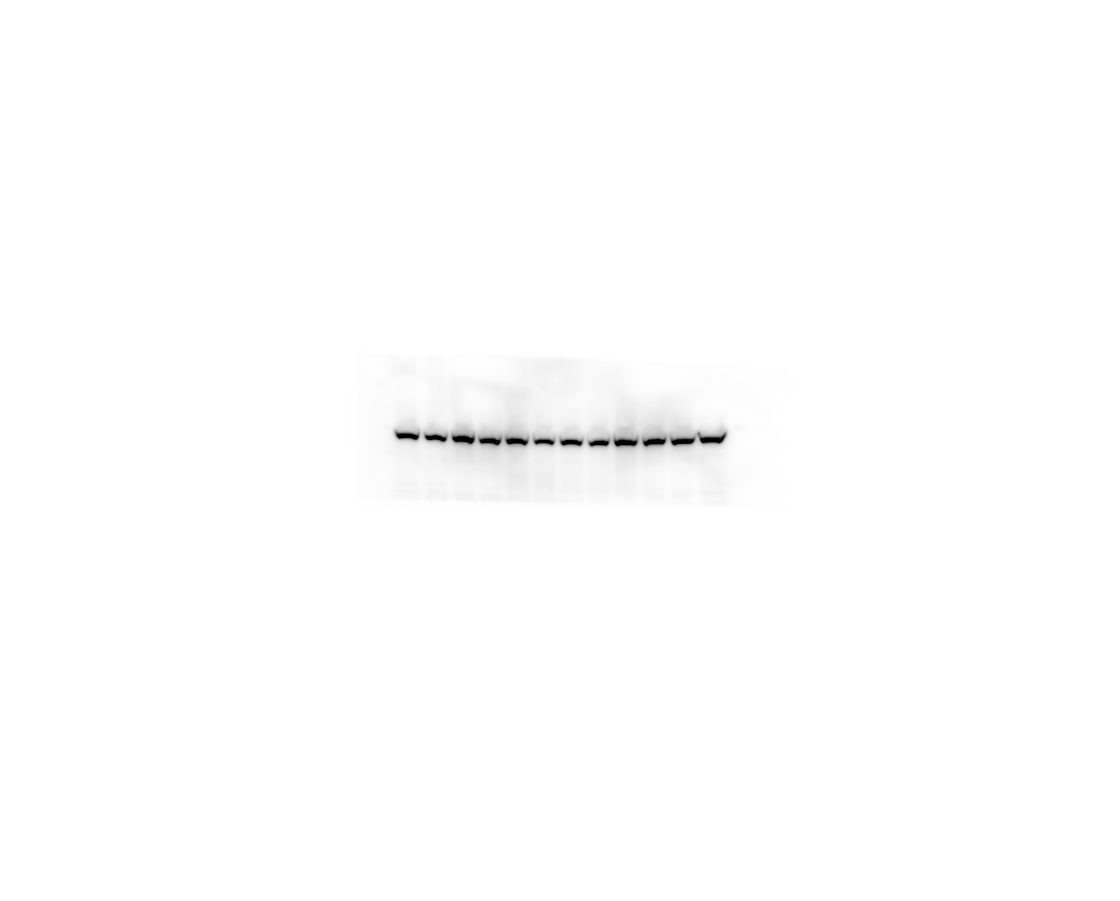

Supplement: Supplementary file 3 [file DataSheet1.ZIP › Original western blot images/High nitrate water/eNOS_High nitrate.tif]

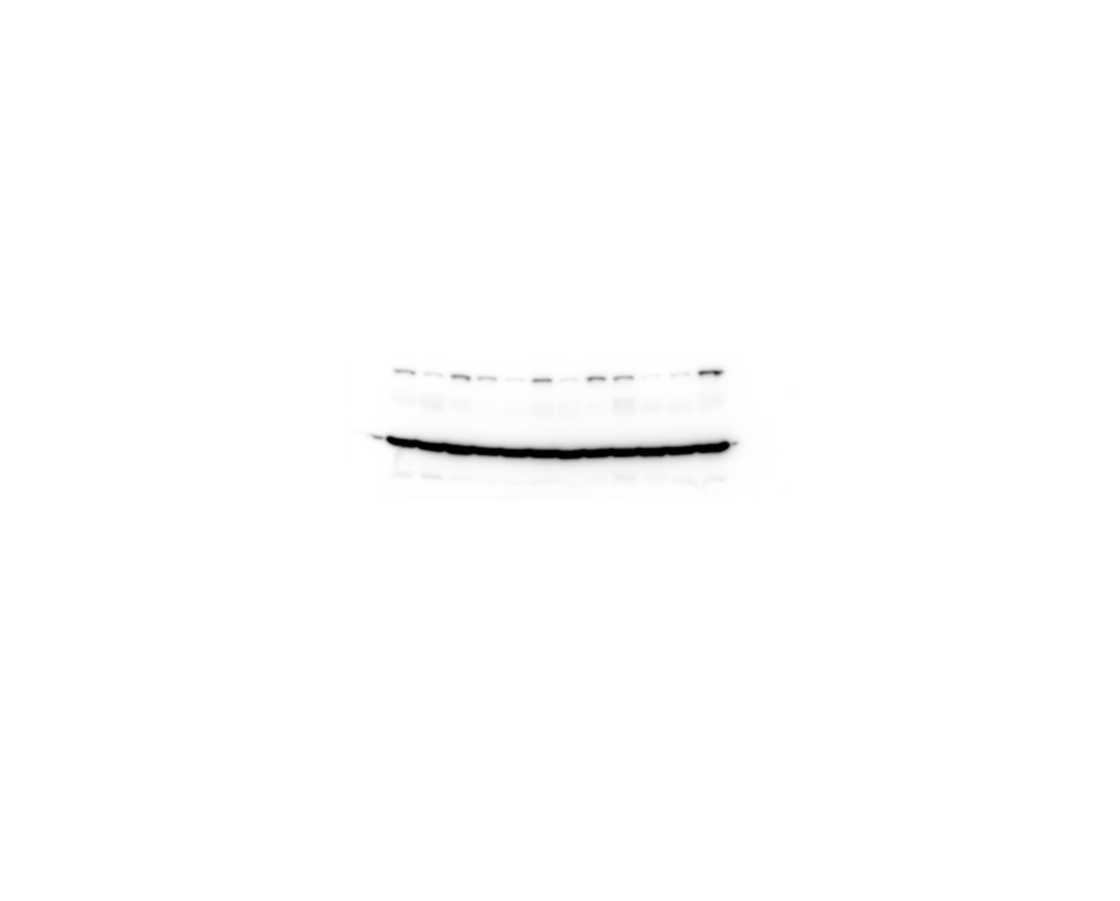

Supplement: Supplementary file 3 [file DataSheet1.ZIP › Original western blot images/High nitrate water/GAPDH_High nitrate.tif]

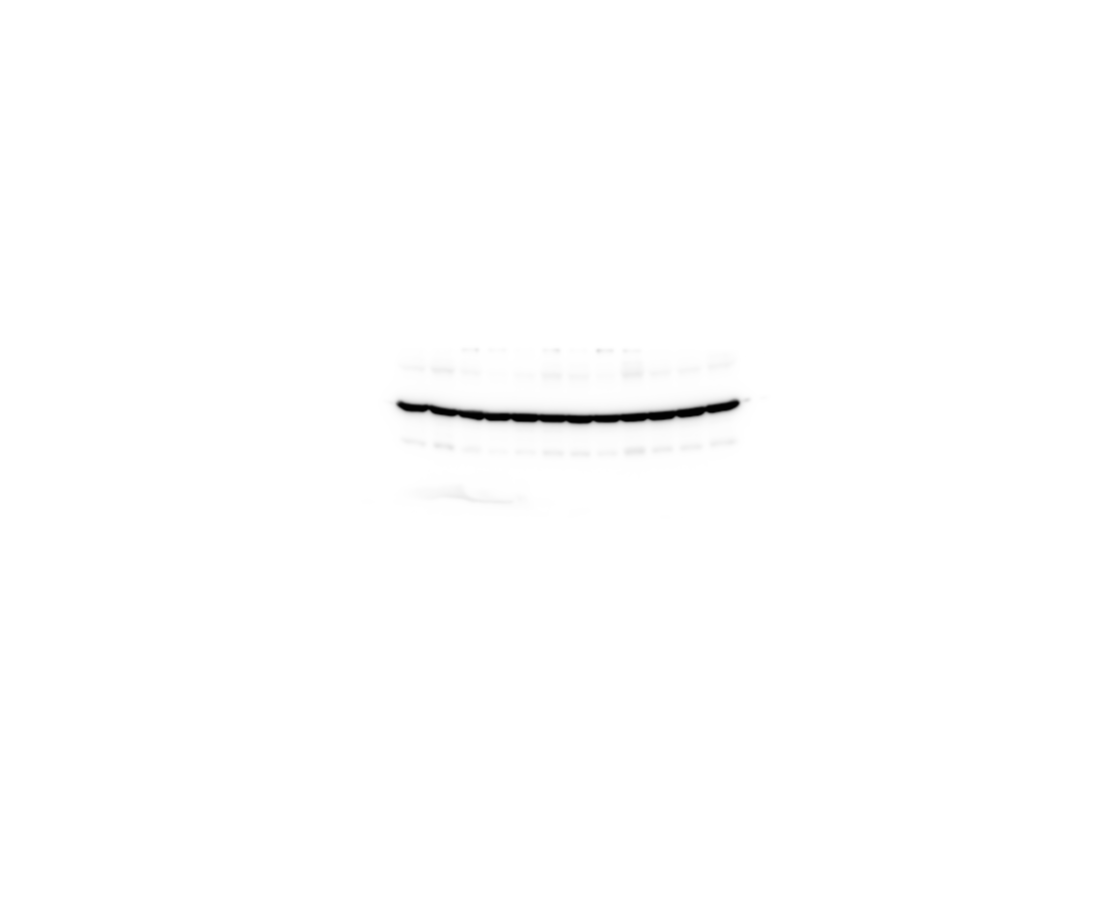

Supplement: Supplementary file 3 [file DataSheet1.ZIP › Original western blot images/High nitrate water/GAPDH-2_High nitrate.tif]

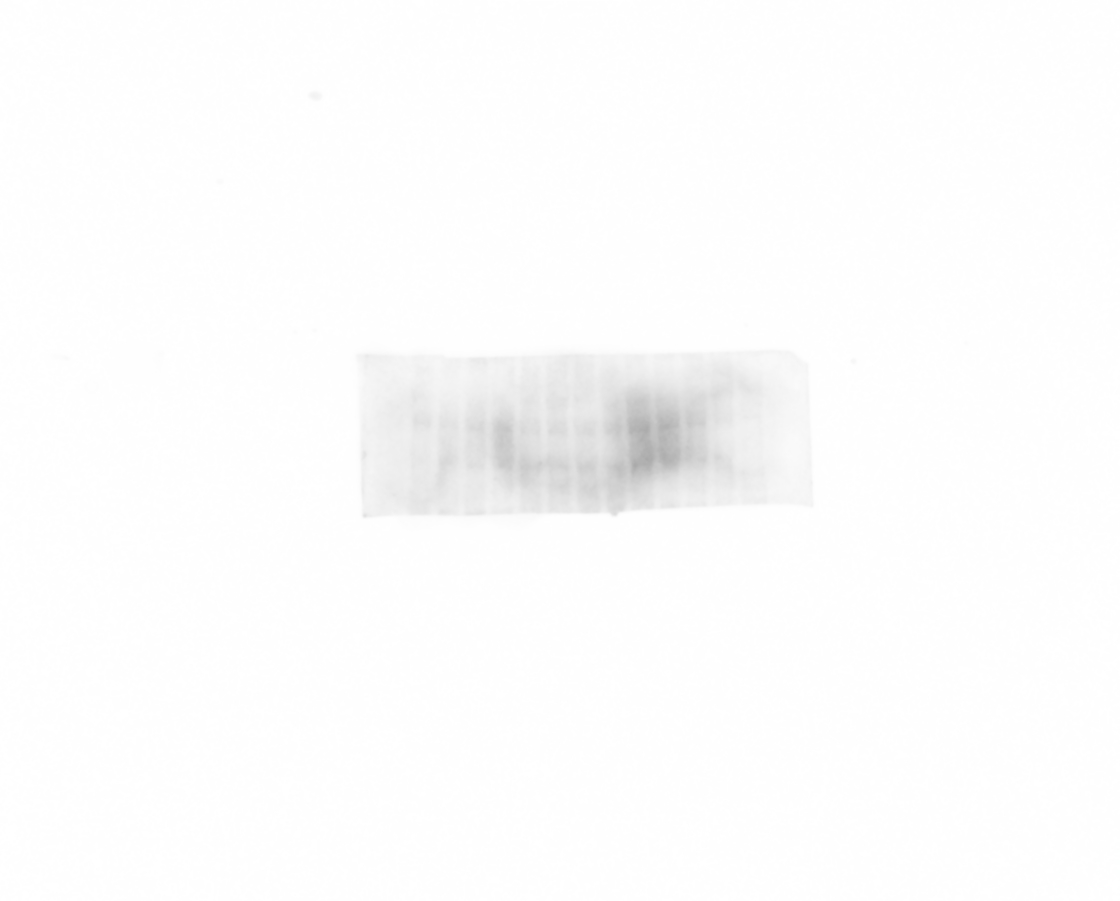

Supplement: Supplementary file 3 [file DataSheet1.ZIP › Original western blot images/High nitrate water/iNOS_High nitrate.tif]

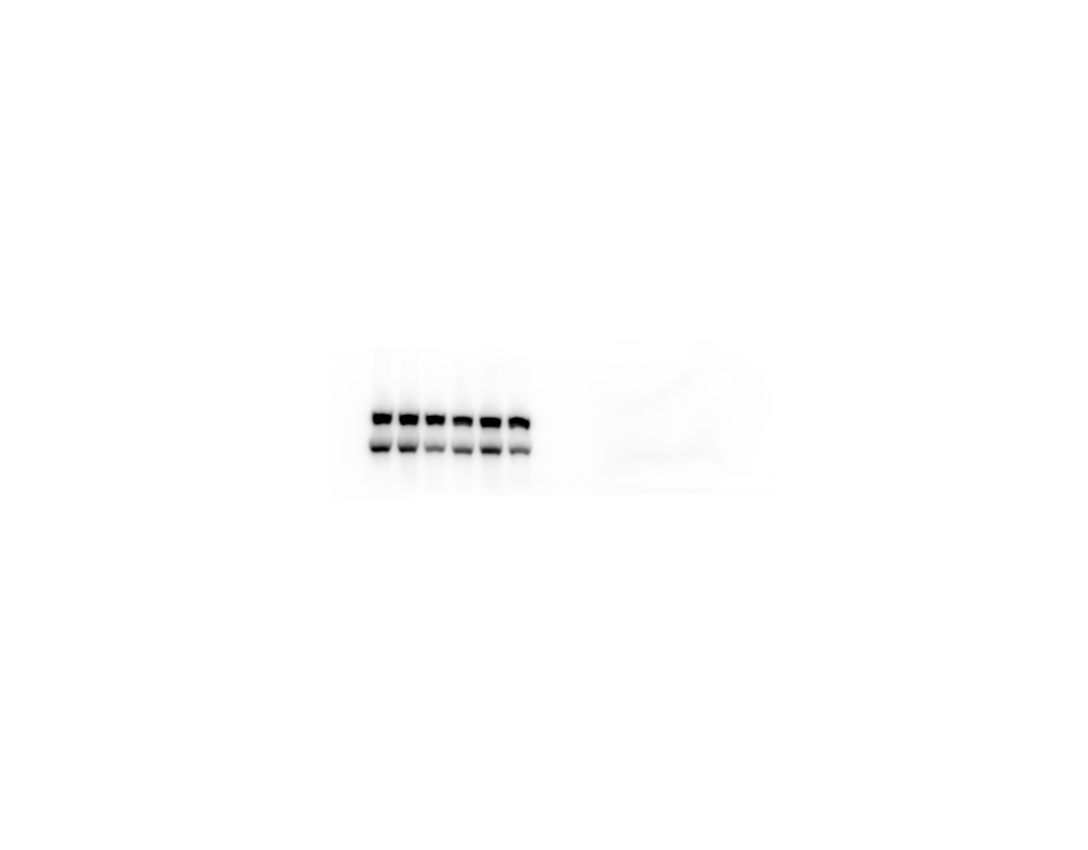

Supplement: Supplementary file 3 [file DataSheet1.ZIP › Original western blot images/High nitrate water/nNOS_High nitrate.tif]

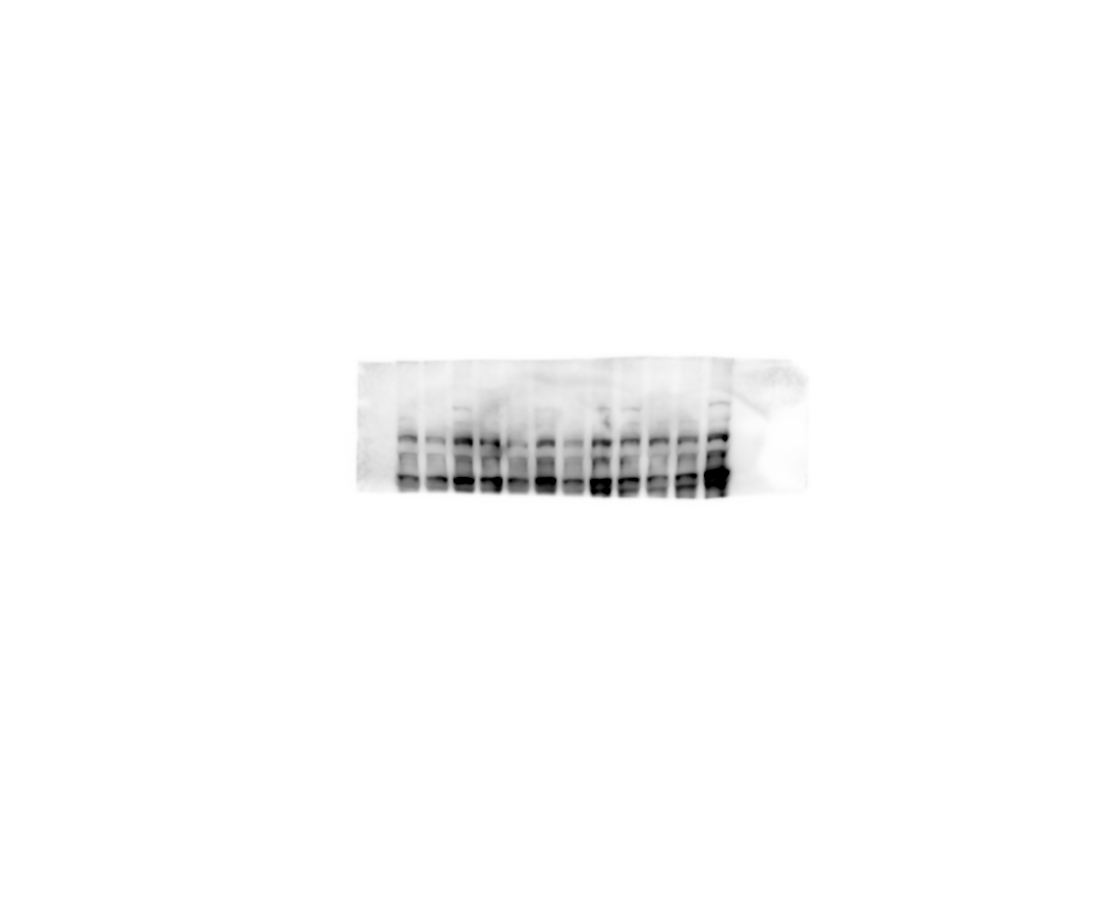

Supplement: Supplementary file 3 [file DataSheet1.ZIP › Original western blot images/High nitrate water/p-eNOSSer1177_High nitrate.tif]

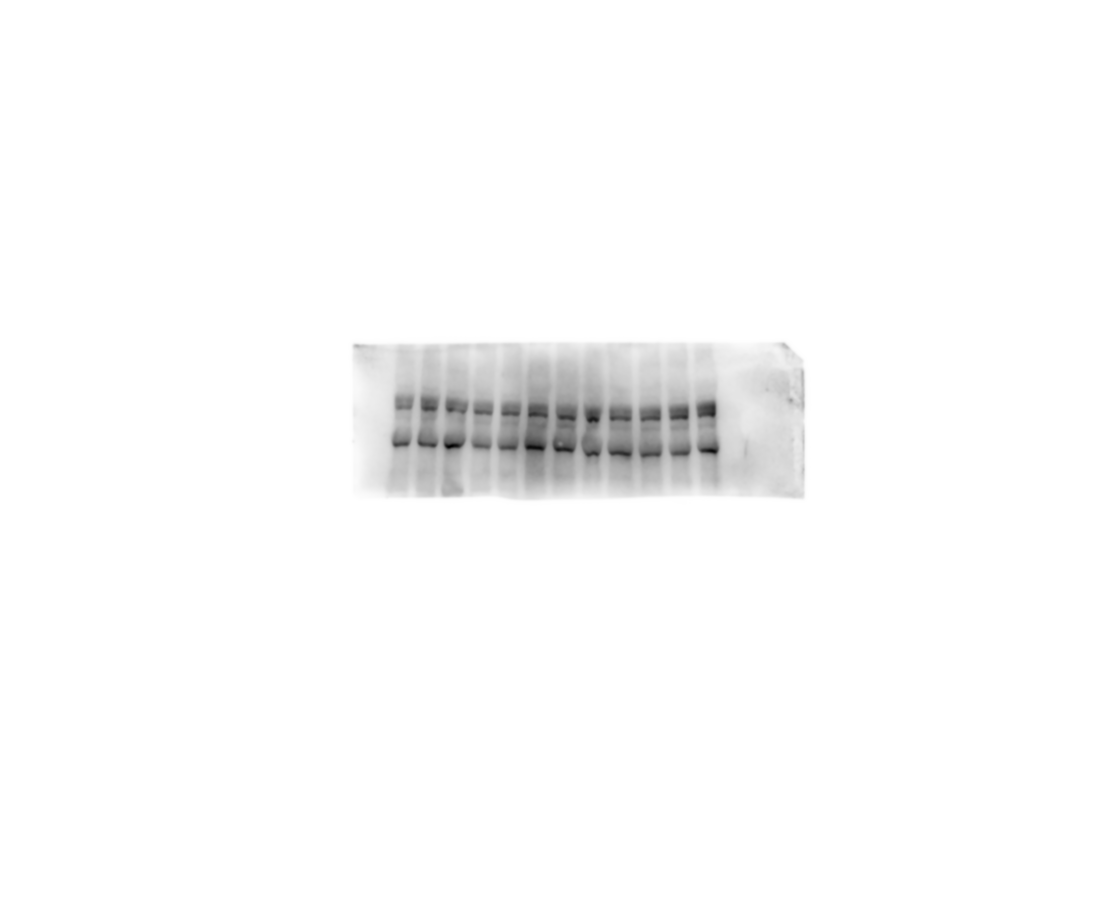

Supplement: Supplementary file 3 [file DataSheet1.ZIP › Original western blot images/High nitrate water/p-eNOSThr495_High nitrate.tif]

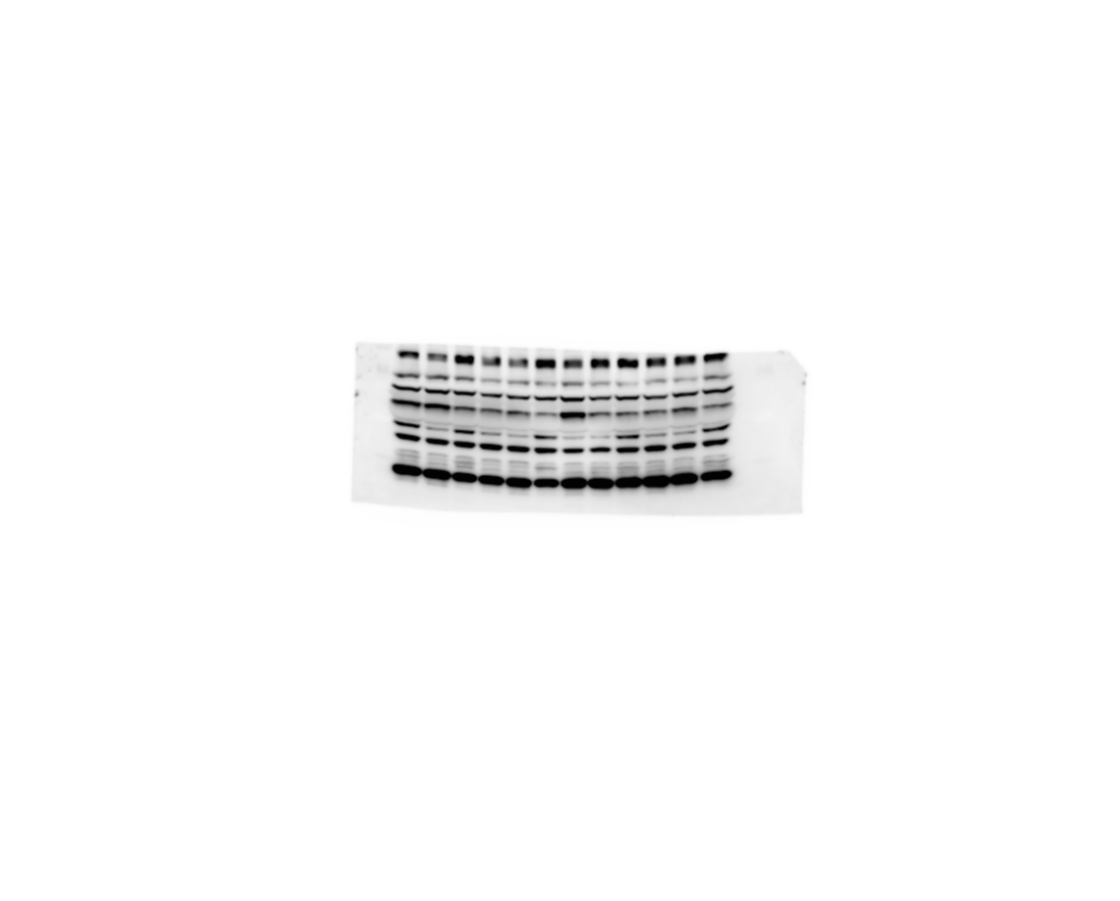

Supplement: Supplementary file 3 [file DataSheet1.ZIP › Original western blot images/High nitrate water/Sialin_High nitrate.tif]

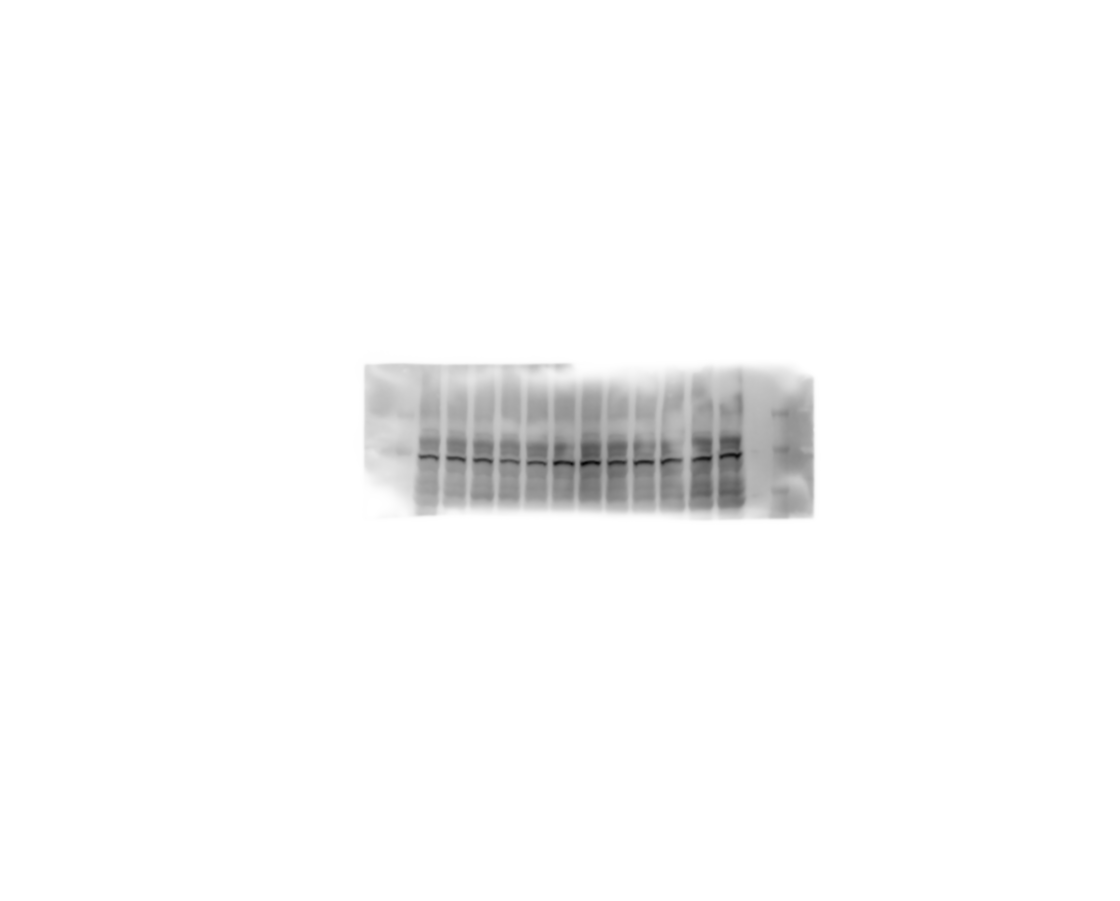

Supplement: Supplementary file 3 [file DataSheet1.ZIP › Original western blot images/High nitrate water/XOR_High nitrate.tif]

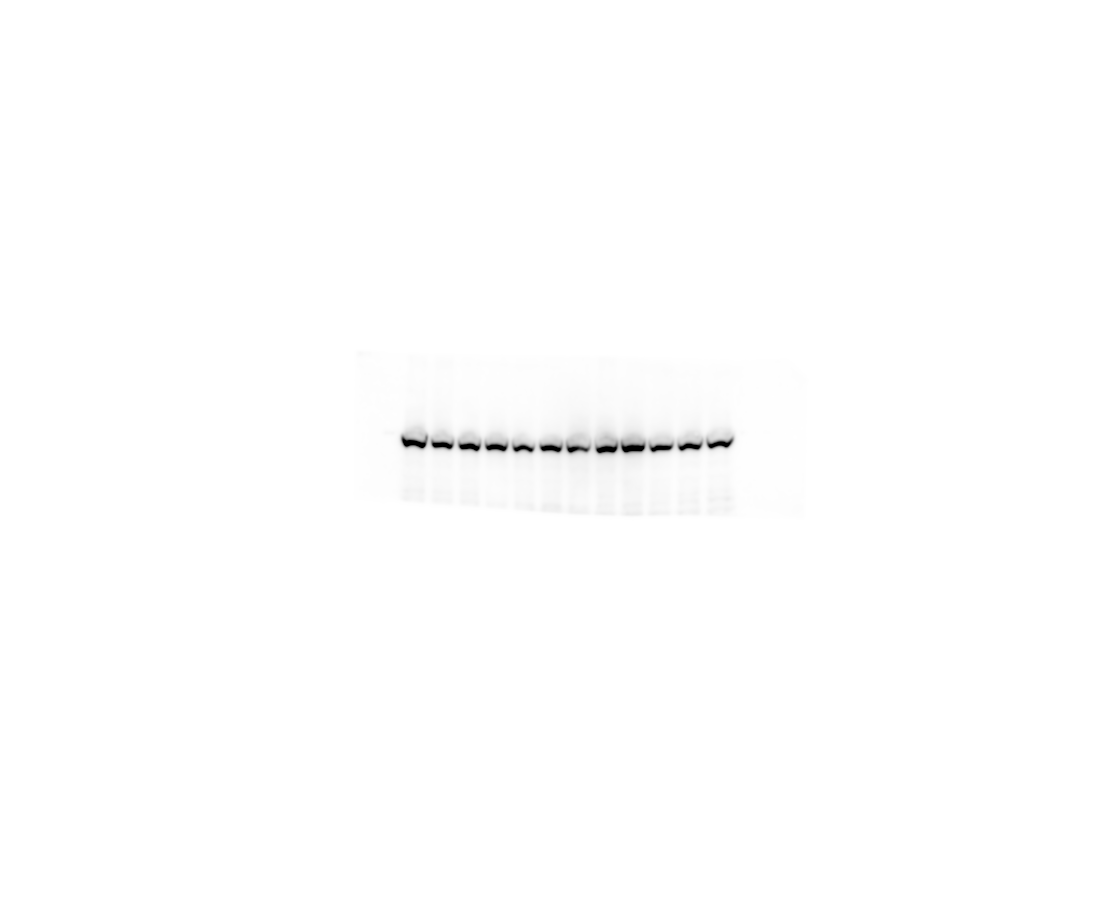

Supplement: Supplementary file 3 [file DataSheet1.ZIP › Original western blot images/Low nitrate diet followed by high nitrate water/eNOS_Low-high nitrate.tif]

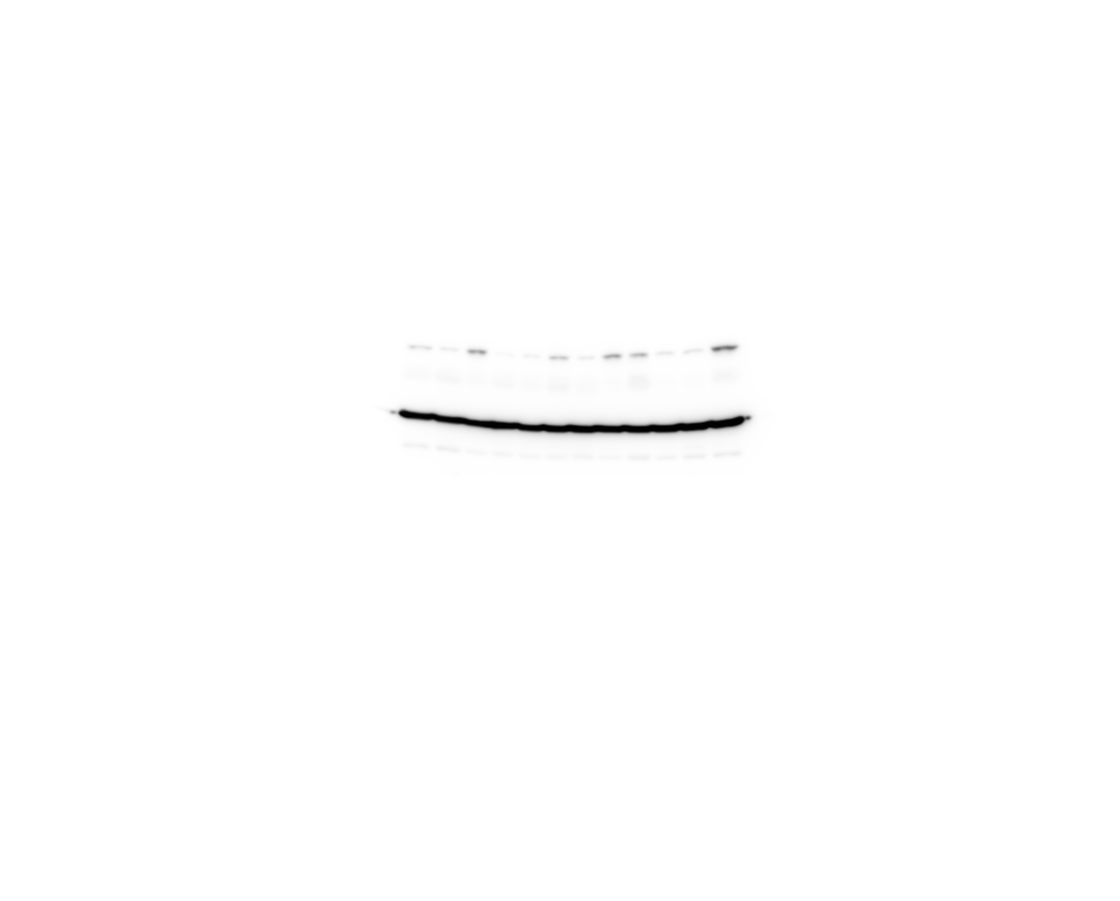

Supplement: Supplementary file 3 [file DataSheet1.ZIP › Original western blot images/Low nitrate diet followed by high nitrate water/GAPDH-1_Low-high nitrate.tif]

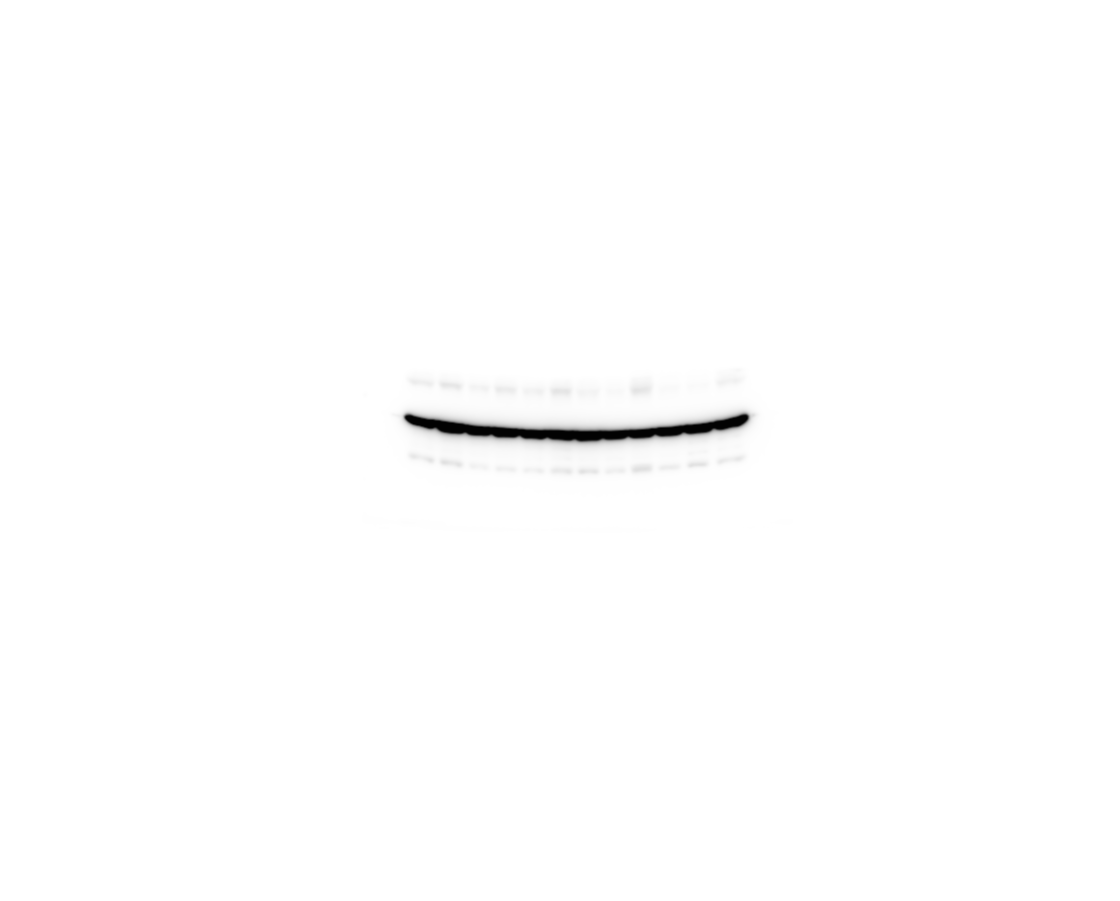

Supplement: Supplementary file 3 [file DataSheet1.ZIP › Original western blot images/Low nitrate diet followed by high nitrate water/GAPDH-2_Low-high nitrate.tif]

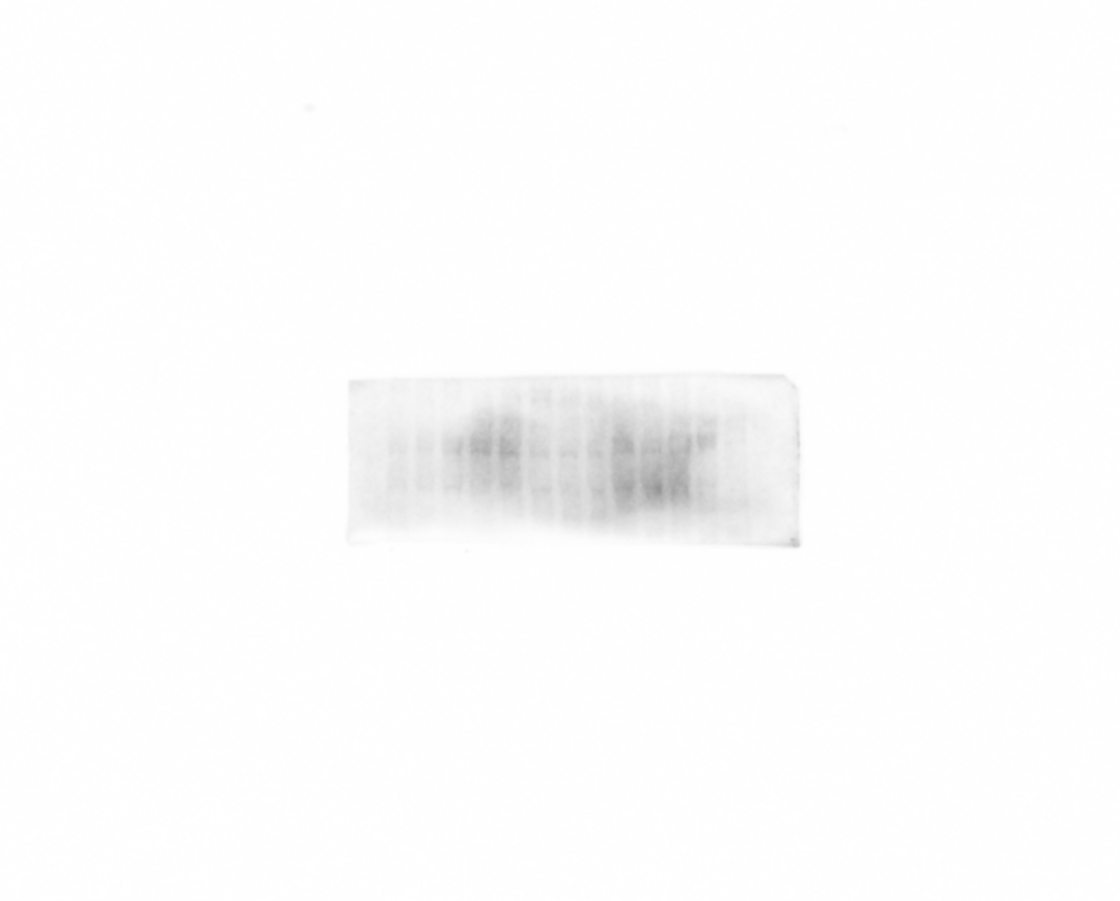

Supplement: Supplementary file 3 [file DataSheet1.ZIP › Original western blot images/Low nitrate diet followed by high nitrate water/iNOS_Low-high nitrate.tif]

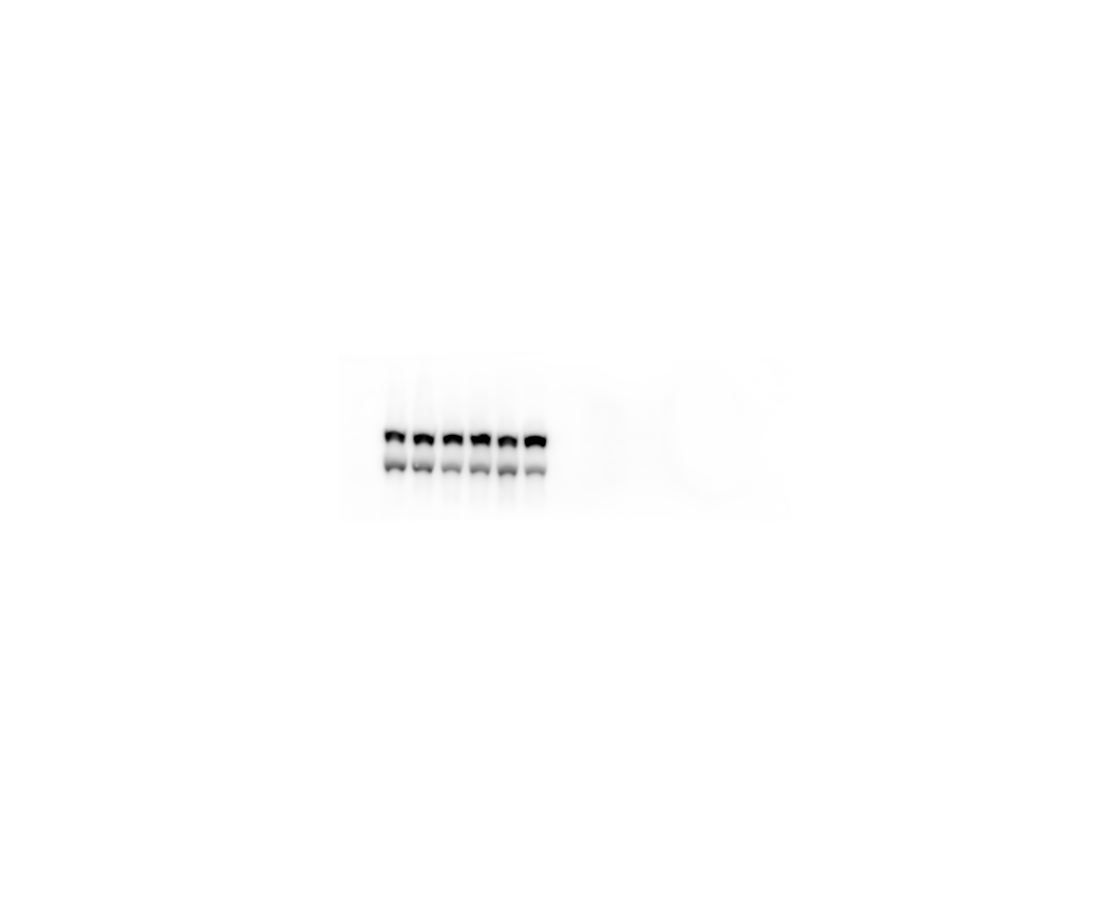

Supplement: Supplementary file 3 [file DataSheet1.ZIP › Original western blot images/Low nitrate diet followed by high nitrate water/nNOS_Low-high nitrate.tif]

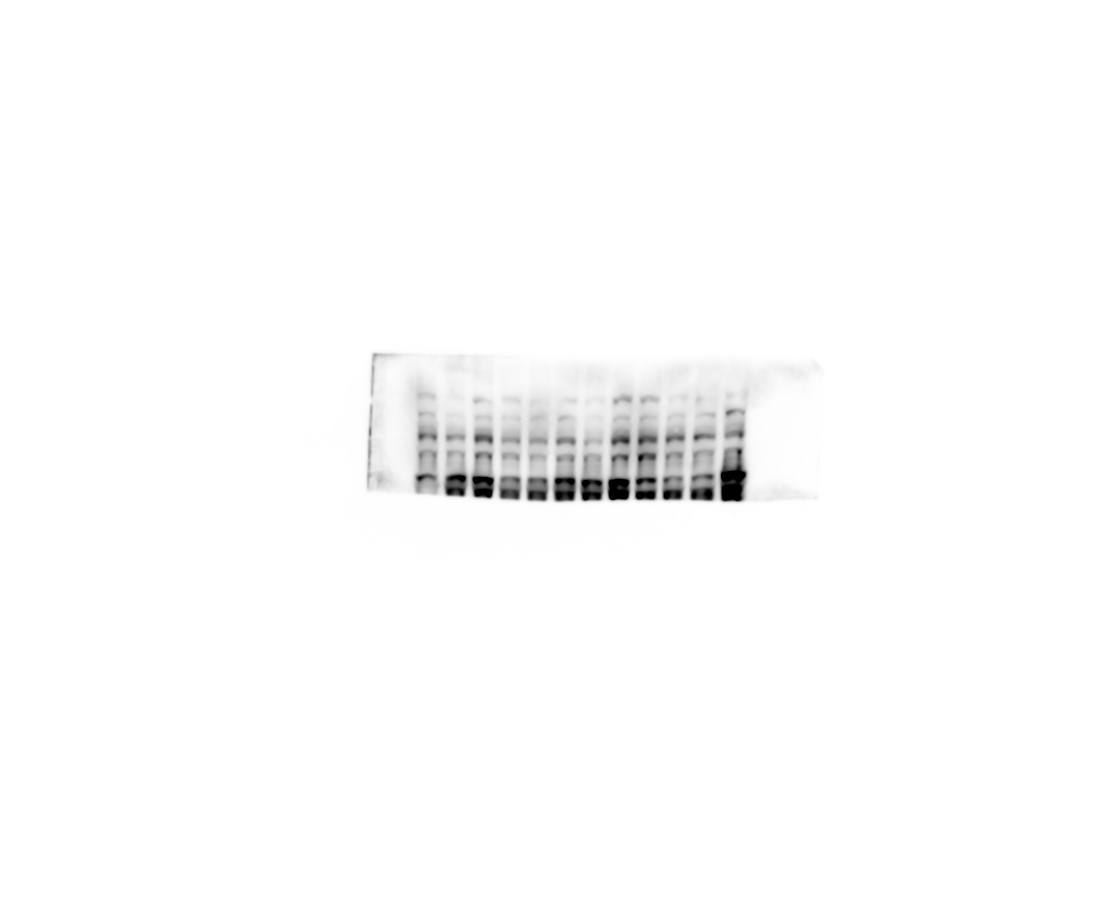

Supplement: Supplementary file 3 [file DataSheet1.ZIP › Original western blot images/Low nitrate diet followed by high nitrate water/p-eNOSSer1177_Low-high nitrate.tif]

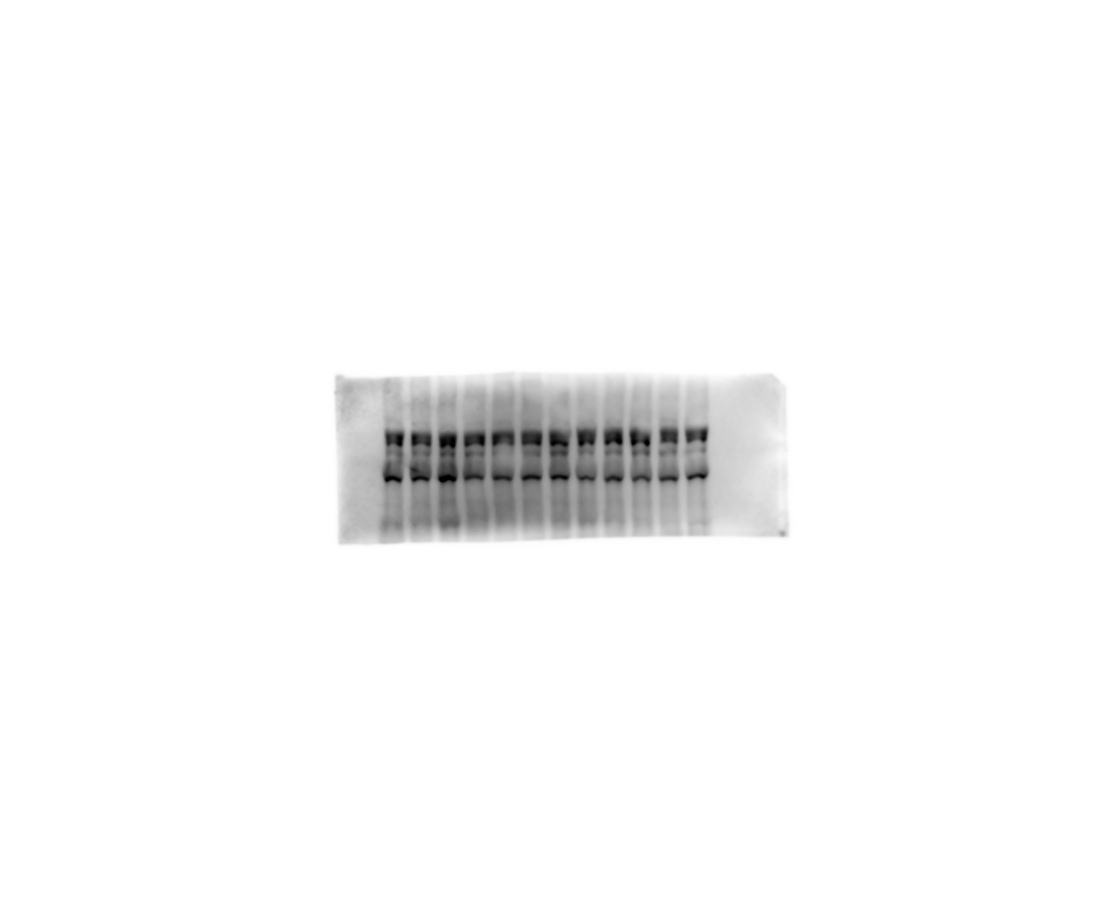

Supplement: Supplementary file 3 [file DataSheet1.ZIP › Original western blot images/Low nitrate diet followed by high nitrate water/p-eNOSThr495_Low-high nitrate.tif]

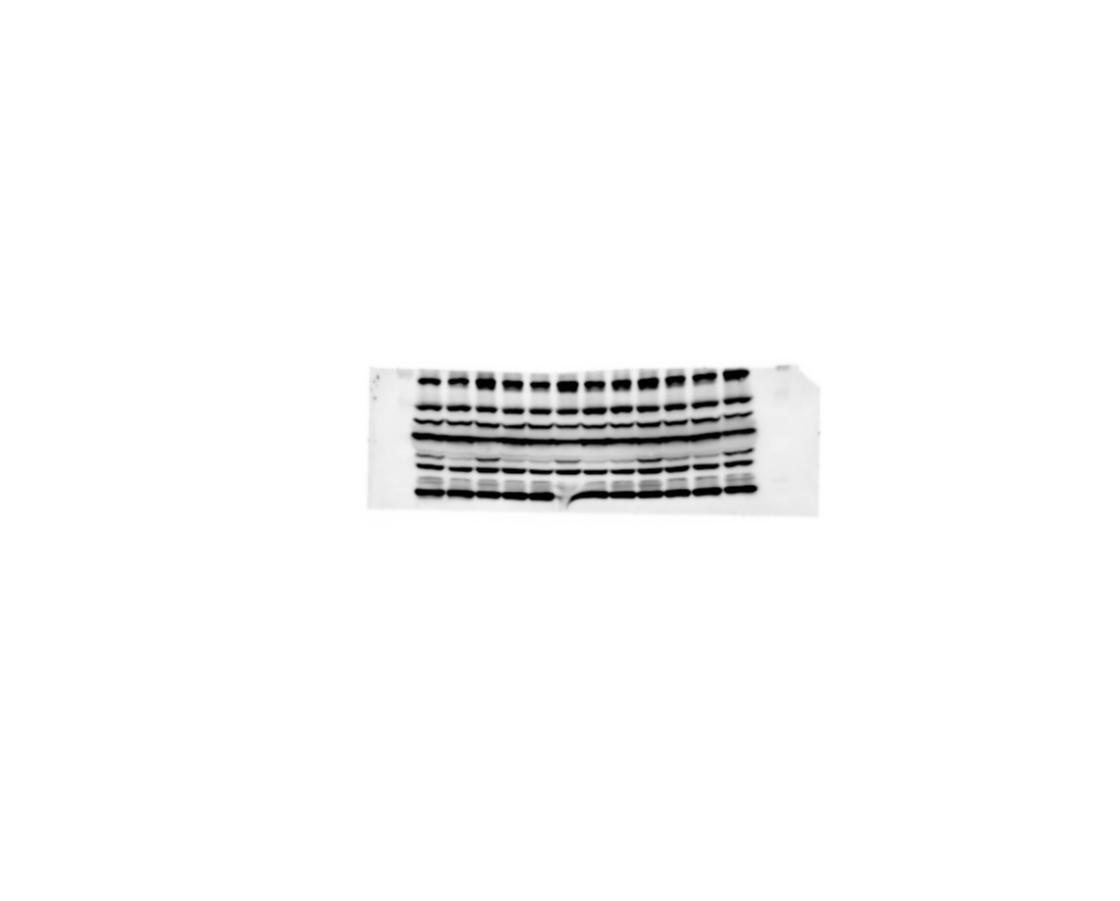

Supplement: Supplementary file 3 [file DataSheet1.ZIP › Original western blot images/Low nitrate diet followed by high nitrate water/Sialin_Low-high nitrate.tif]

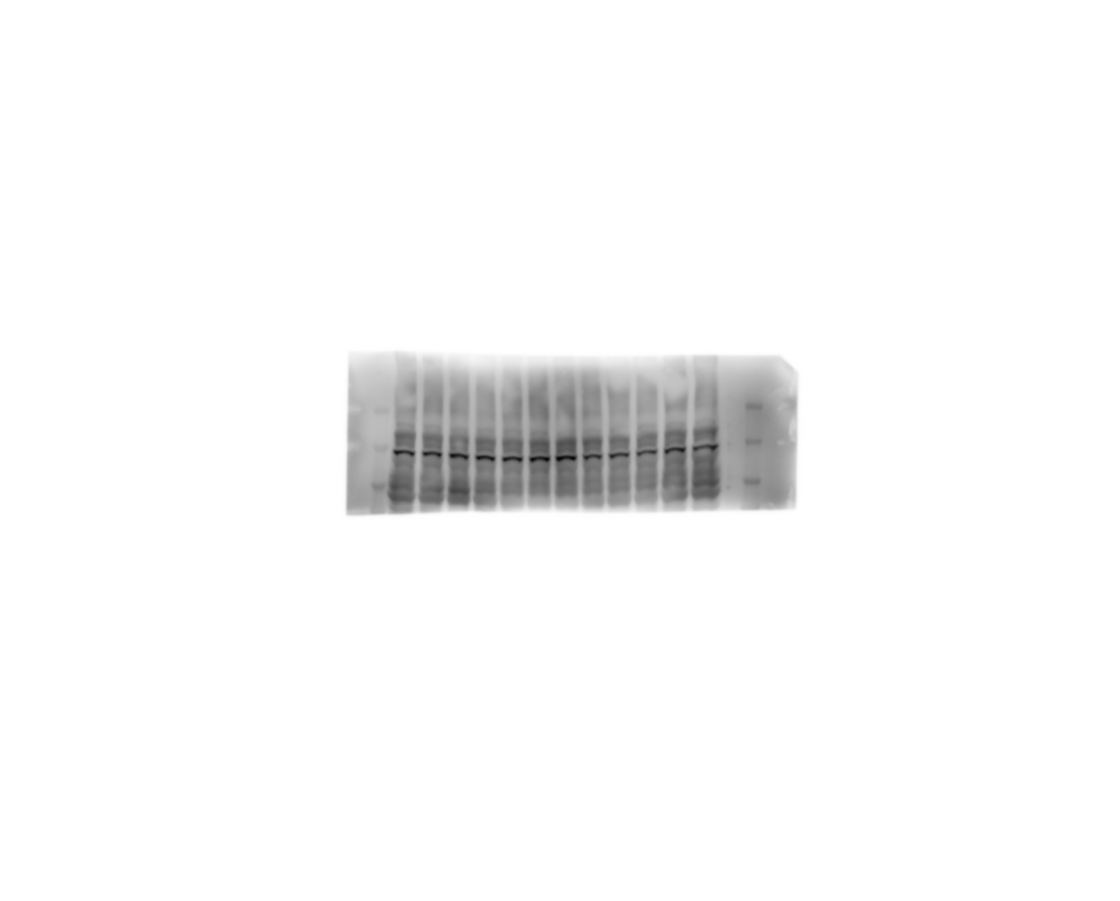

Supplement: Supplementary file 3 [file DataSheet1.ZIP › Original western blot images/Low nitrate diet followed by high nitrate water/XOR_Low-high nitrate.tif]

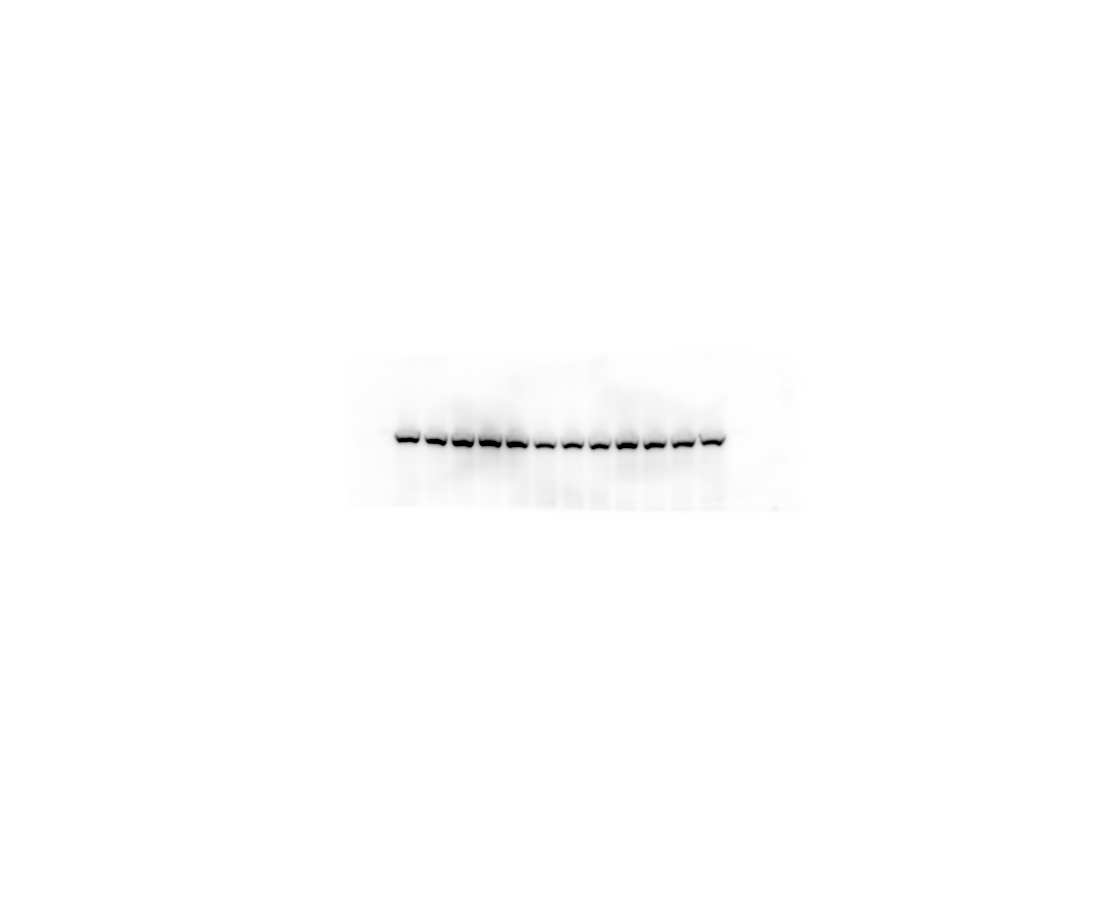

Supplement: Supplementary file 3 [file DataSheet1.ZIP › Original western blot images/Low nitrate diet/eNOS_Low nitrate.tif]

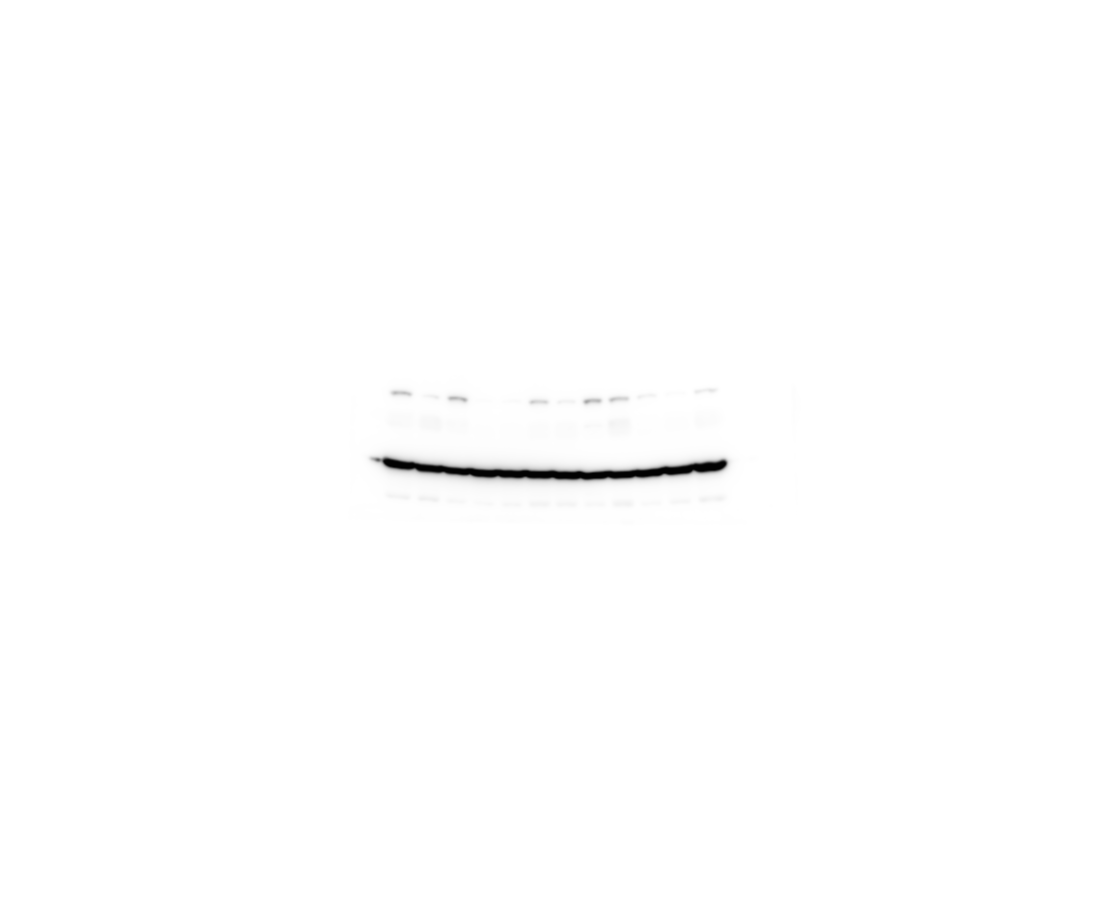

Supplement: Supplementary file 3 [file DataSheet1.ZIP › Original western blot images/Low nitrate diet/GAPDH_Low nitrate.tif]

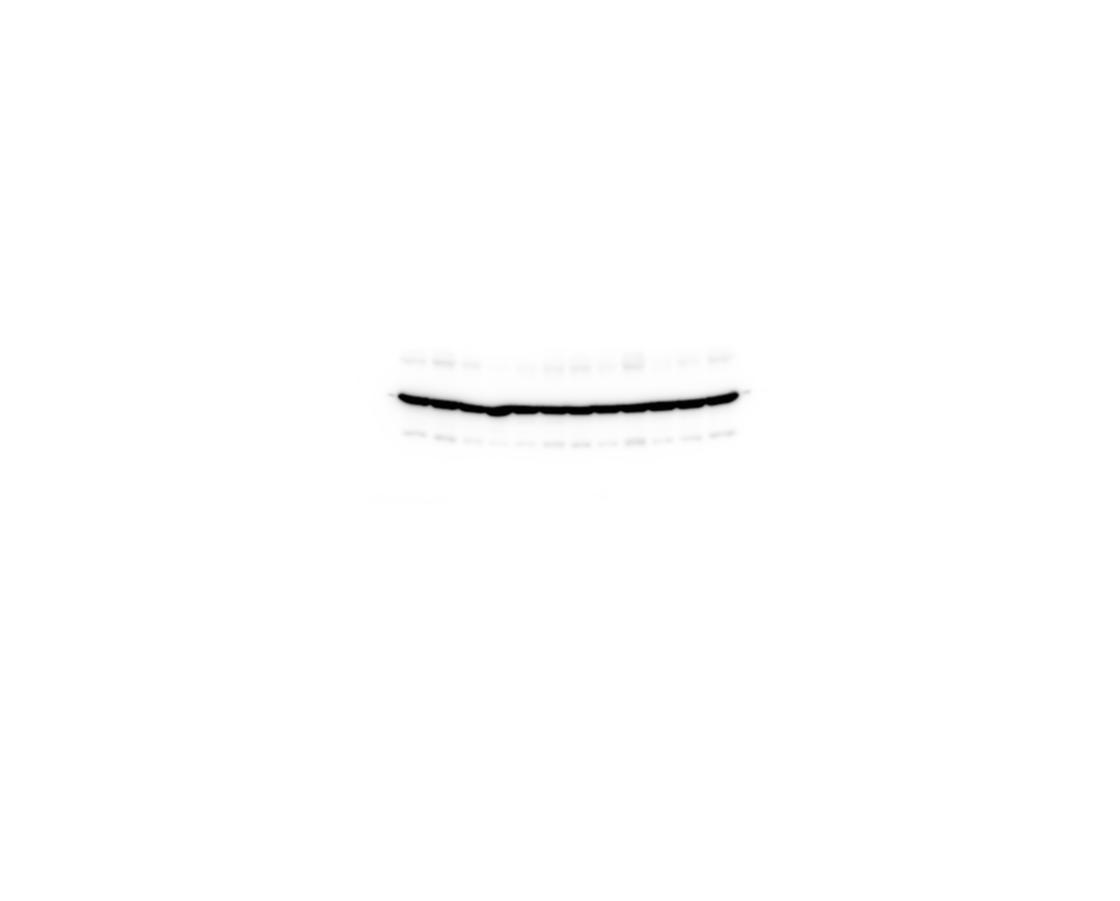

Supplement: Supplementary file 3 [file DataSheet1.ZIP › Original western blot images/Low nitrate diet/GAPDH-2_Low nitrate.tif]

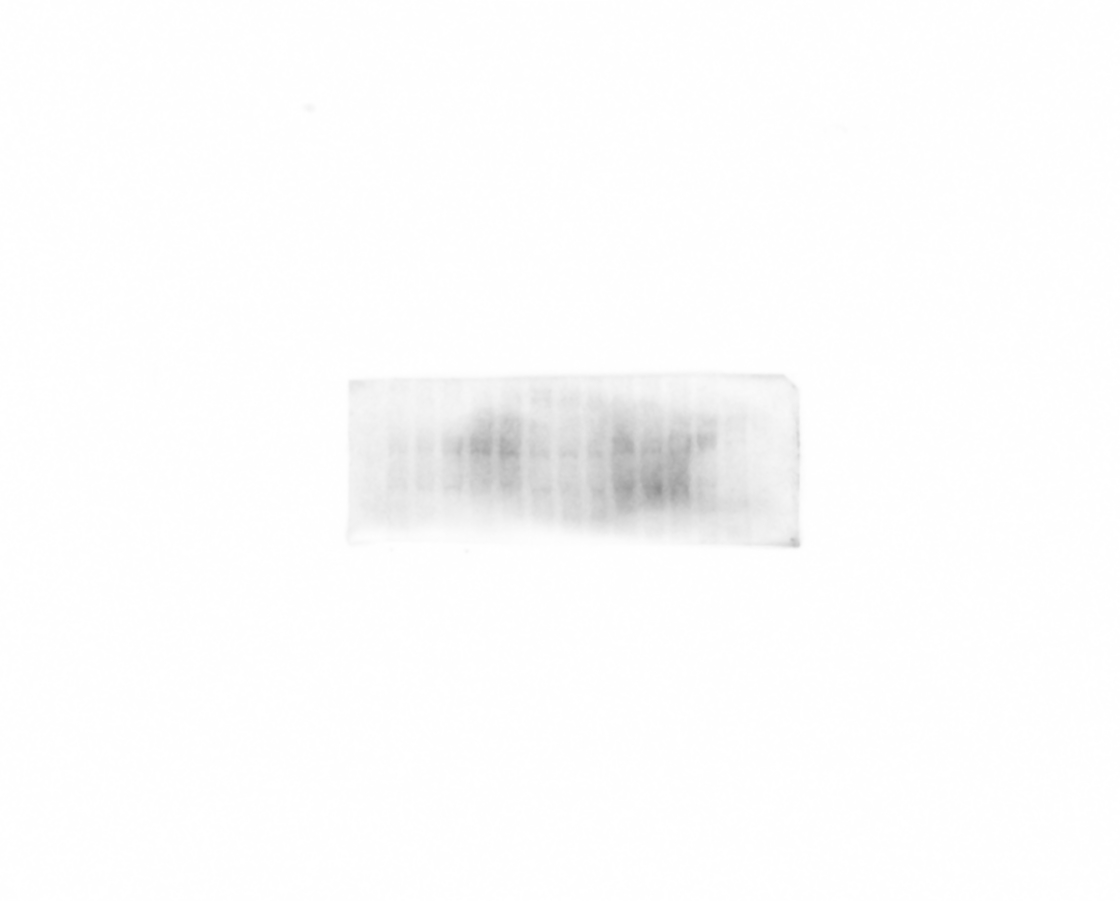

Supplement: Supplementary file 3 [file DataSheet1.ZIP › Original western blot images/Low nitrate diet/iNOS_Low nitrate.tif]

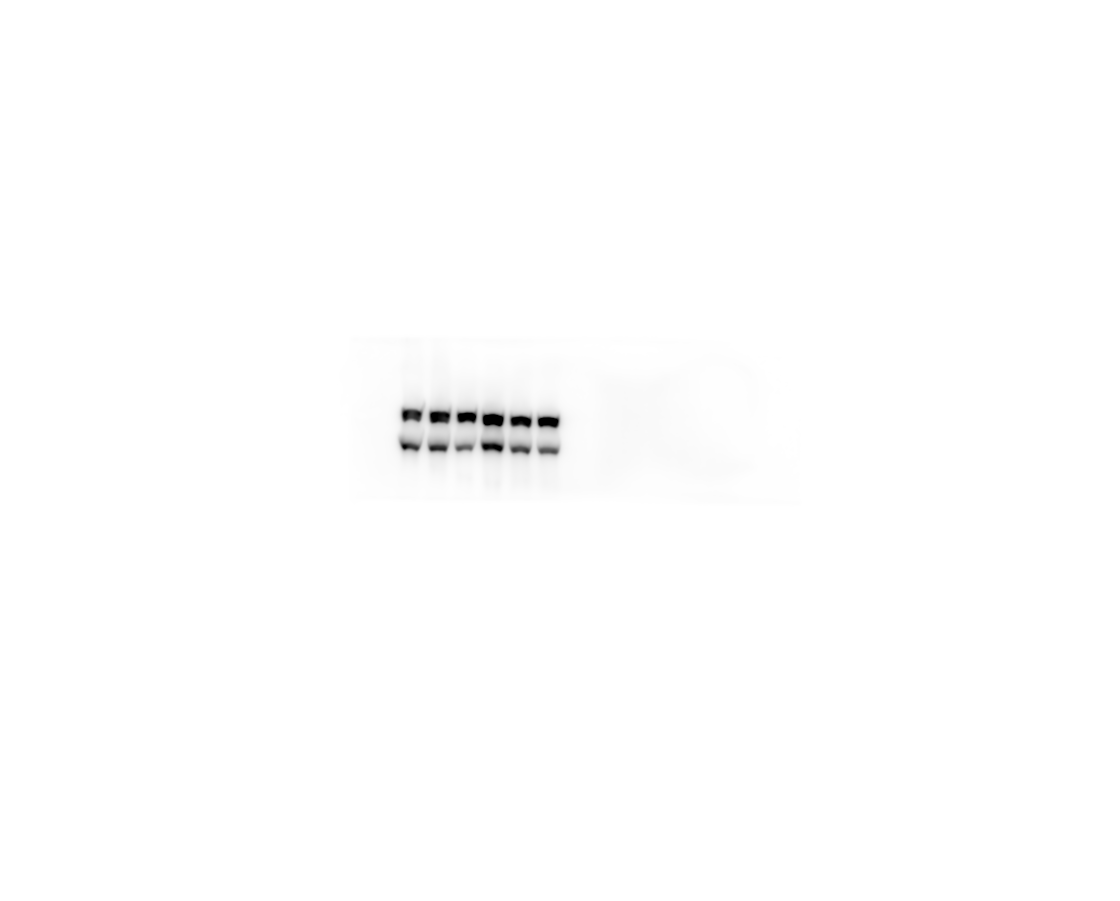

Supplement: Supplementary file 3 [file DataSheet1.ZIP › Original western blot images/Low nitrate diet/nNOS_Low nitrate.tif]

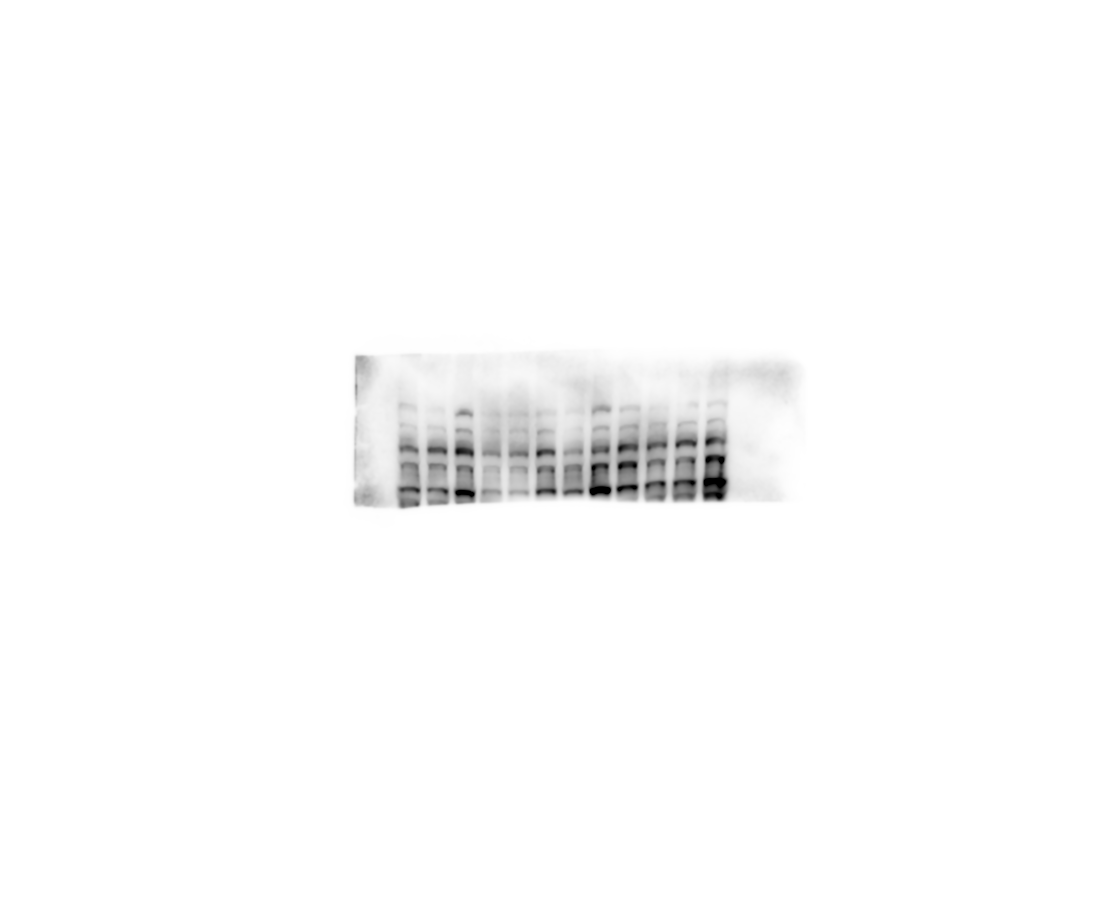

Supplement: Supplementary file 3 [file DataSheet1.ZIP › Original western blot images/Low nitrate diet/p-eNOSSer1177_Low nitrate.tif]

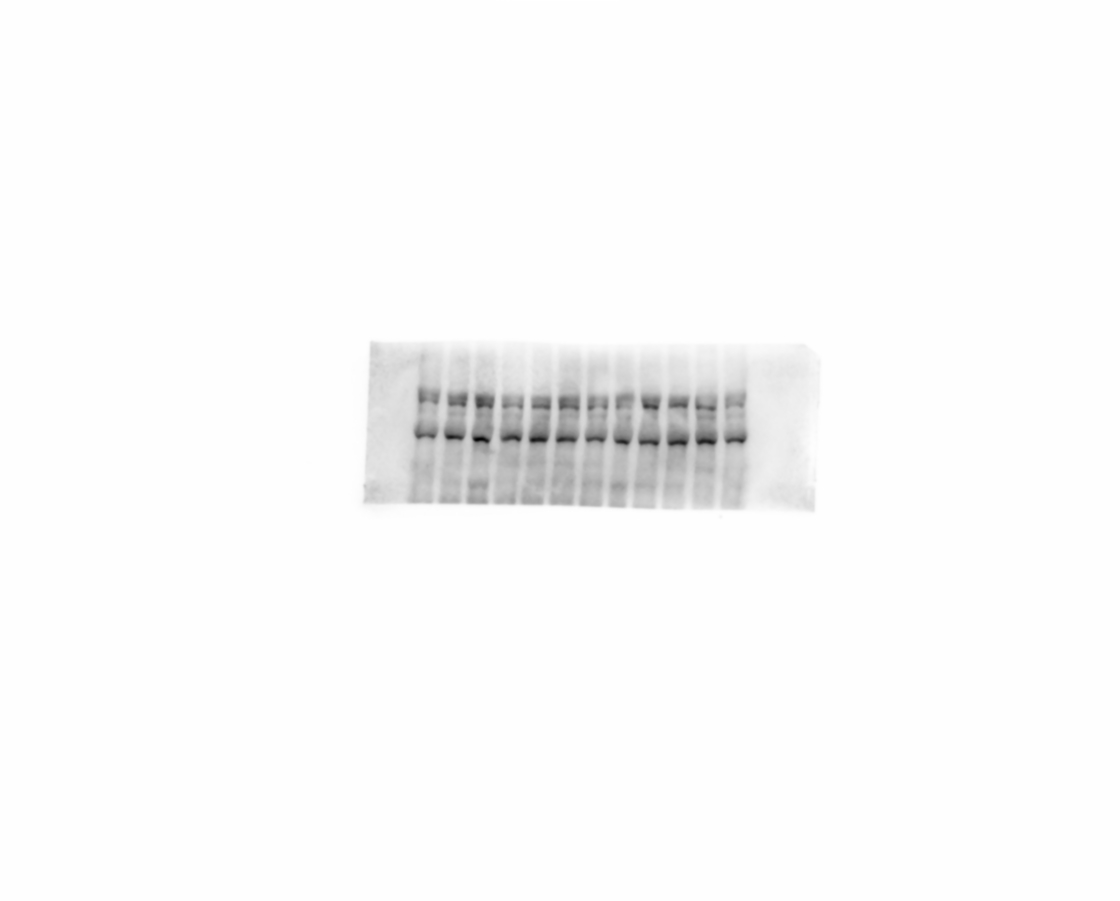

Supplement: Supplementary file 3 [file DataSheet1.ZIP › Original western blot images/Low nitrate diet/p-eNOSThr495_Low nitrate.tif]

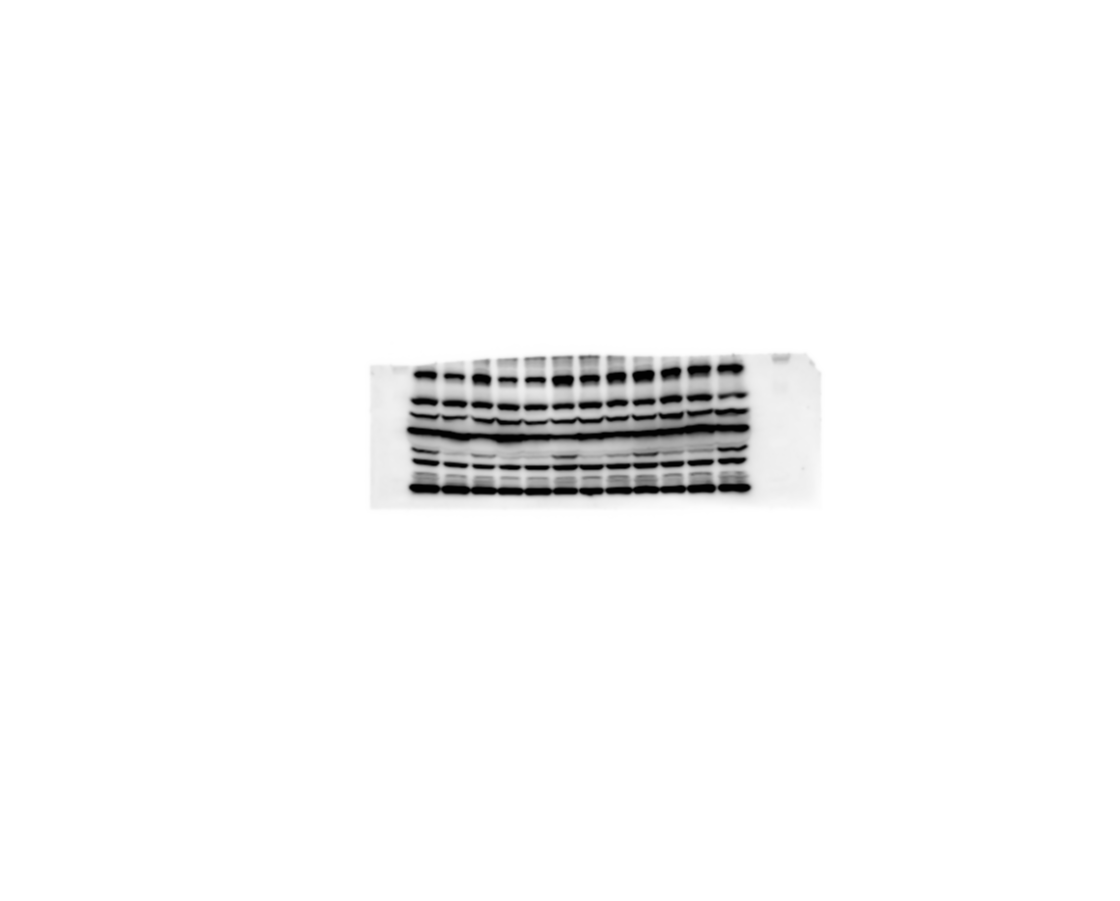

Supplement: Supplementary file 3 [file DataSheet1.ZIP › Original western blot images/Low nitrate diet/Sialin_Low nitrate.tif]

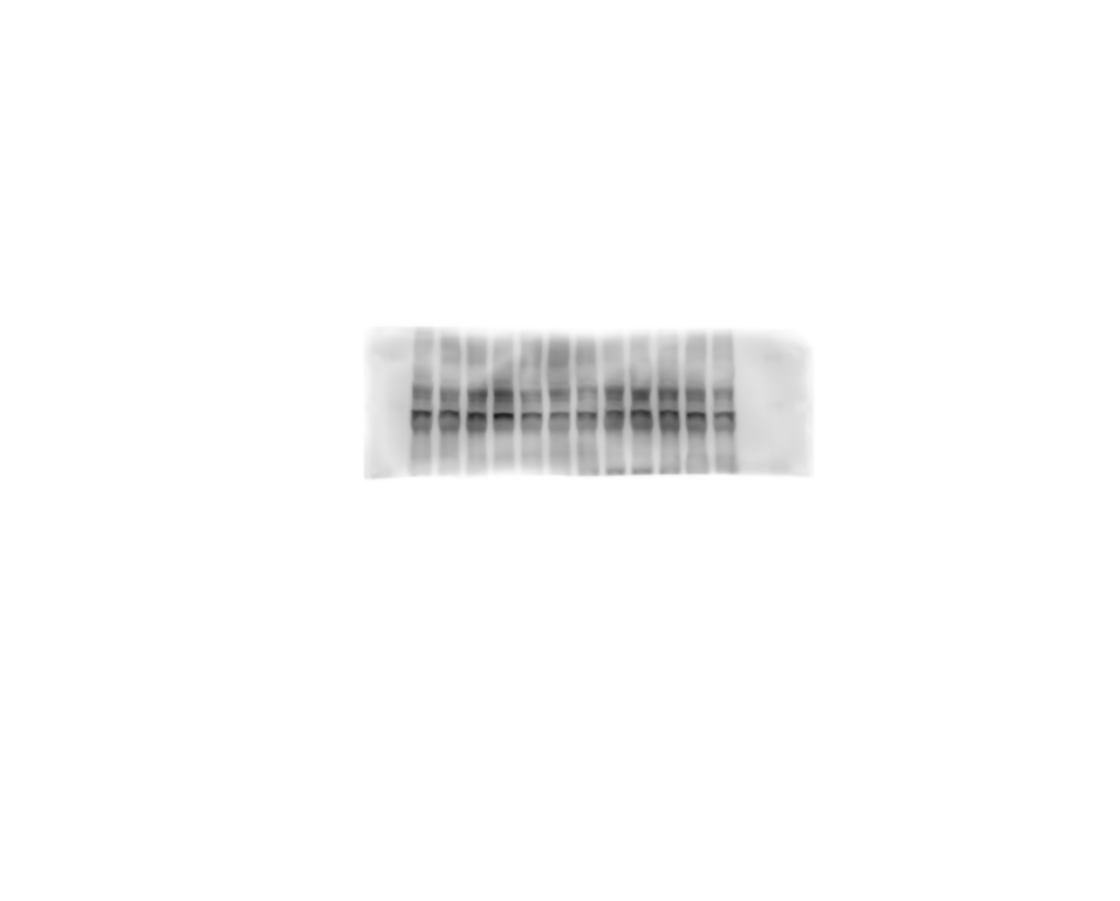

Supplement: Supplementary file 3 [file DataSheet1.ZIP › Original western blot images/Low nitrate diet/XOR_Low nitrate.tif]

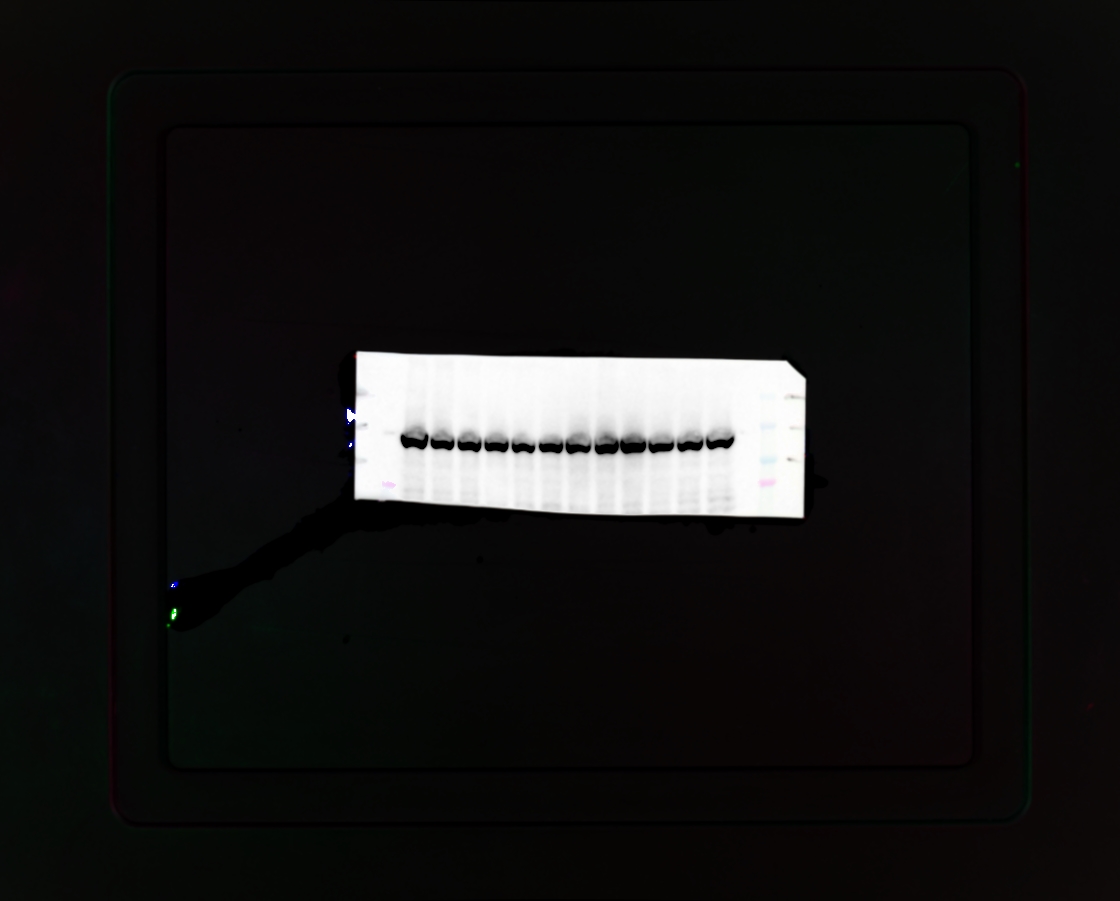

Supplement: Supplementary file 3 [file DataSheet1.ZIP › Original western blot images/Protein bands and protein marker/eNOS+Marker.jpg]

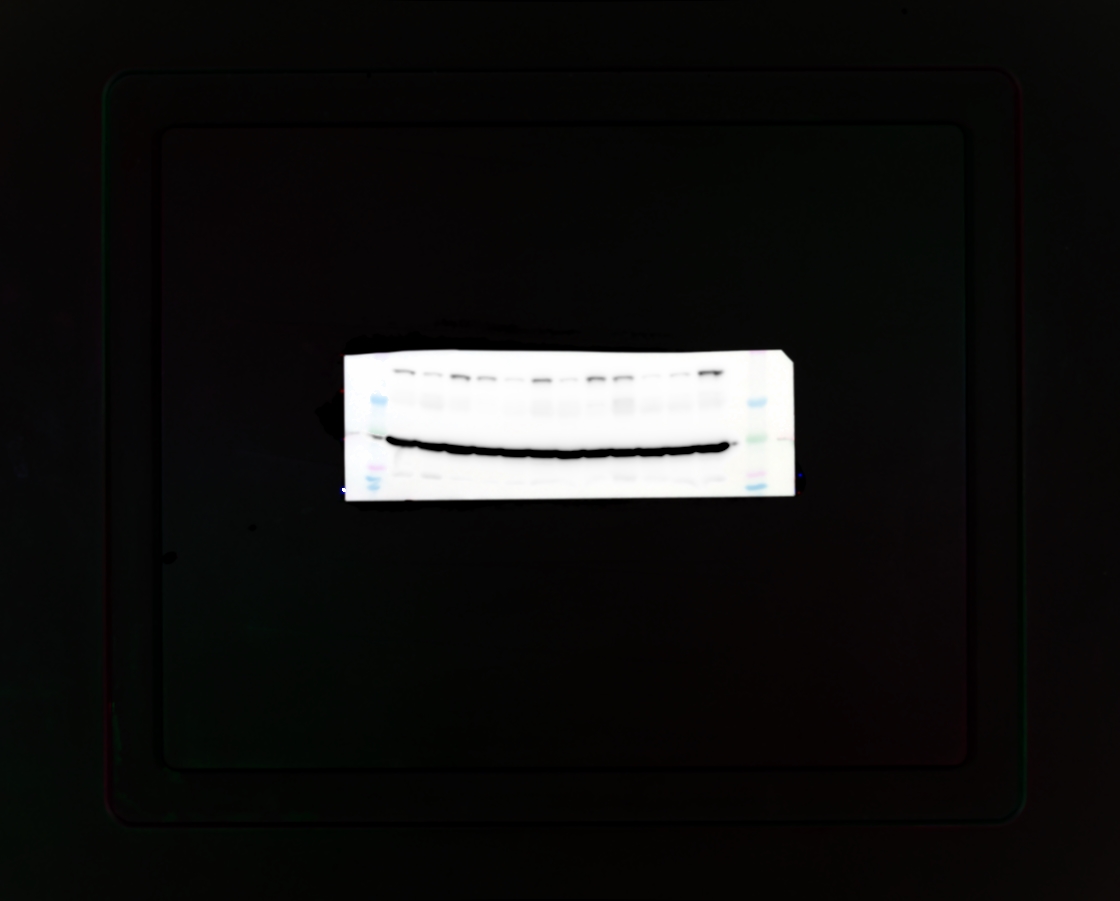

Supplement: Supplementary file 3 [file DataSheet1.ZIP › Original western blot images/Protein bands and protein marker/GAPDH+Marker.jpg]

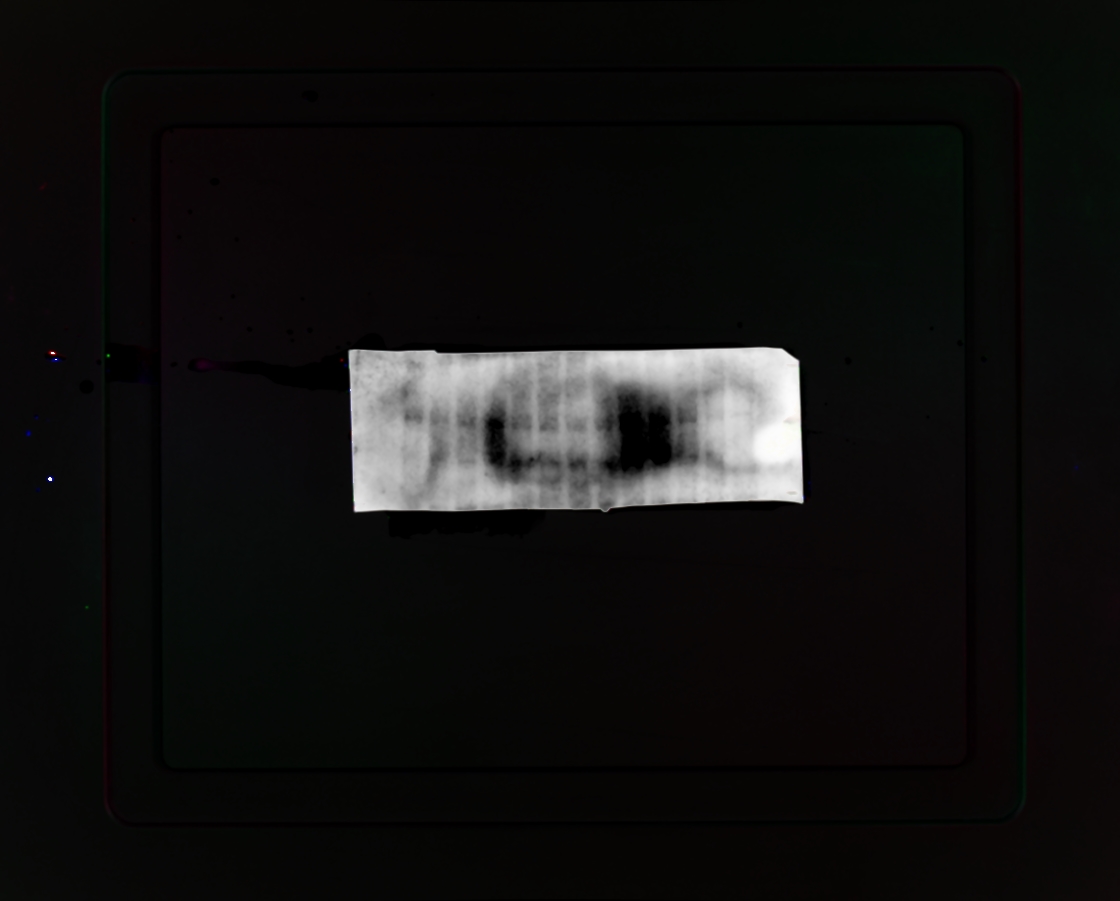

Supplement: Supplementary file 3 [file DataSheet1.ZIP › Original western blot images/Protein bands and protein marker/iNOS+Marker.jpg]

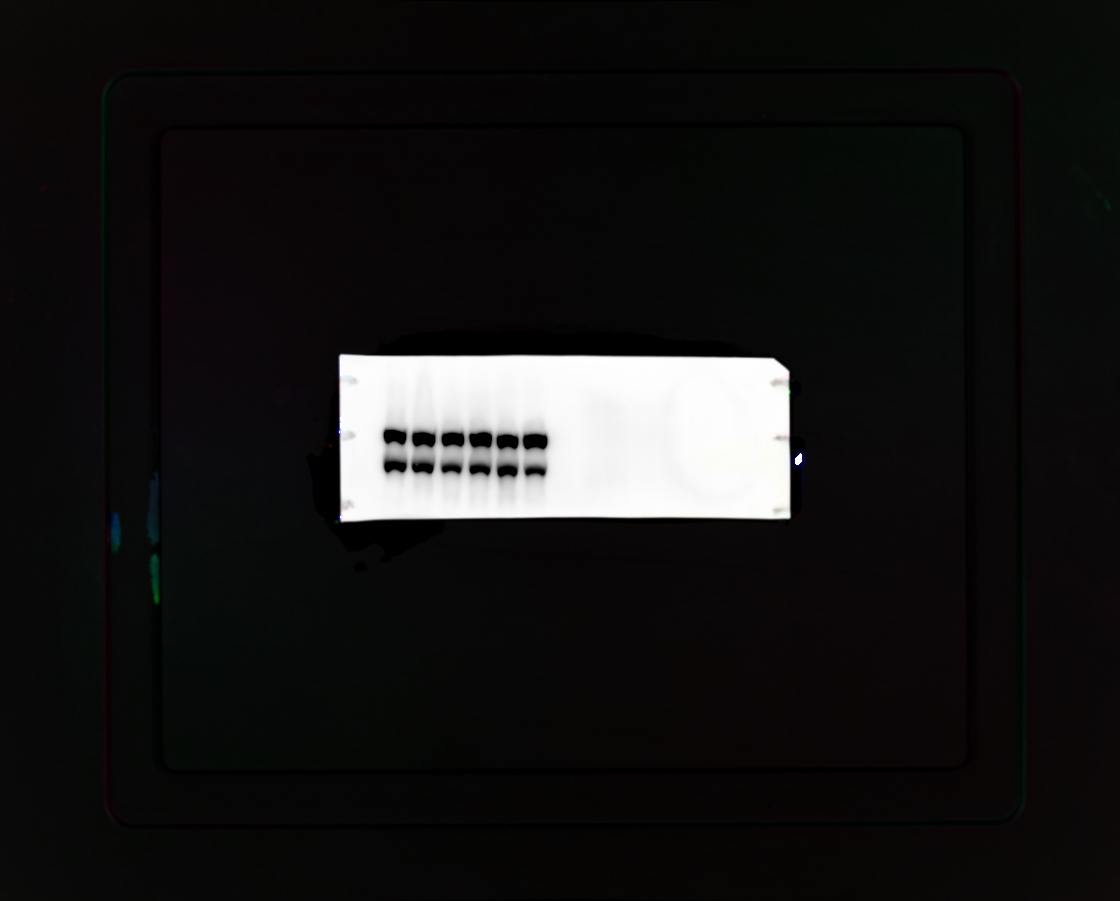

Supplement: Supplementary file 3 [file DataSheet1.ZIP › Original western blot images/Protein bands and protein marker/nNOS+Marker.jpg]

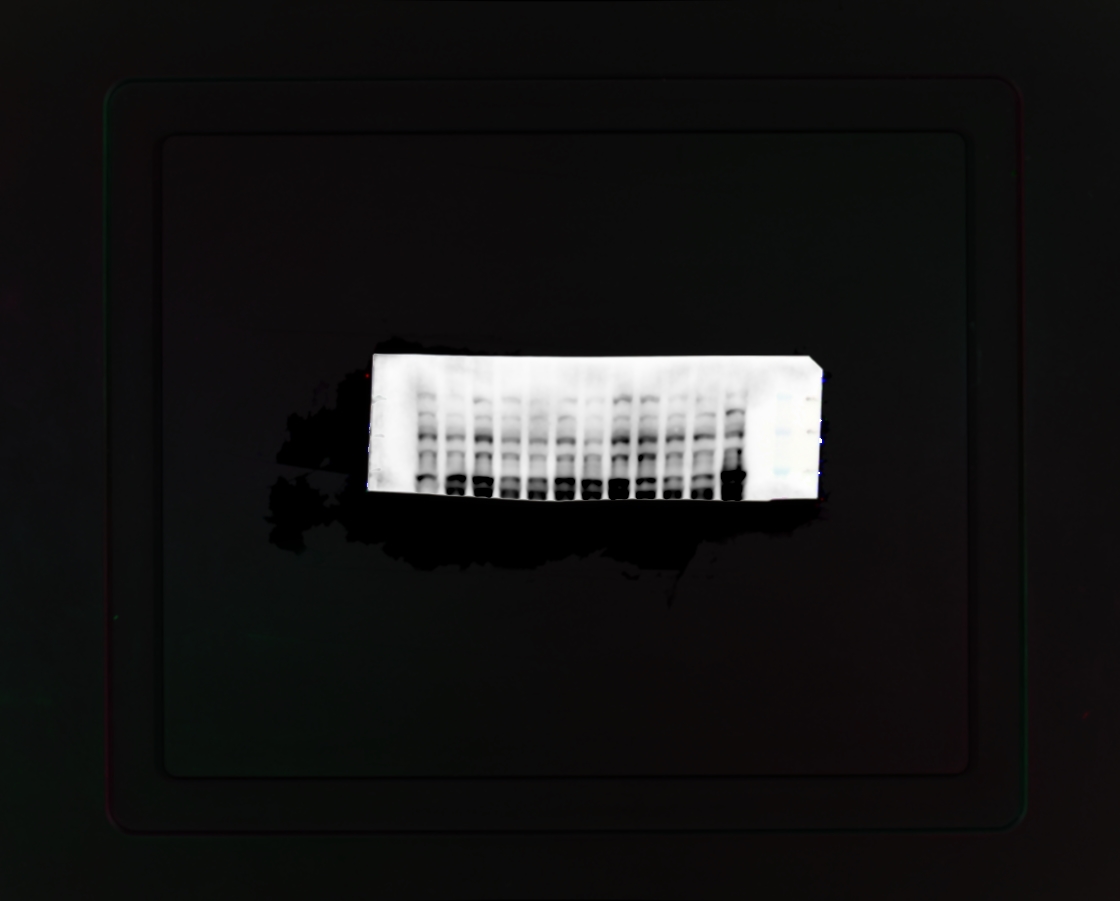

Supplement: Supplementary file 3 [file DataSheet1.ZIP › Original western blot images/Protein bands and protein marker/p-eNOSSer1177+Marker.jpg]

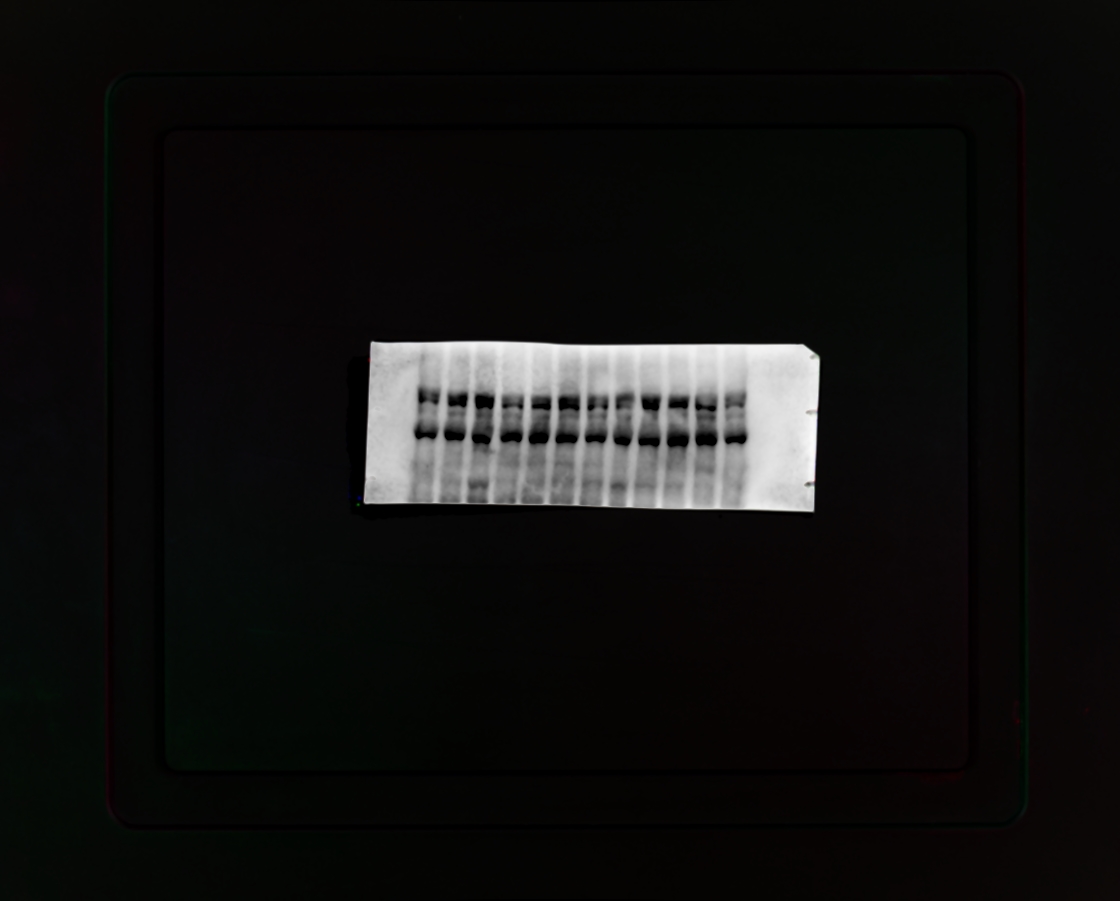

Supplement: Supplementary file 3 [file DataSheet1.ZIP › Original western blot images/Protein bands and protein marker/p-eNOSThr495+Marker.jpg]

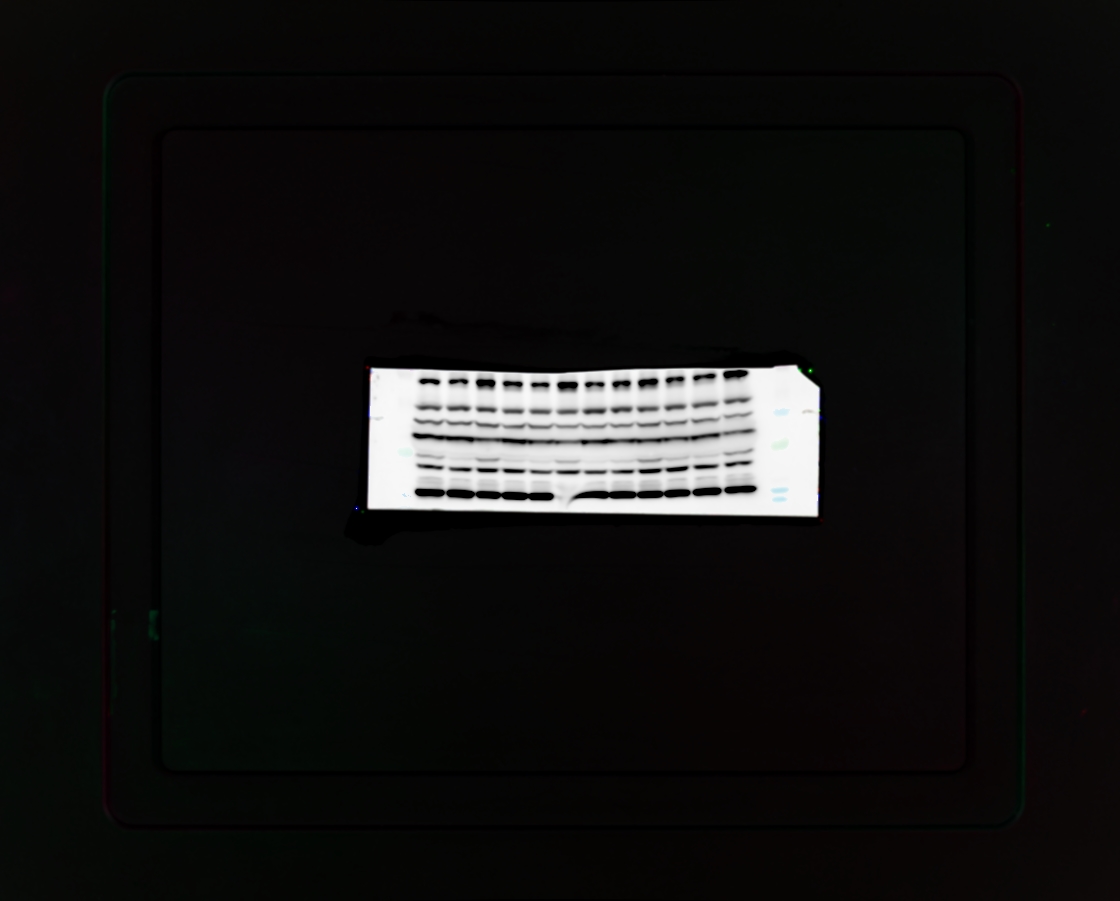

Supplement: Supplementary file 3 [file DataSheet1.ZIP › Original western blot images/Protein bands and protein marker/Sialin+Marker.jpg]

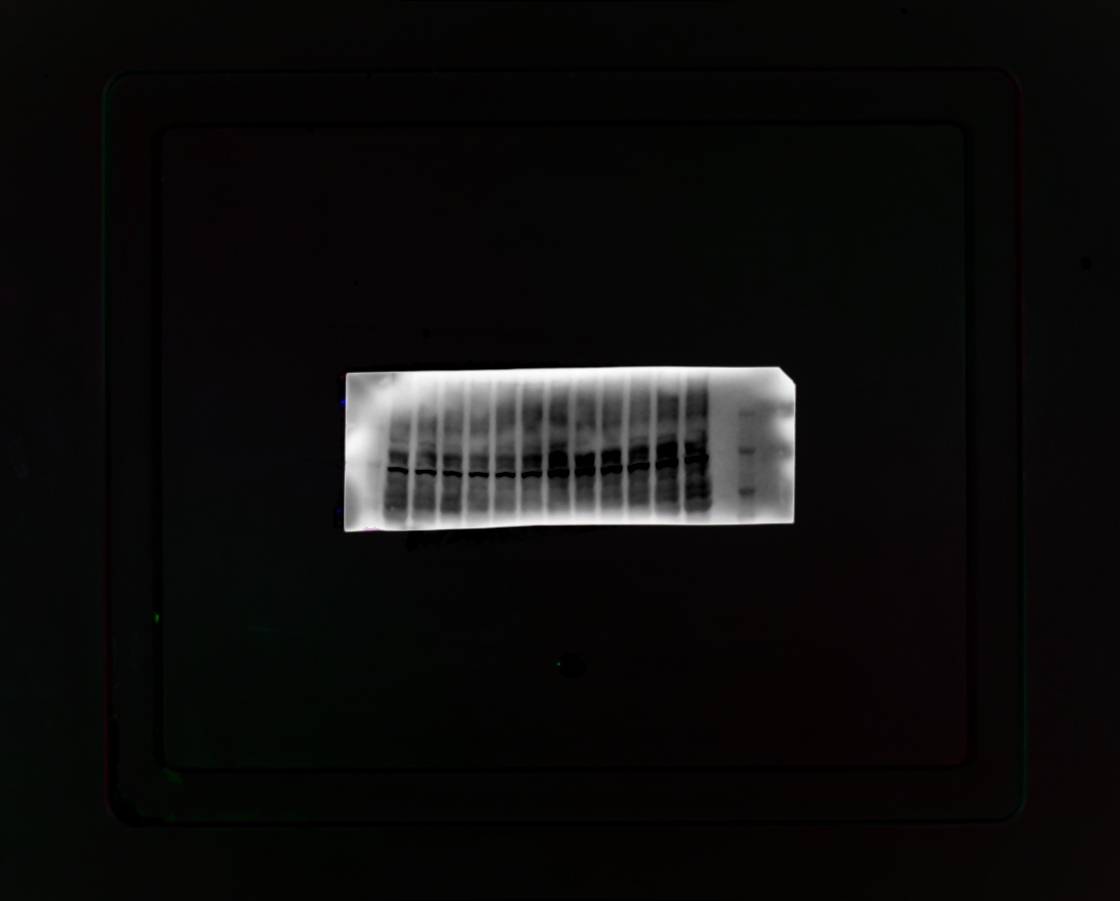

Supplement: Supplementary file 3 [file DataSheet1.ZIP › Original western blot images/Protein bands and protein marker/XOR+Marker.jpg]
